# Supplementary figures and images for: Dedicated chaperones coordinate co-translational regulation of ribosomal protein production with ribosome assembly to preserve proteostasis
Source: eLife. 2022 Mar 31;11:e74255. doi: 10.7554/eLife.74255 (PMC8970588; doi:10.7554/eLife.74255)

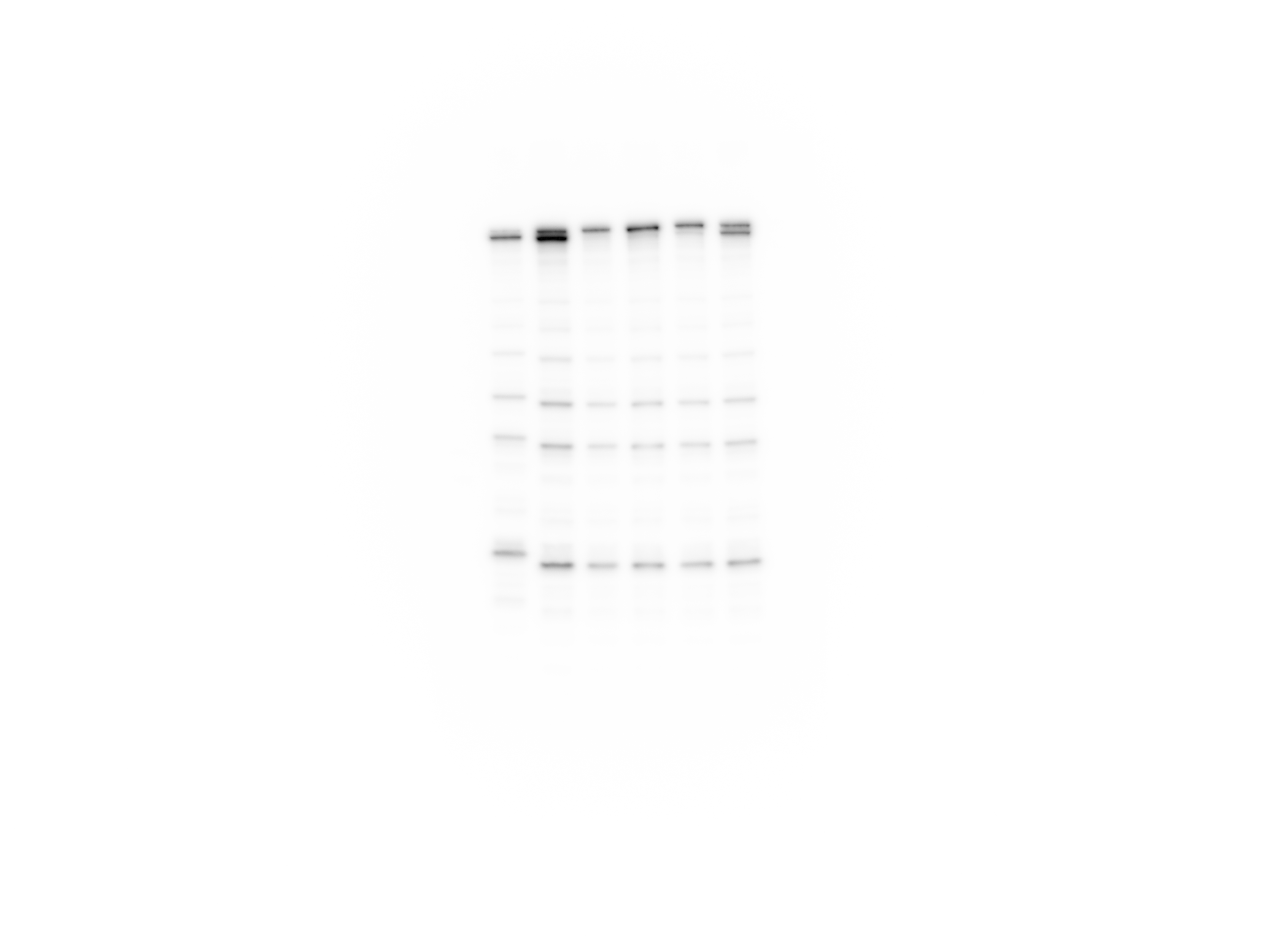

Supplement: Figure 2—source data 1. [file elife-74255-fig2-data1.zip › Figure 2B - source 1.tif]

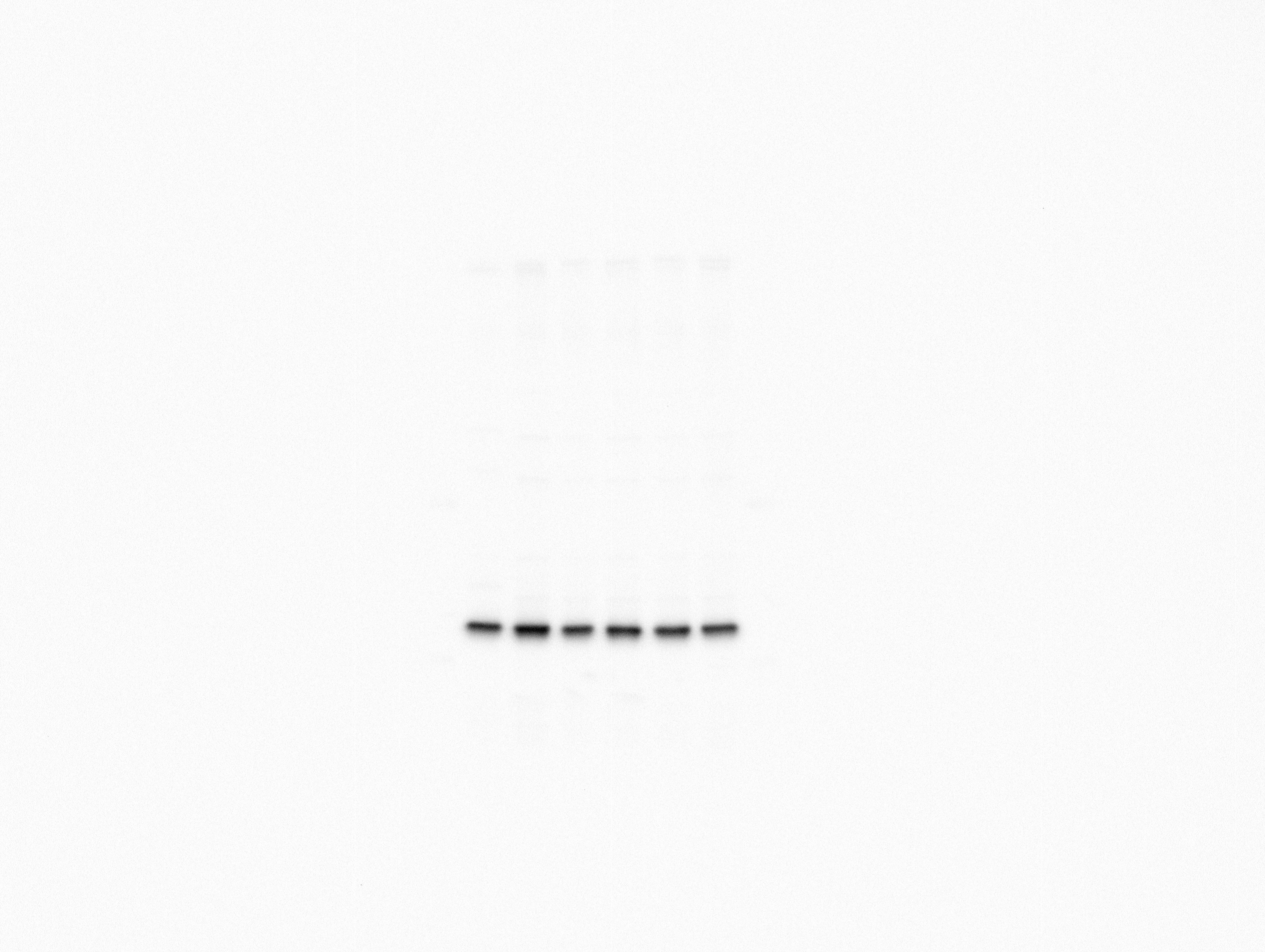

Supplement: Figure 2—source data 1. [file elife-74255-fig2-data1.zip › Figure 2B - source 2.tif]

# Figure 2B - source data

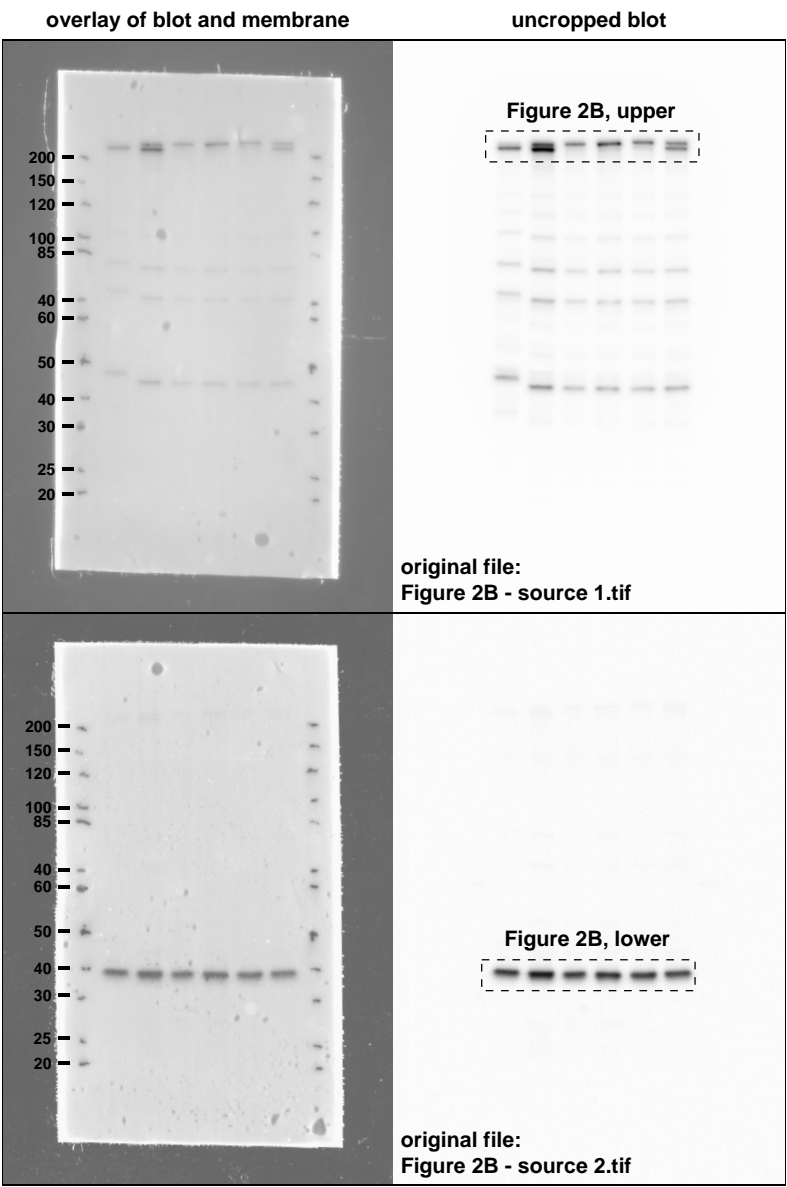

Supplement: Figure 2—source data 1. [file elife-74255-fig2-data1.zip › Figure 2B - source data.pdf]

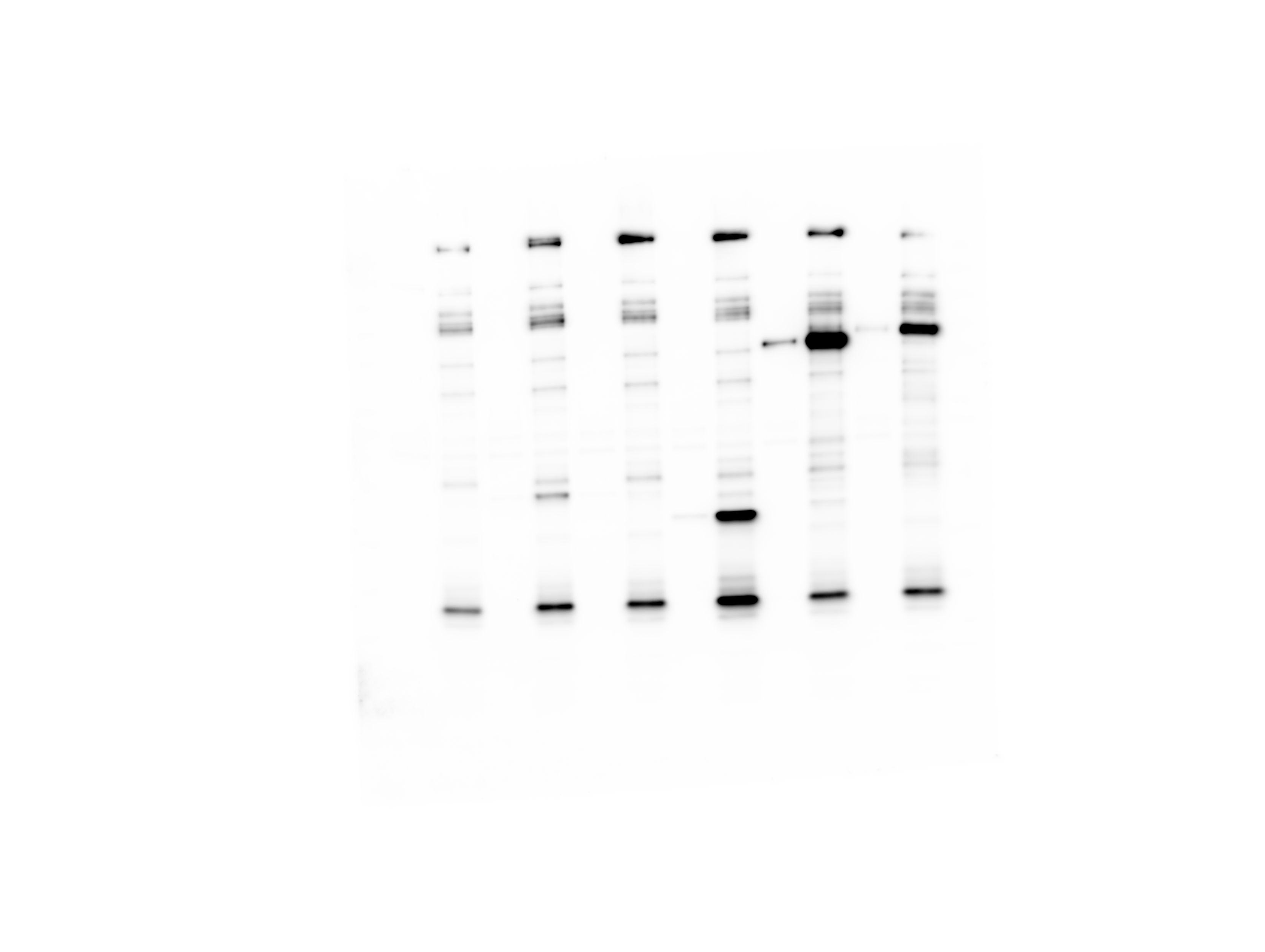

Supplement: Figure 3—source data 1. [file elife-74255-fig3-data1.zip › Figure 3AB - source 154C GFP IP.tif]

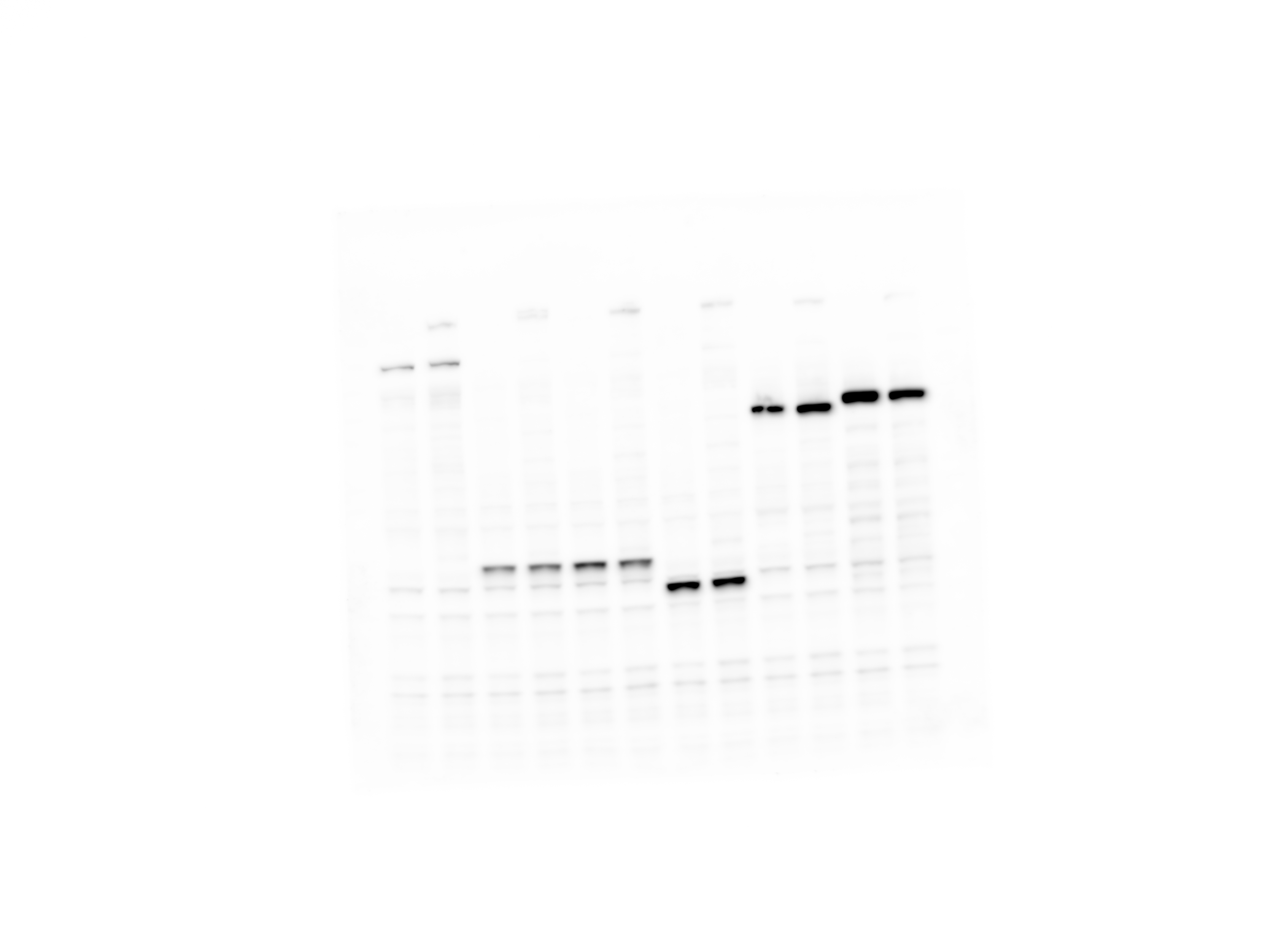

Supplement: Figure 3—source data 1. [file elife-74255-fig3-data1.zip › Figure 3AB - source 154C GFP total.tif]

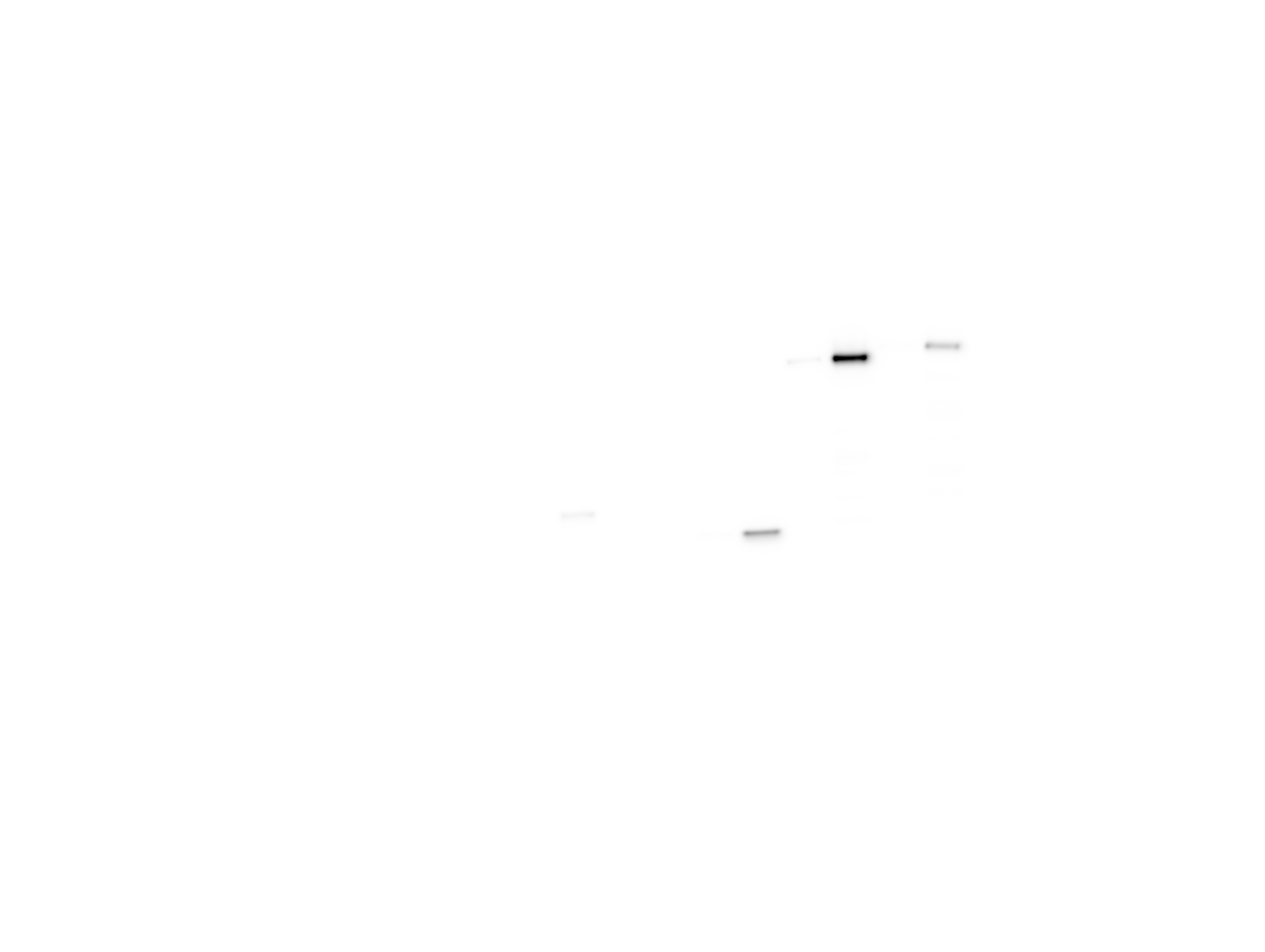

Supplement: Figure 3—source data 1. [file elife-74255-fig3-data1.zip › Figure 3AB - source 154C TAP IP.tif]

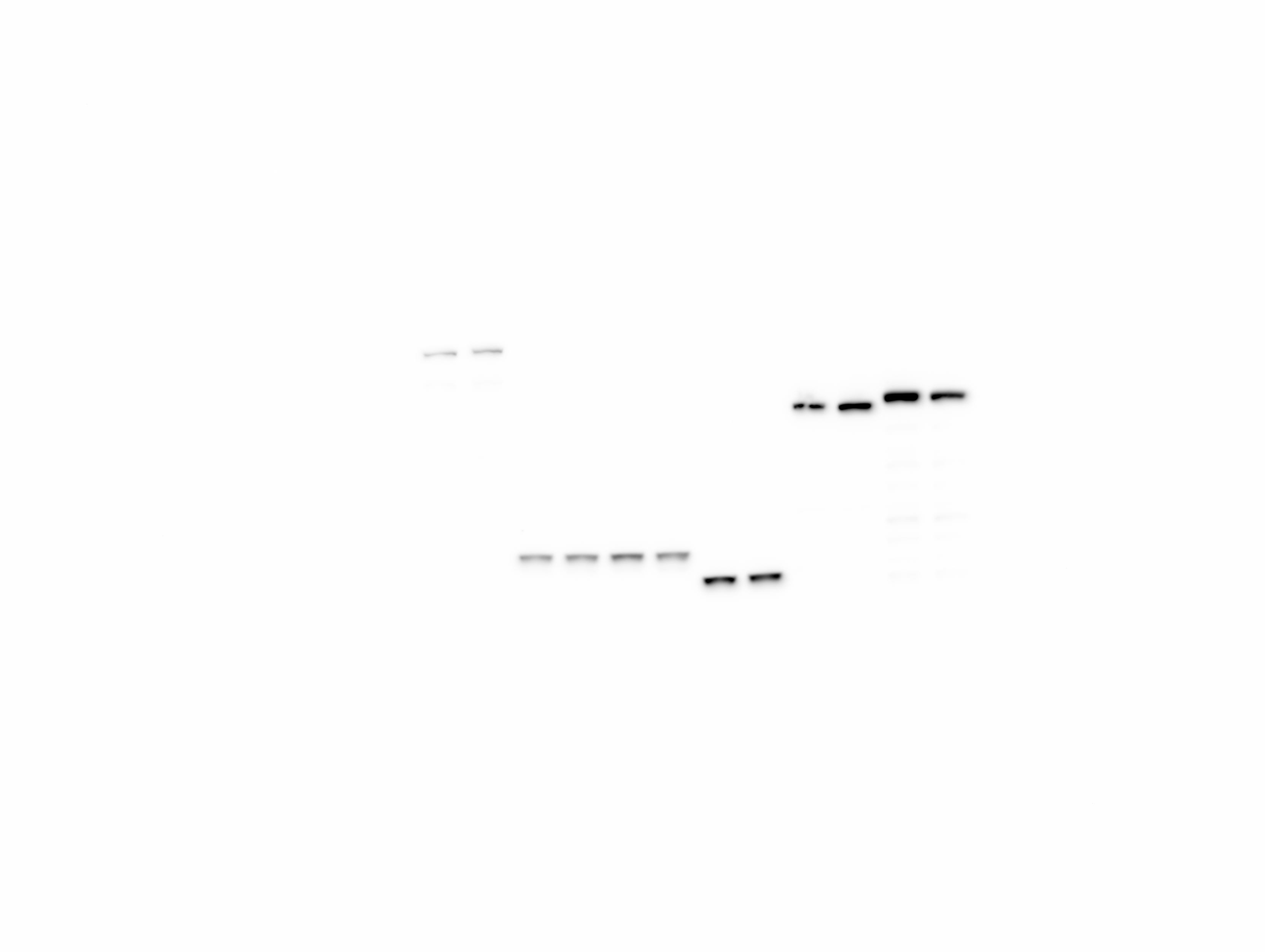

Supplement: Figure 3—source data 1. [file elife-74255-fig3-data1.zip › Figure 3AB - source 154C TAP total.tif]

# Figure 3AB - source data

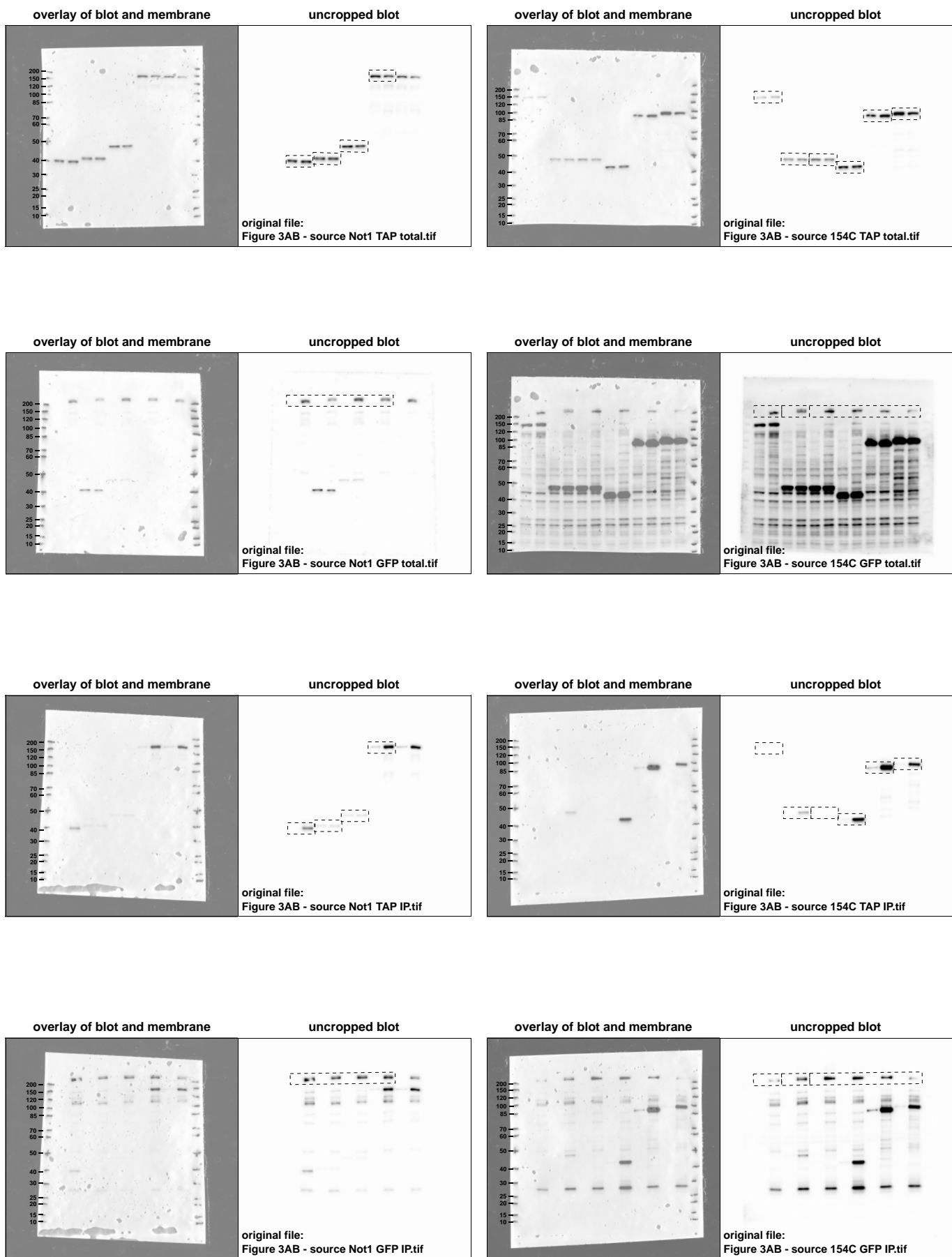

Supplement: Figure 3—source data 1. [file elife-74255-fig3-data1.zip › Figure 3AB - source data.pdf]

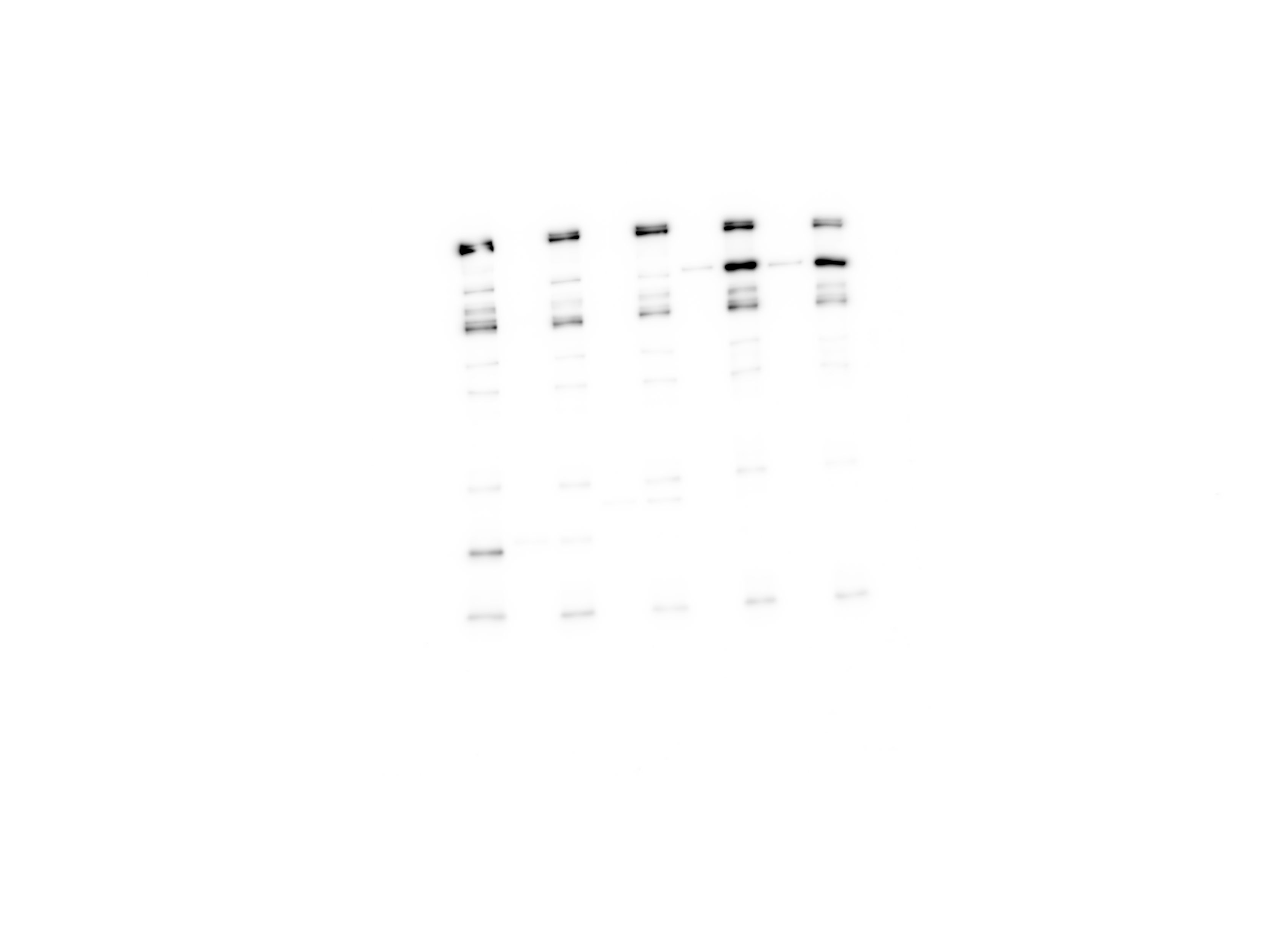

Supplement: Figure 3—source data 1. [file elife-74255-fig3-data1.zip › Figure 3AB - source Not1 GFP IP.tif]

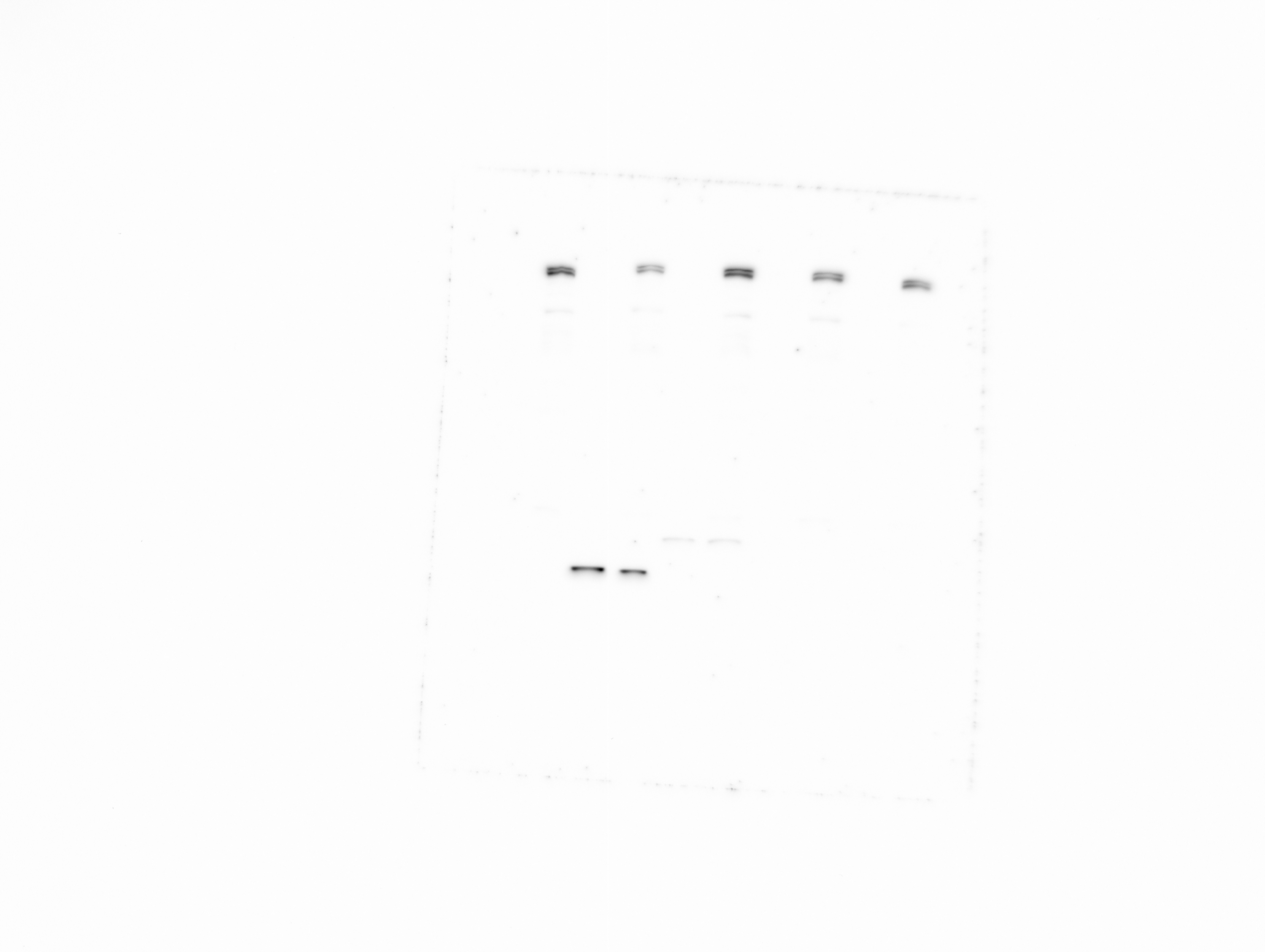

Supplement: Figure 3—source data 1. [file elife-74255-fig3-data1.zip › Figure 3AB - source Not1 GFP total.tif]

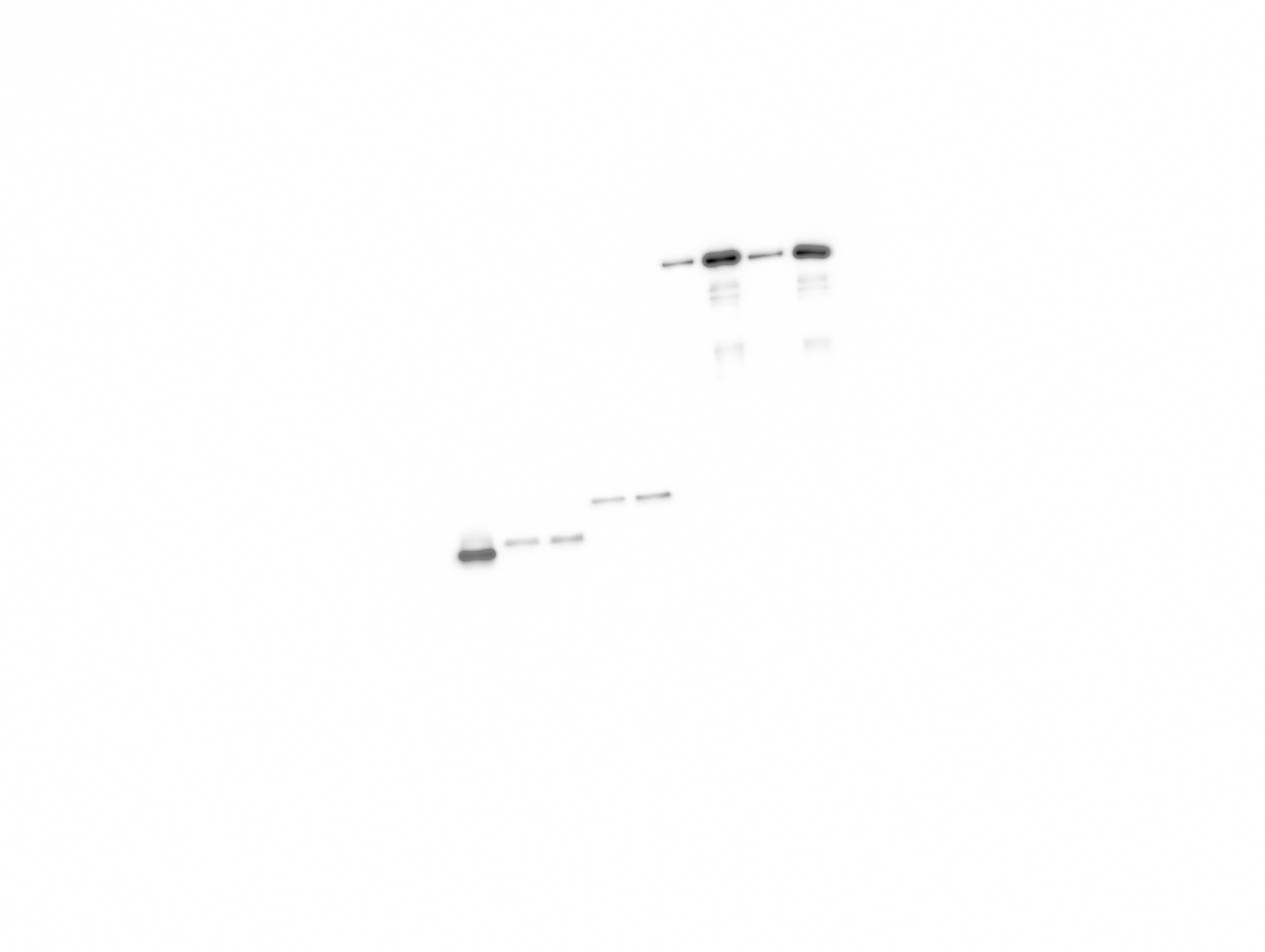

Supplement: Figure 3—source data 1. [file elife-74255-fig3-data1.zip › Figure 3AB - source Not1 TAP IP.tif]

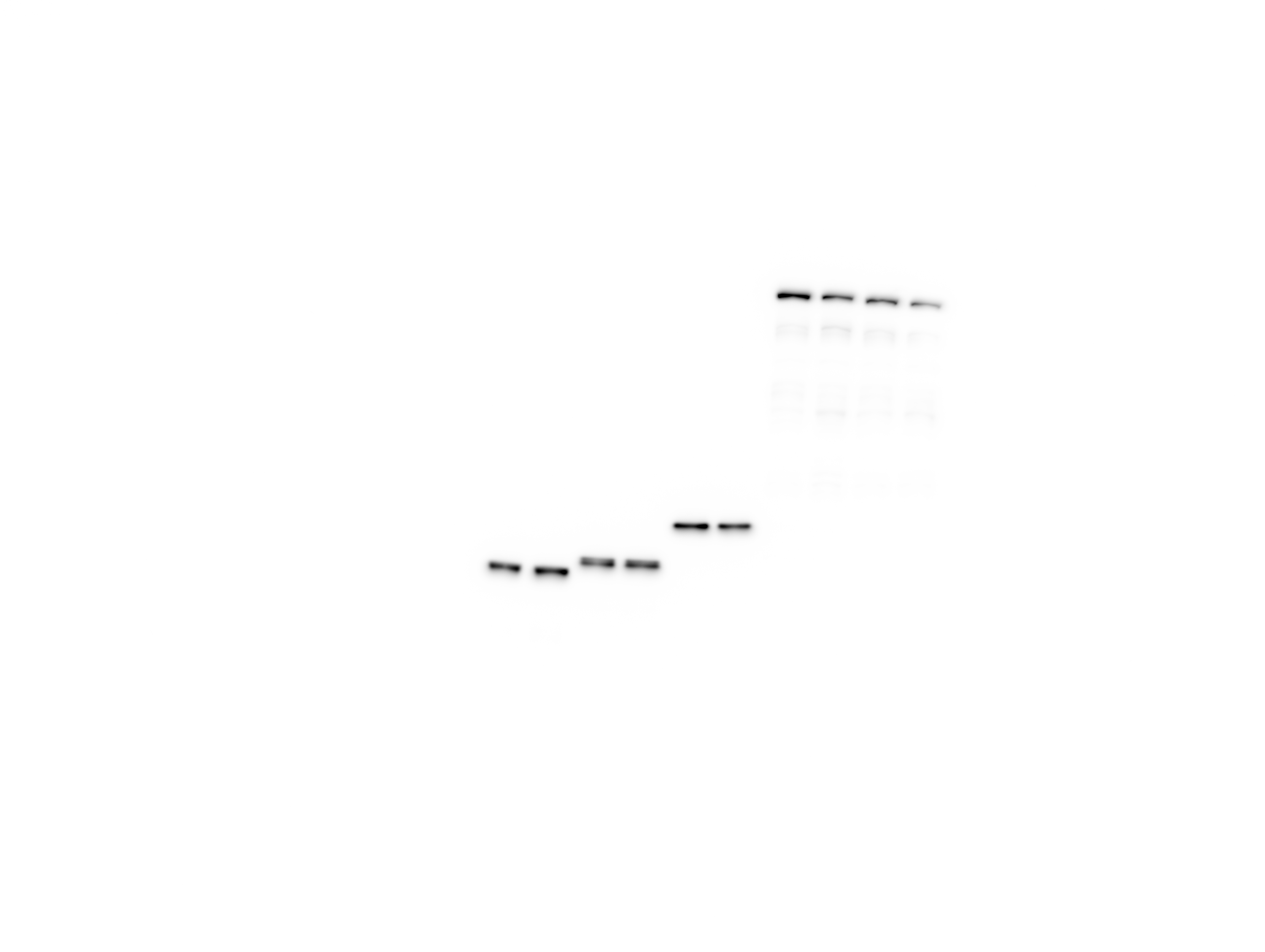

Supplement: Figure 3—source data 1. [file elife-74255-fig3-data1.zip › Figure 3AB - source Not1 TAP total.tif]

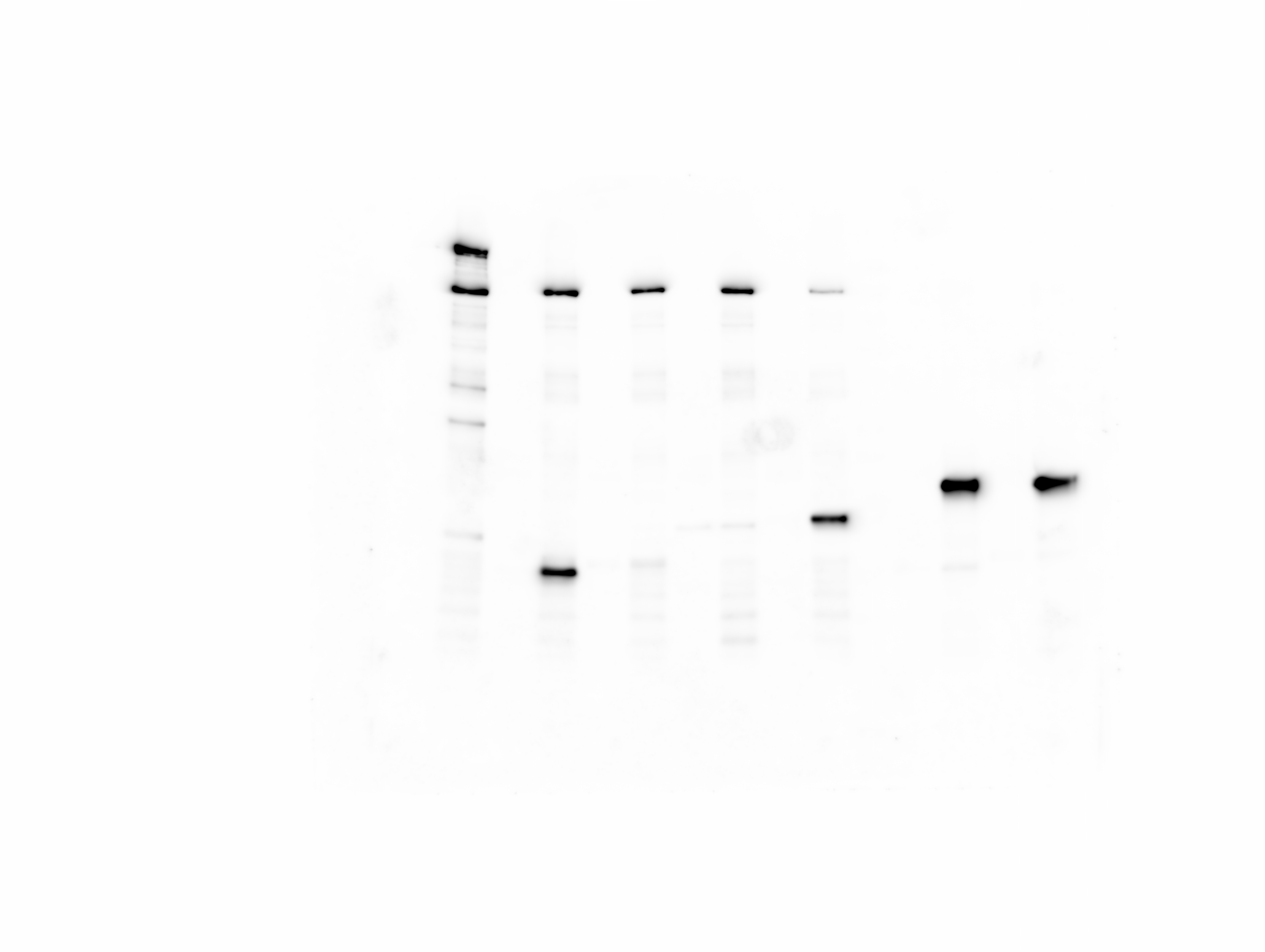

Supplement: Figure 3—source data 2. [file elife-74255-fig3-data2.zip › Figure 3C - source Caf130 GFP IP.tif]

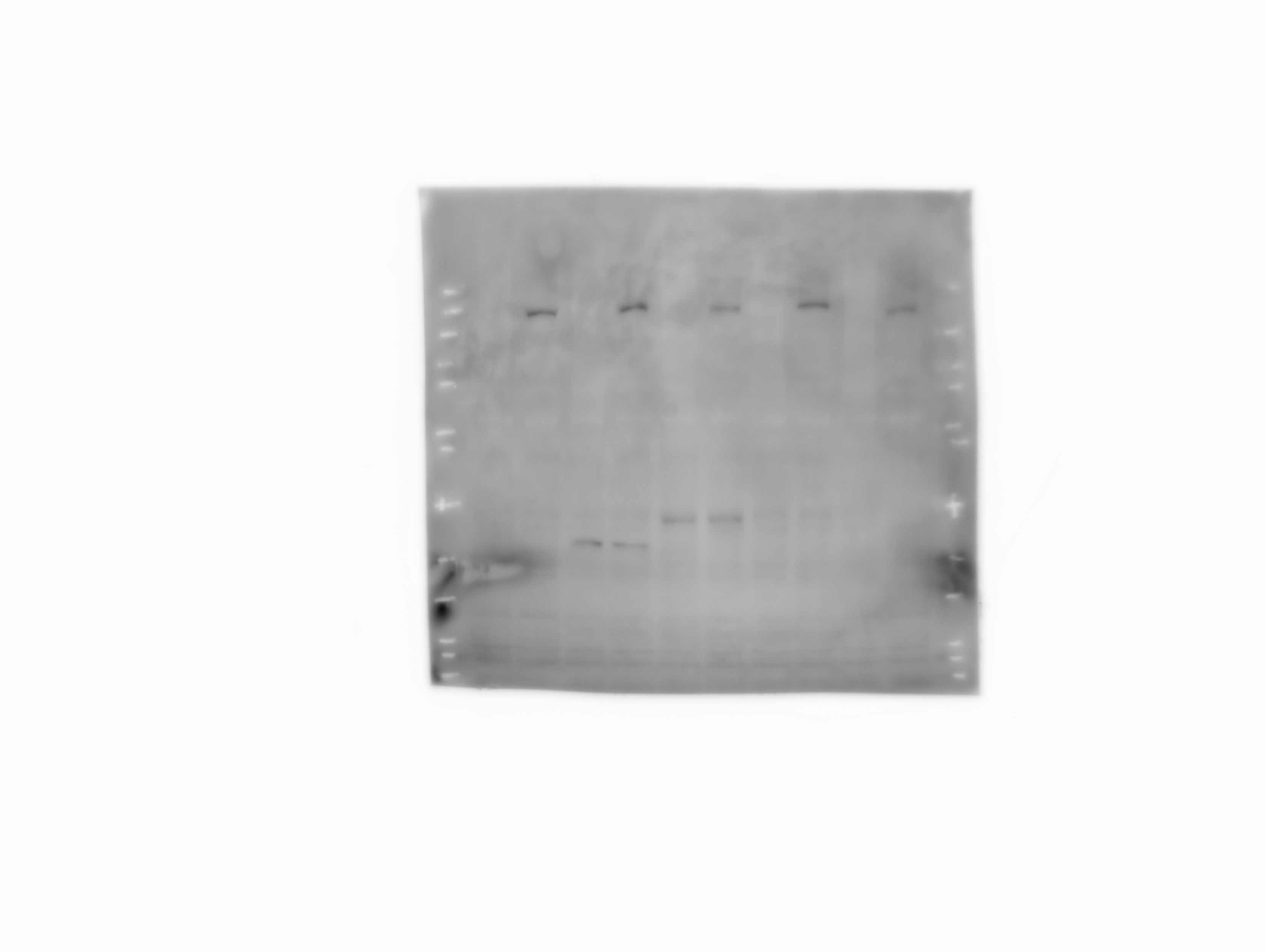

Supplement: Figure 3—source data 2. [file elife-74255-fig3-data2.zip › Figure 3C - source Caf130 GFP total.tif]

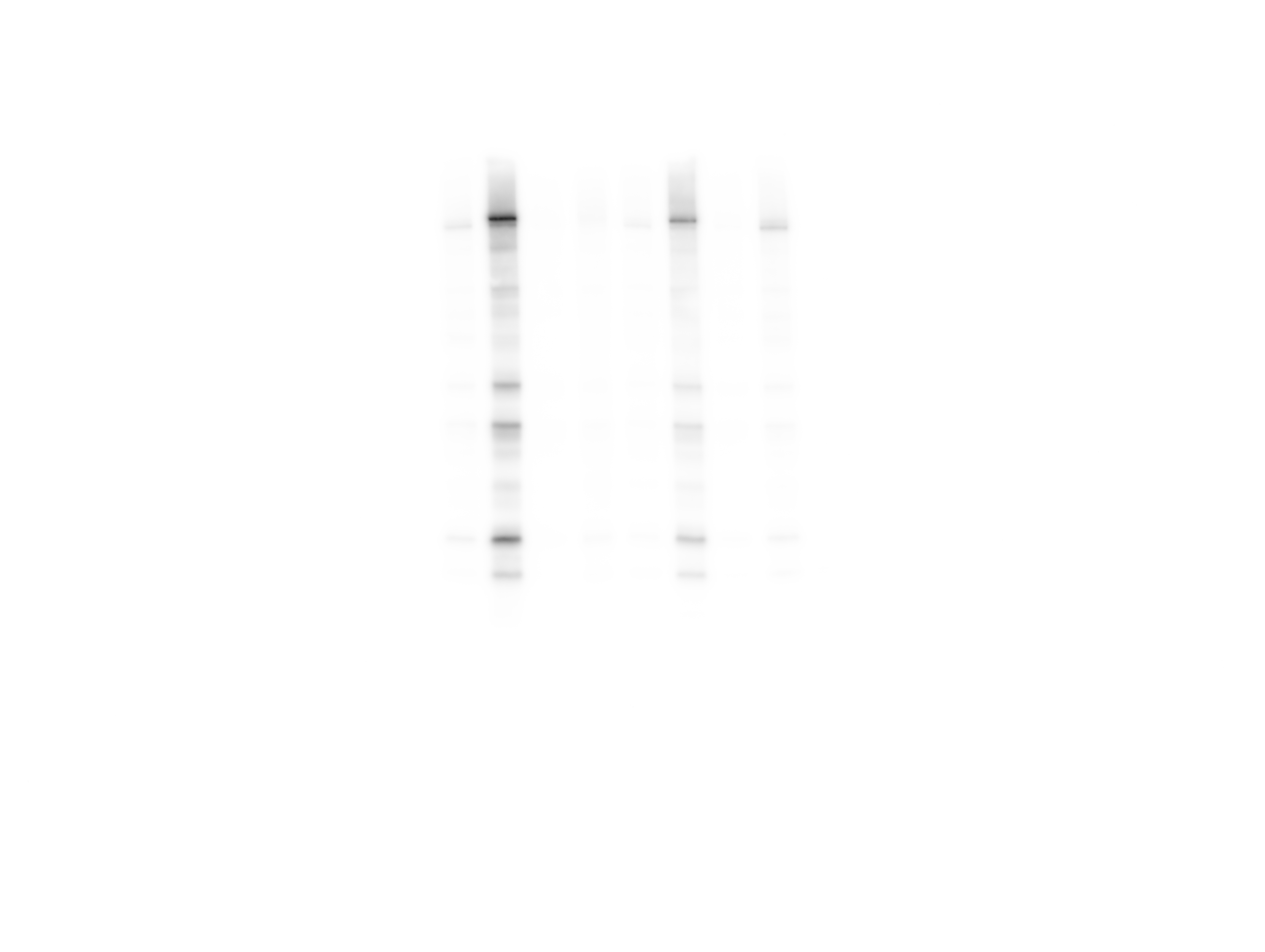

Supplement: Figure 3—source data 2. [file elife-74255-fig3-data2.zip › Figure 3C - source Caf130 TAP IP (2).tif]

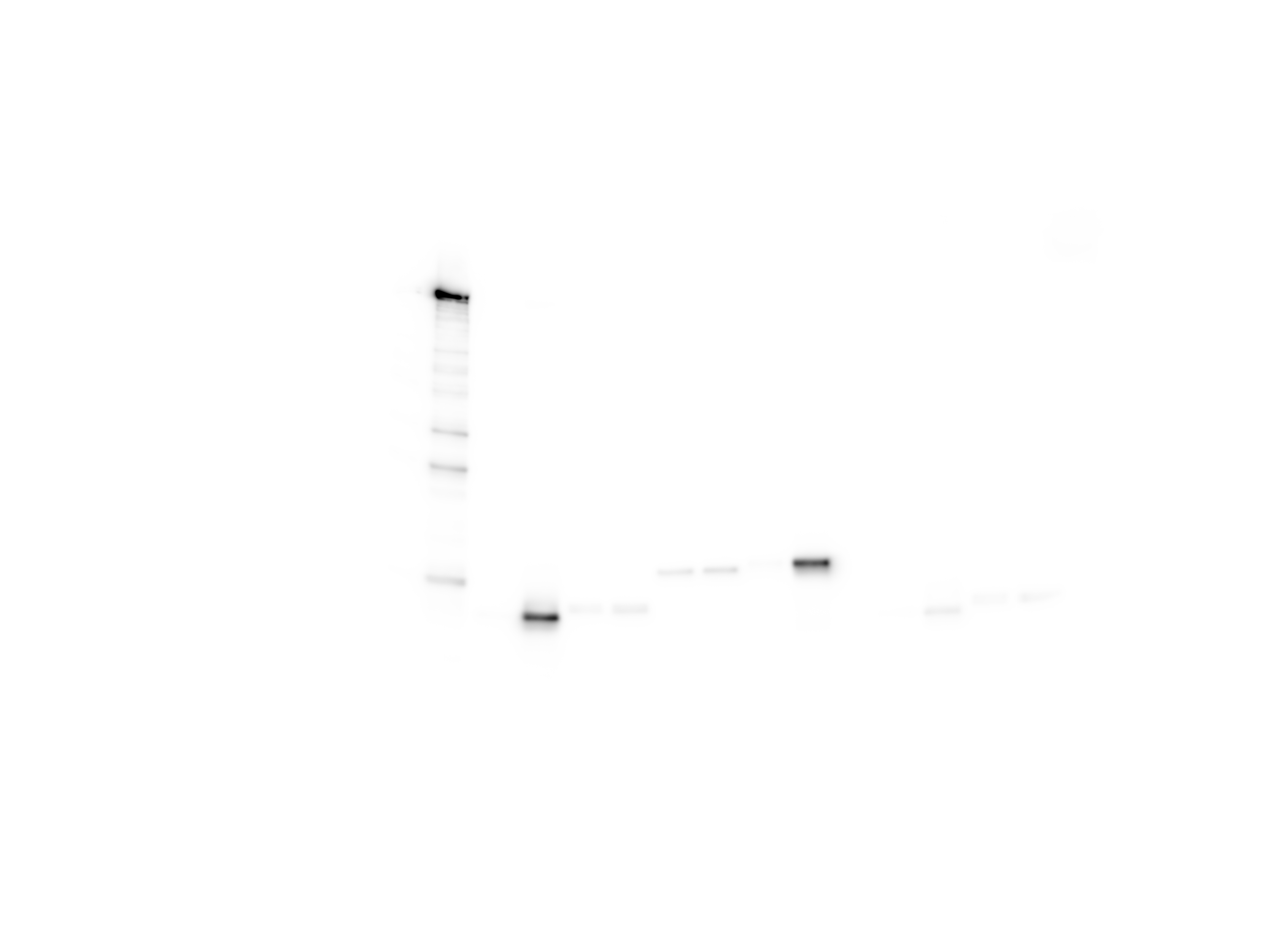

Supplement: Figure 3—source data 2. [file elife-74255-fig3-data2.zip › Figure 3C - source Caf130 TAP IP.tif]

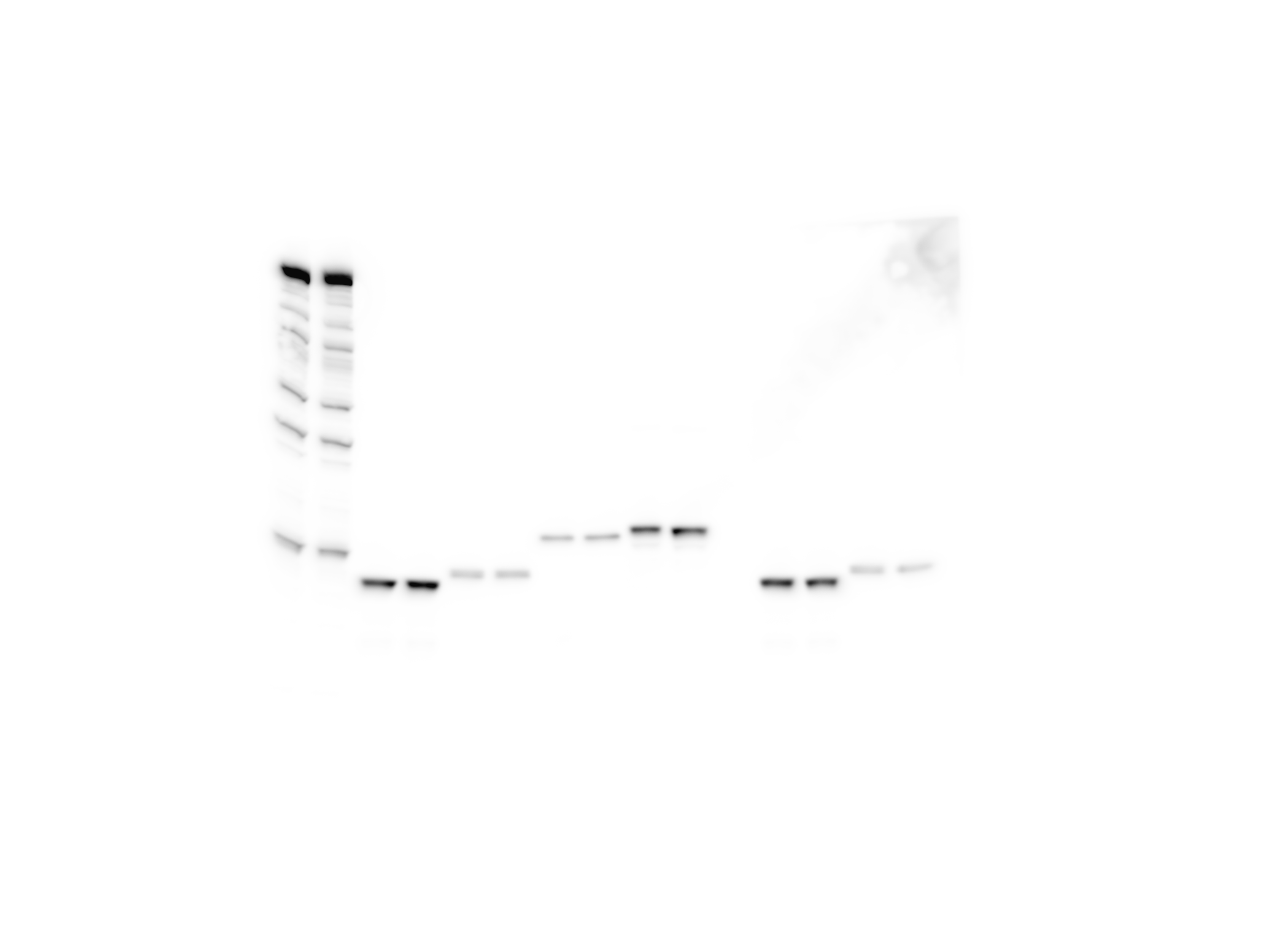

Supplement: Figure 3—source data 2. [file elife-74255-fig3-data2.zip › Figure 3C - source Caf130 TAP total.tif]

# Figure 3C - source data

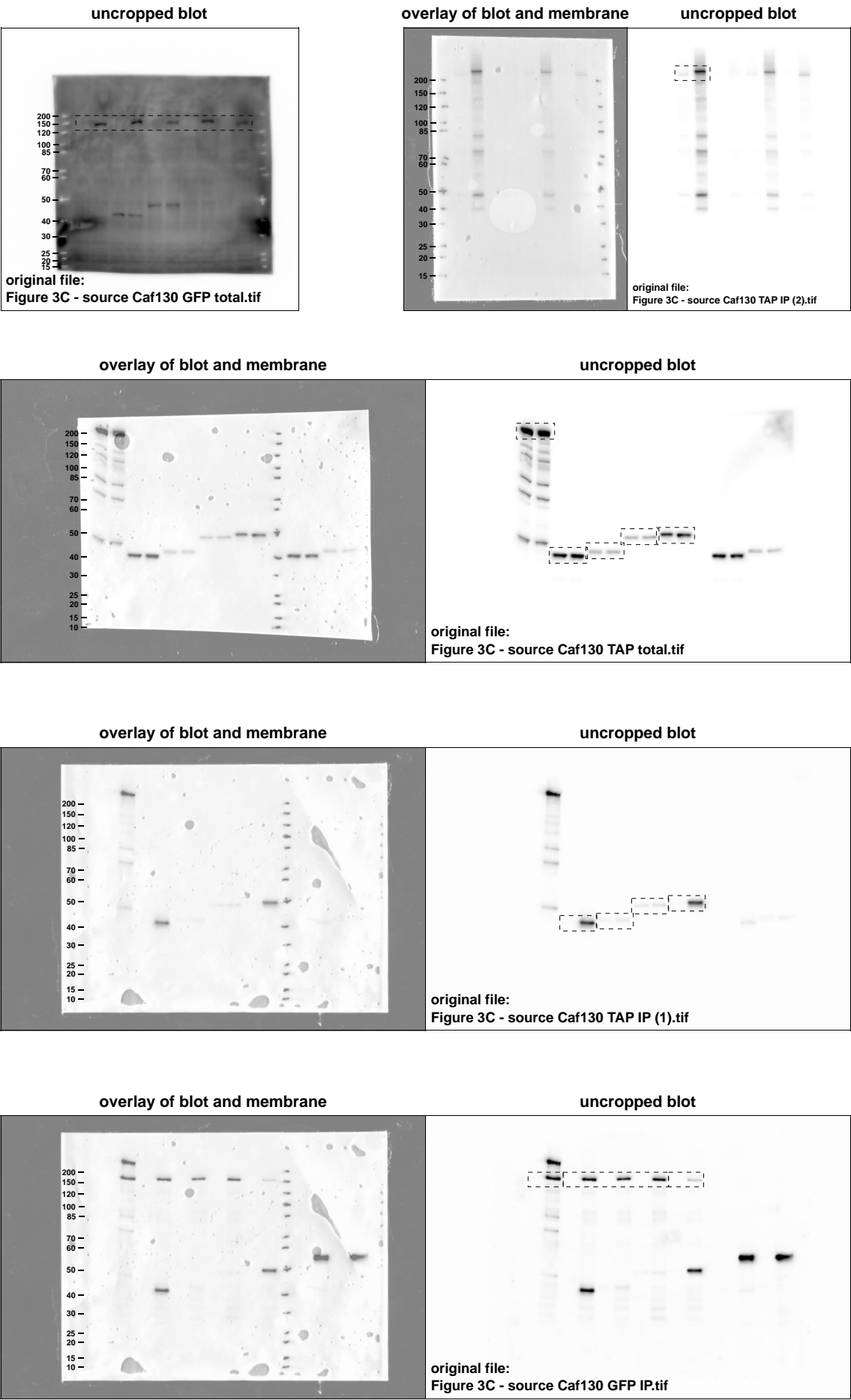

Supplement: Figure 3—source data 2. [file elife-74255-fig3-data2.zip › Figure 3C - source data.pdf]

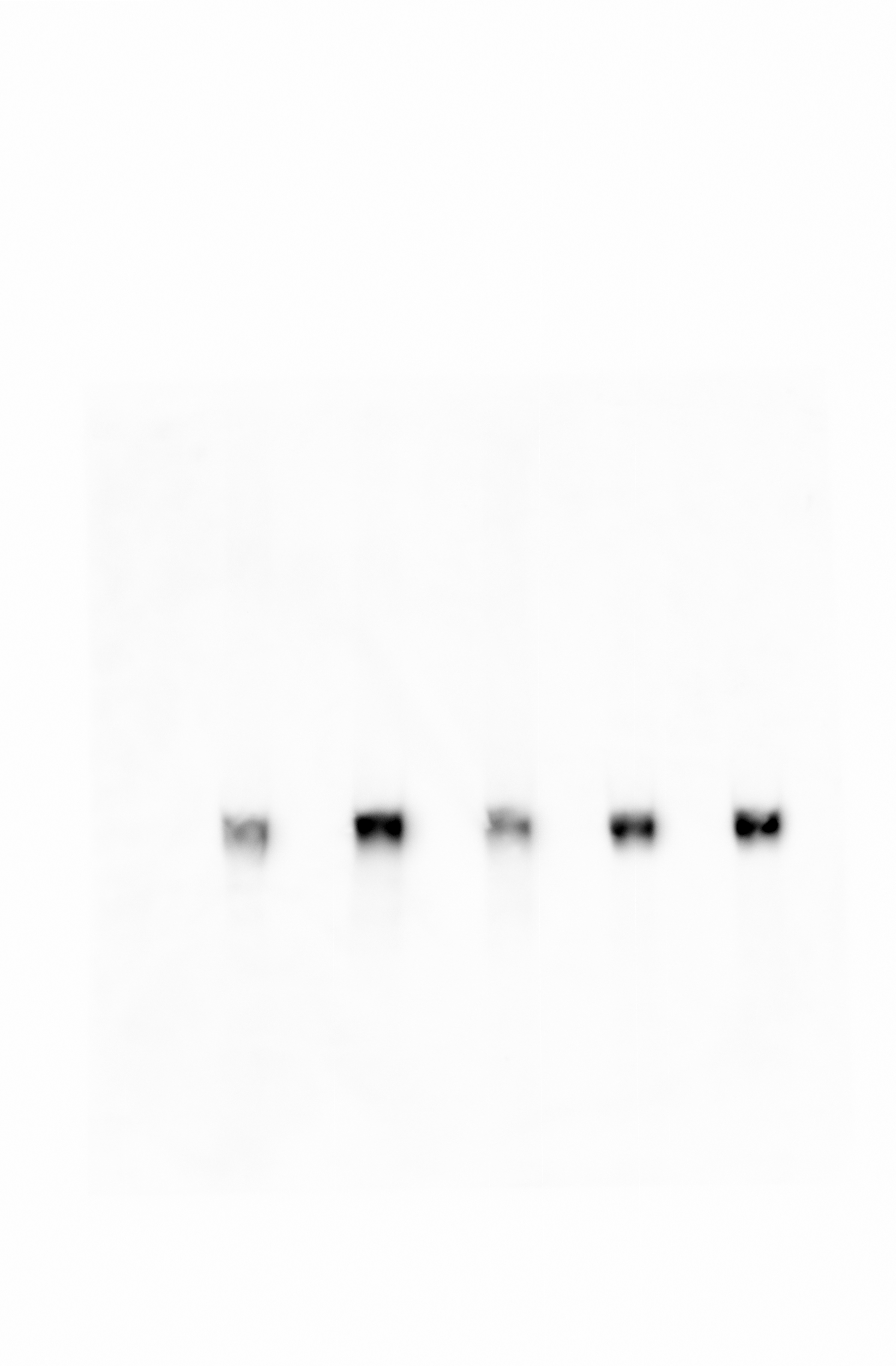

Supplement: Figure 3—source data 3. [file elife-74255-fig3-data3.zip › Figure 3D - source Cal4 GFP IP.tif]

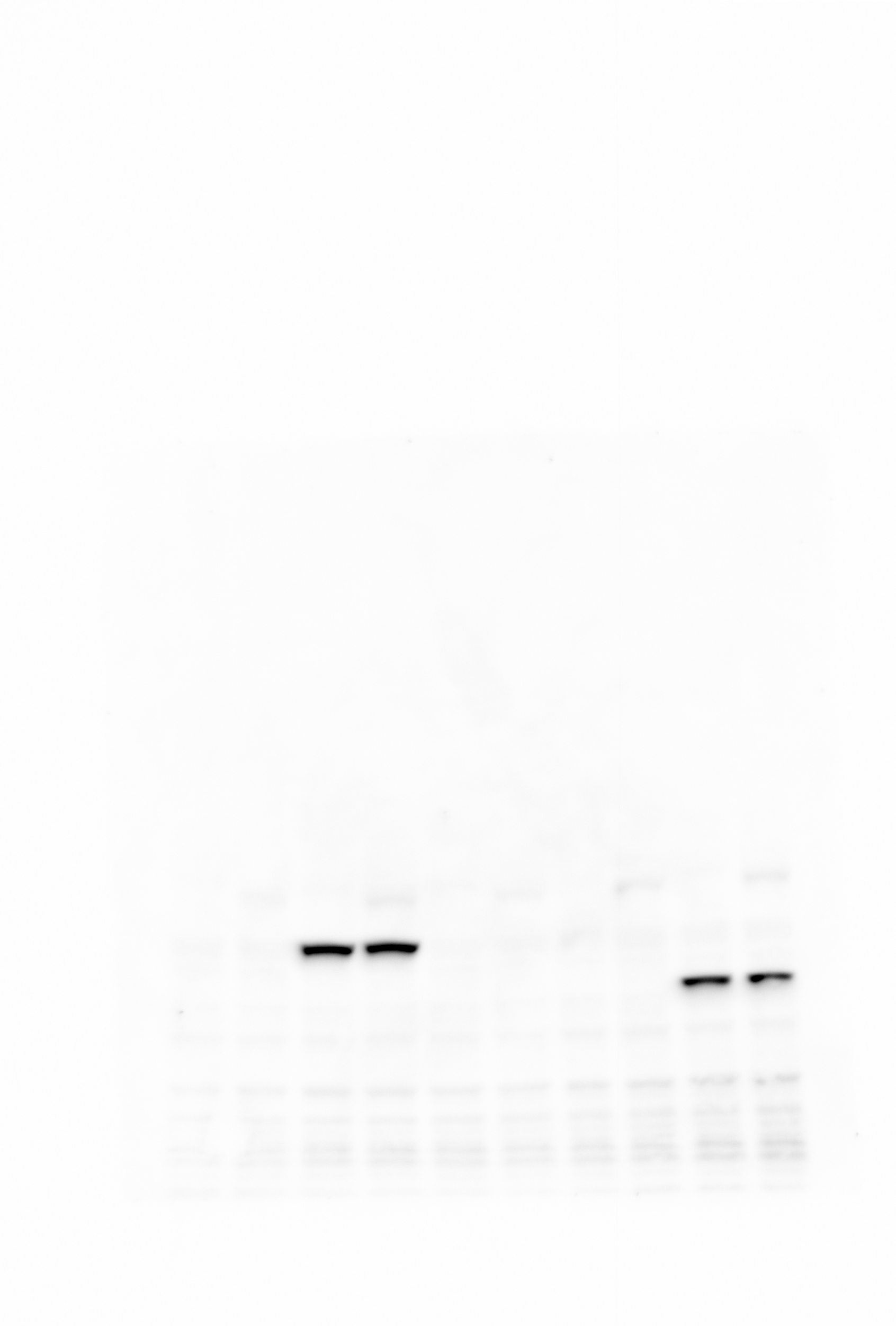

Supplement: Figure 3—source data 3. [file elife-74255-fig3-data3.zip › Figure 3D - source Cal4 GFP total.tif]

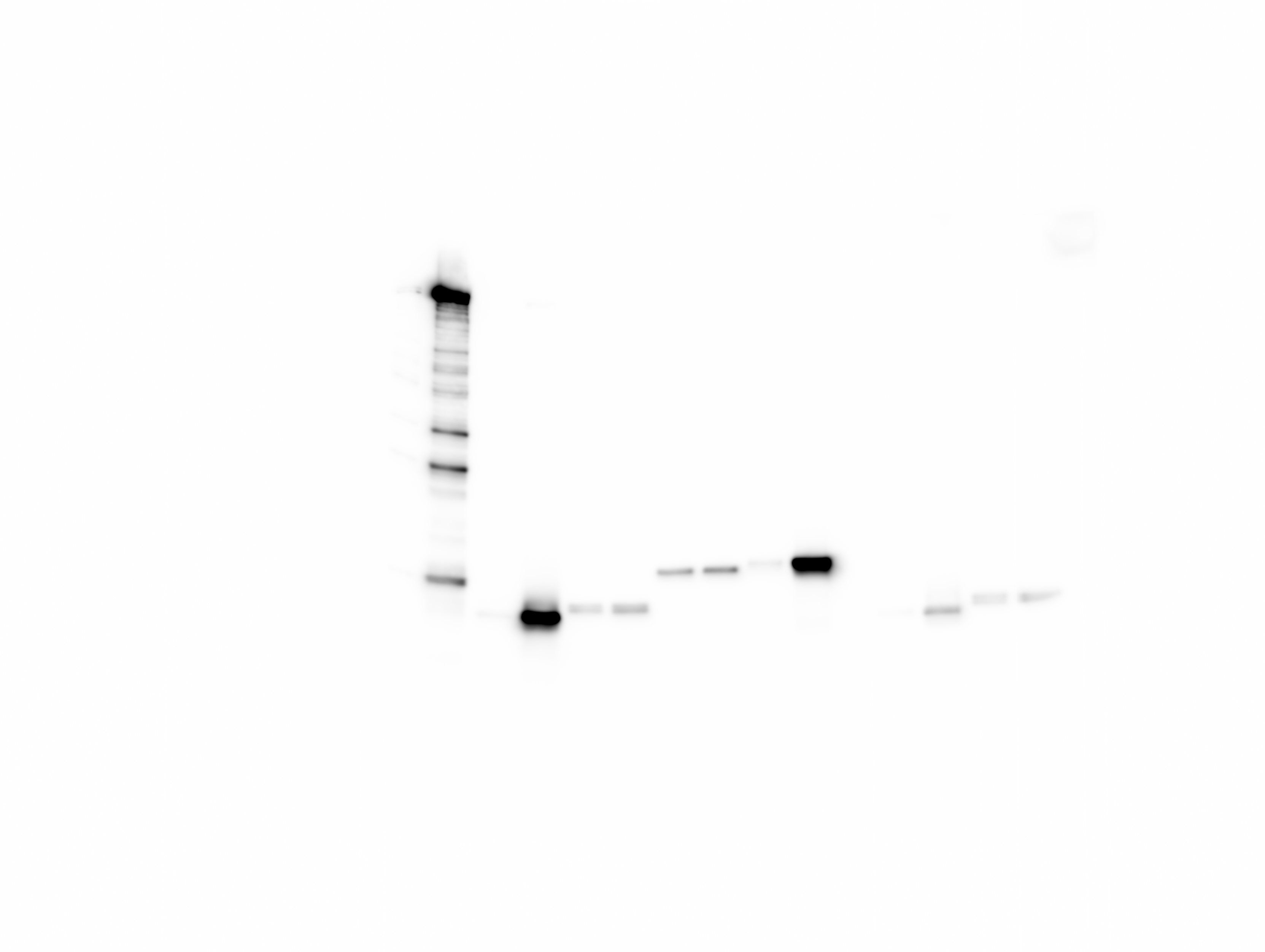

Supplement: Figure 3—source data 3. [file elife-74255-fig3-data3.zip › Figure 3D - source Cal4 TAP IP.tif]

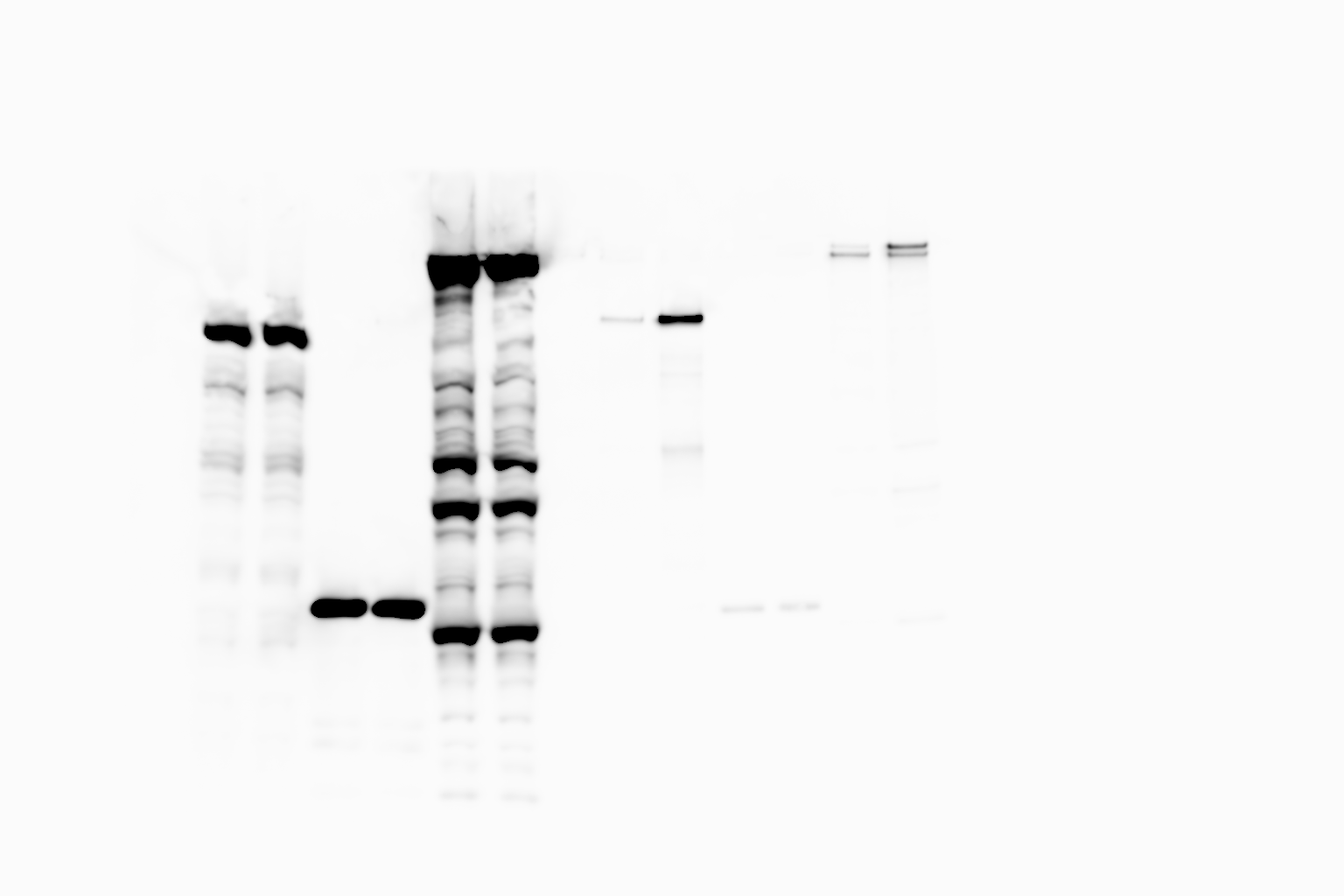

Supplement: Figure 3—source data 3. [file elife-74255-fig3-data3.zip › Figure 3D - source Cal4 TAP total and IP (1).tif]

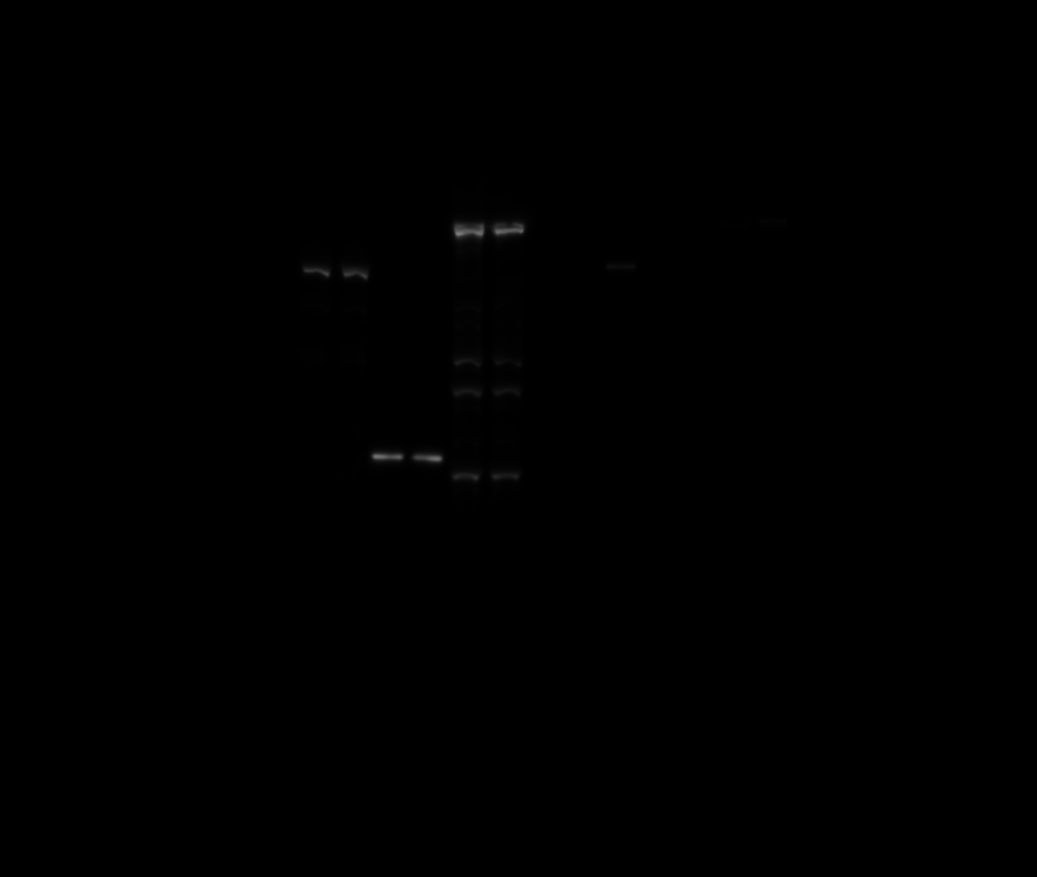

Supplement: Figure 3—source data 3. [file elife-74255-fig3-data3.zip › Figure 3D - source Cal4 TAP total and IP (2).tif]

# Figure 3D - source data

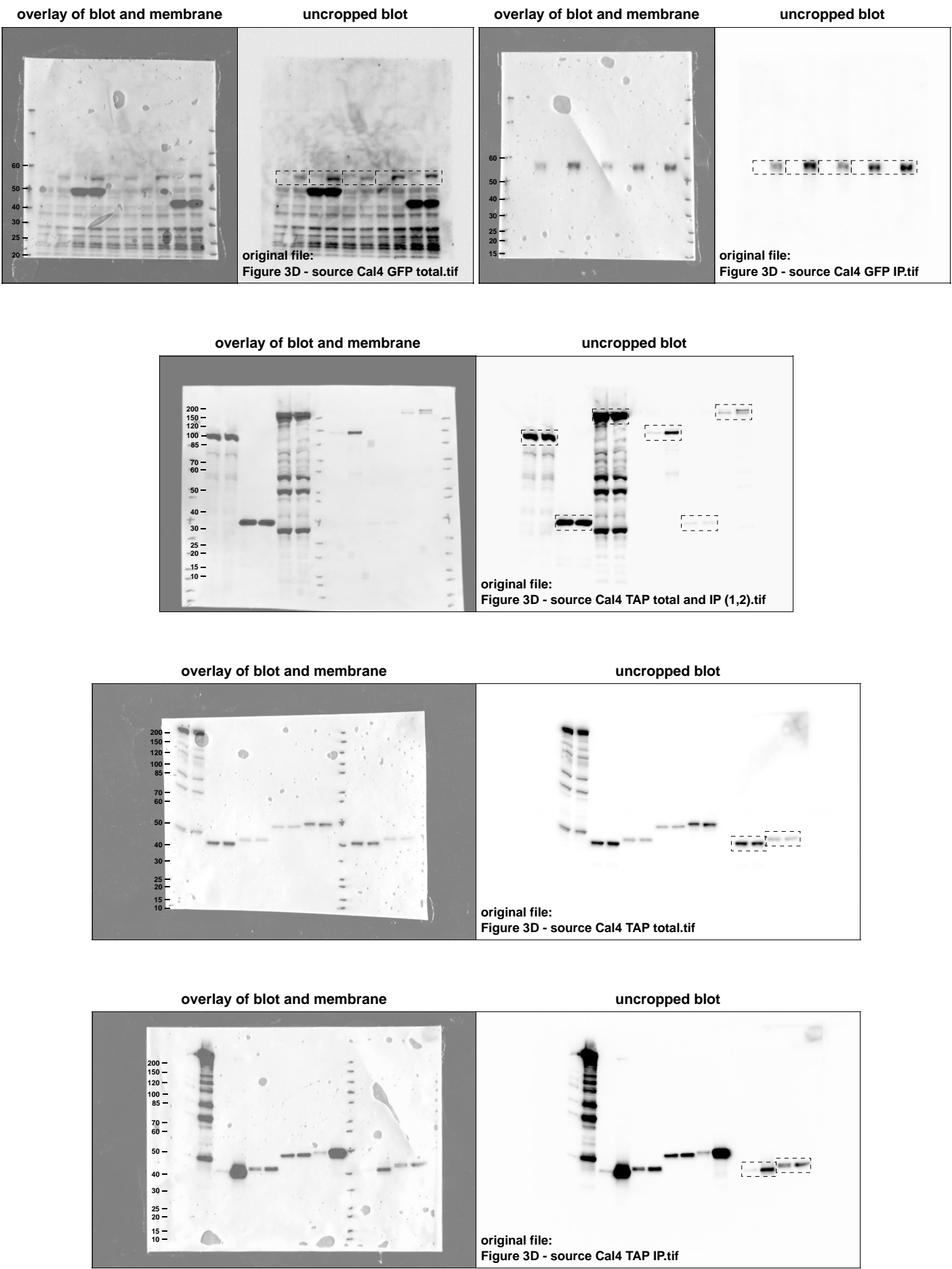

Supplement: Figure 3—source data 3. [file elife-74255-fig3-data3.zip › Figure 3D - source data.pdf]

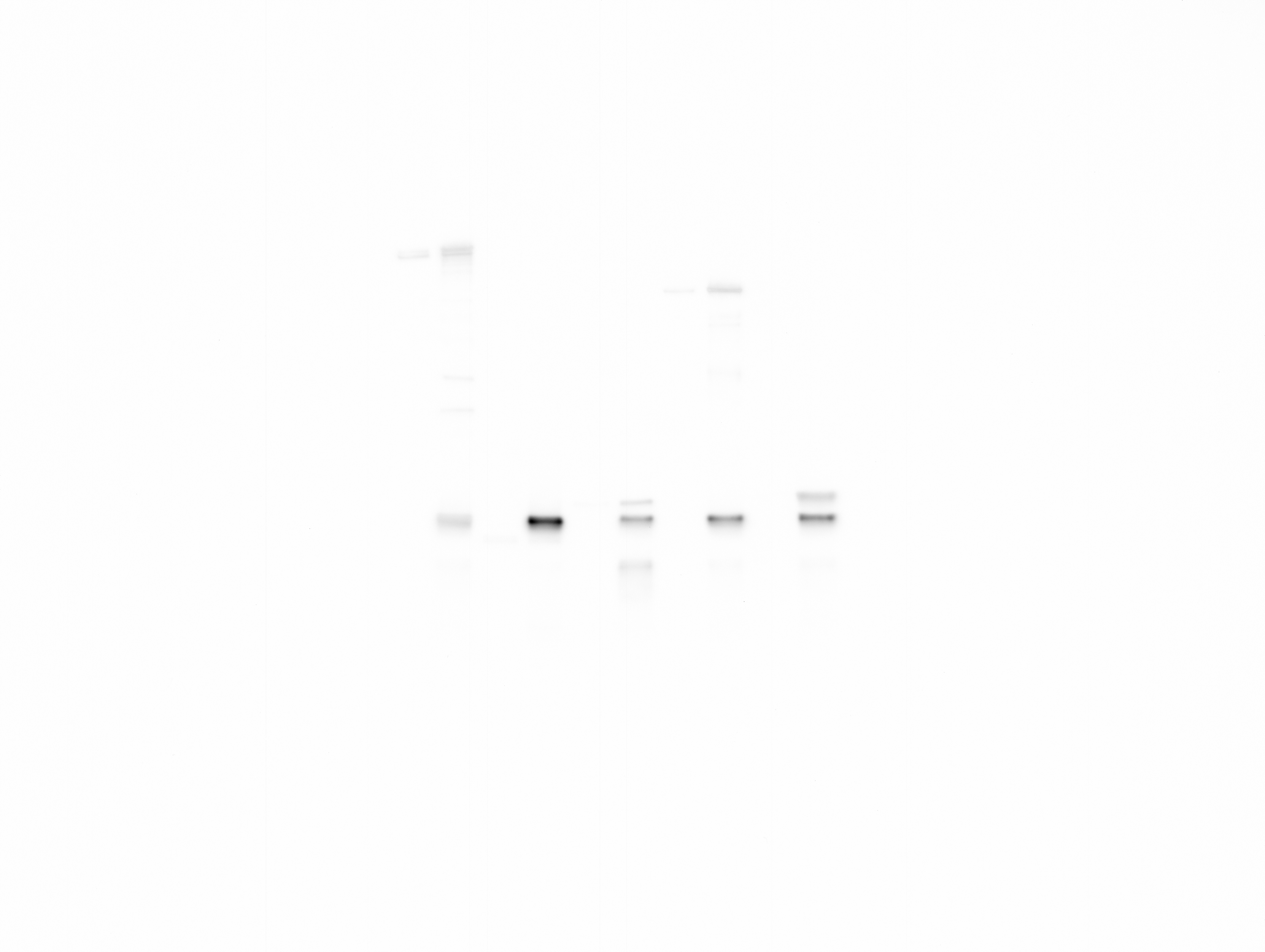

Supplement: Figure 3—source data 4. [file elife-74255-fig3-data4.zip › Figure 3E - source Btt1 GFP IP.tif]

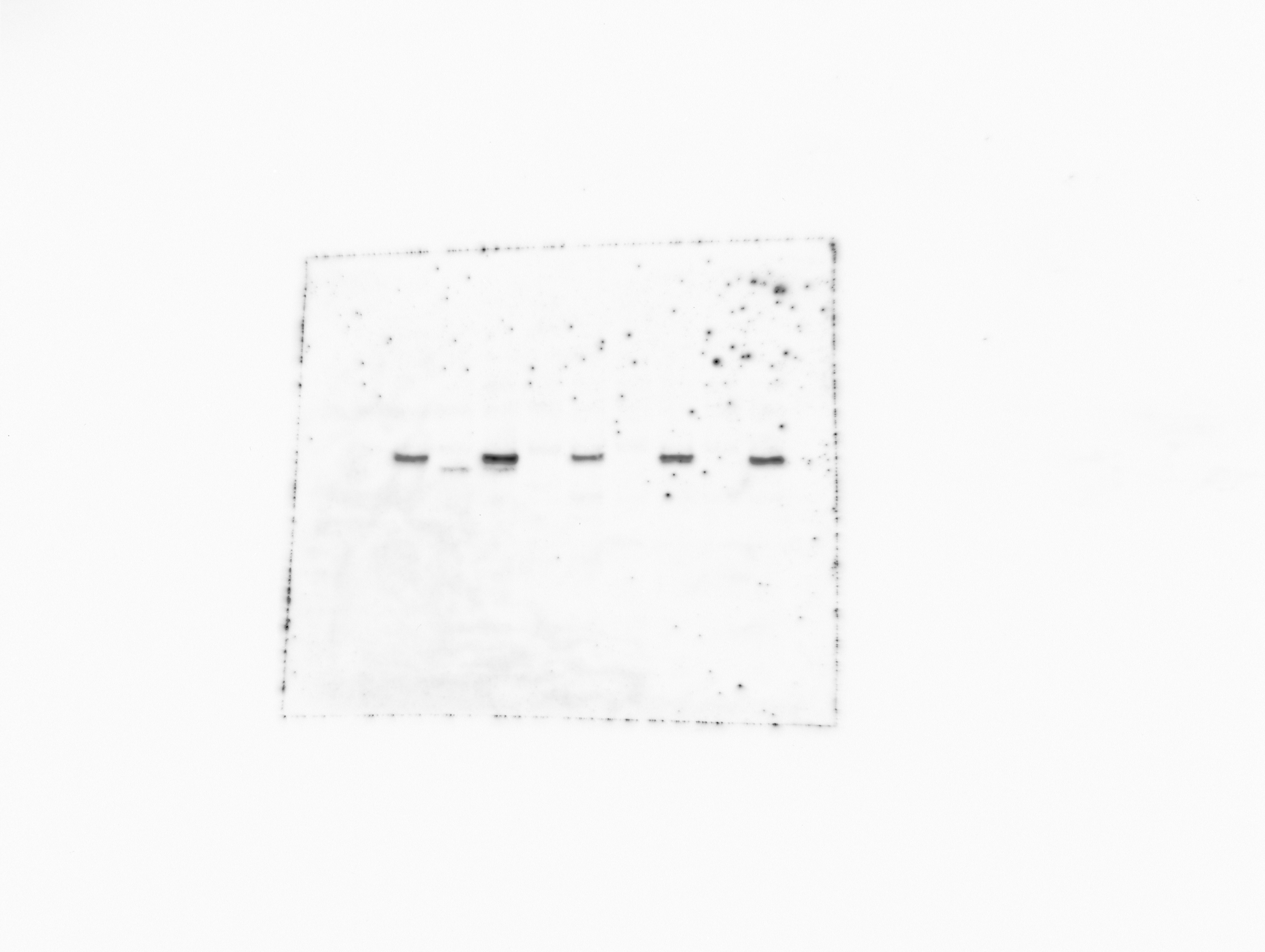

Supplement: Figure 3—source data 4. [file elife-74255-fig3-data4.zip › Figure 3E - source Btt1 GFP total.tif]

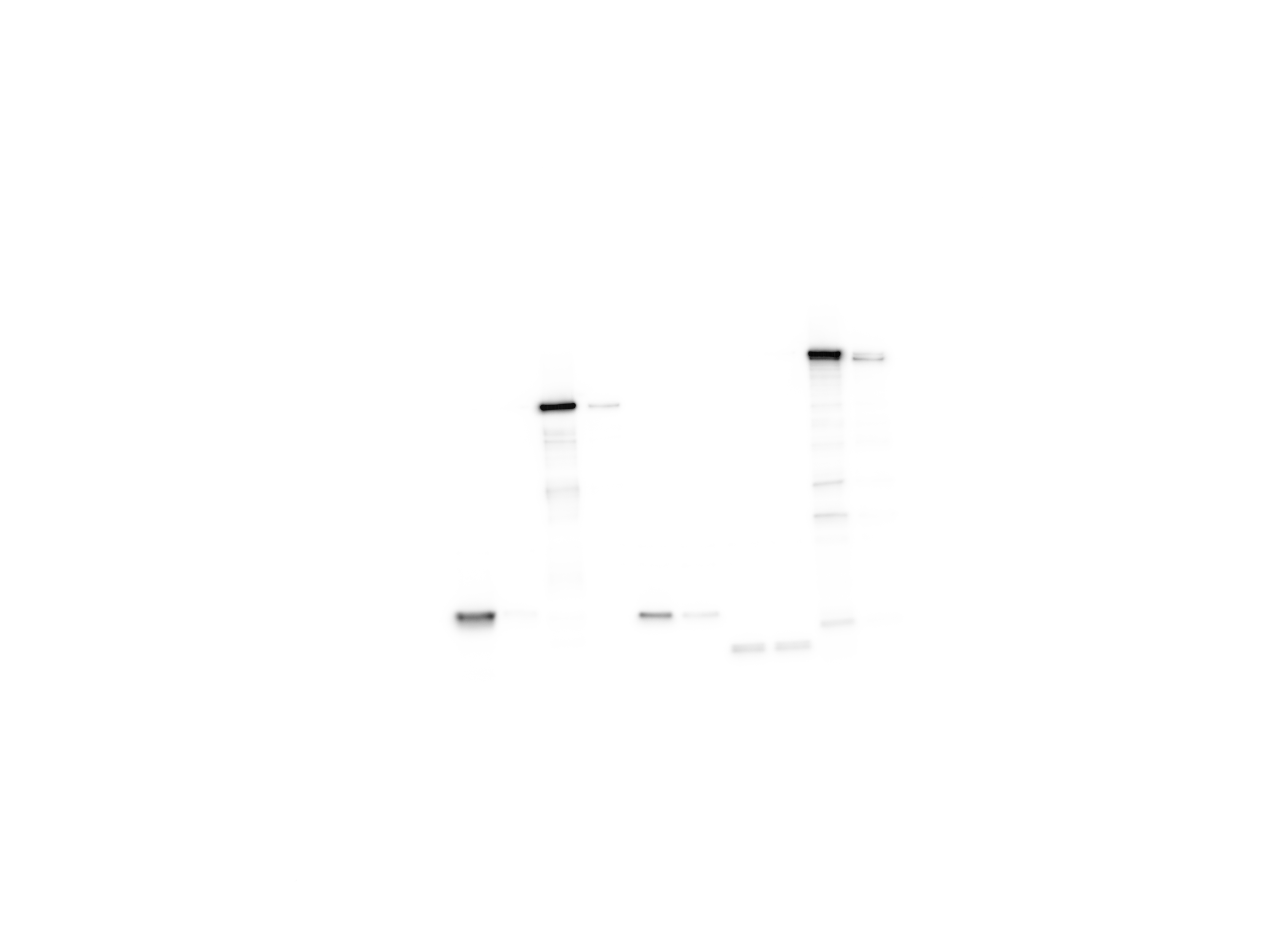

Supplement: Figure 3—source data 4. [file elife-74255-fig3-data4.zip › Figure 3E - source Btt1 TAP IP (1).tif]

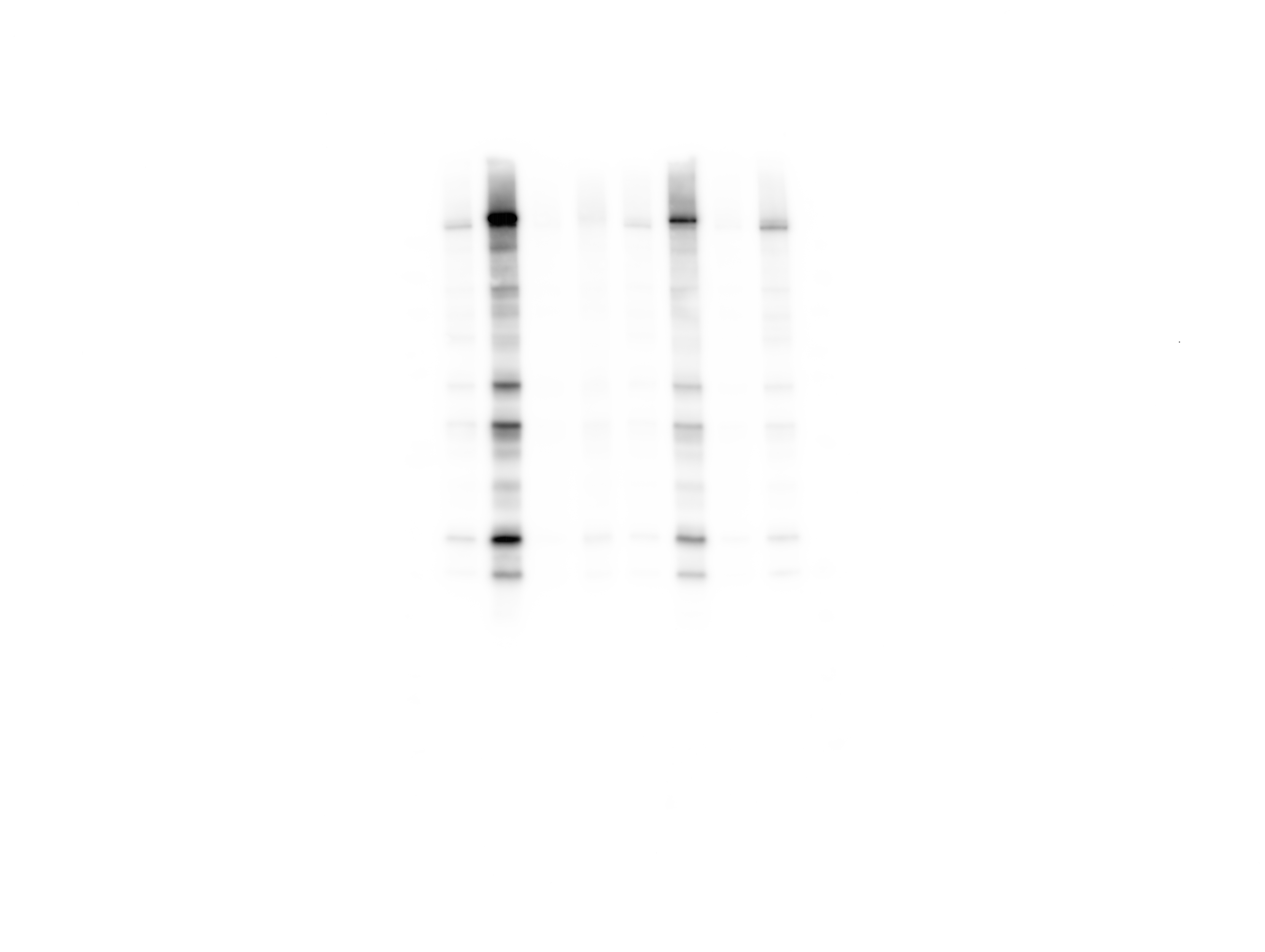

Supplement: Figure 3—source data 4. [file elife-74255-fig3-data4.zip › Figure 3E - source Btt1 TAP IP (2).tif]

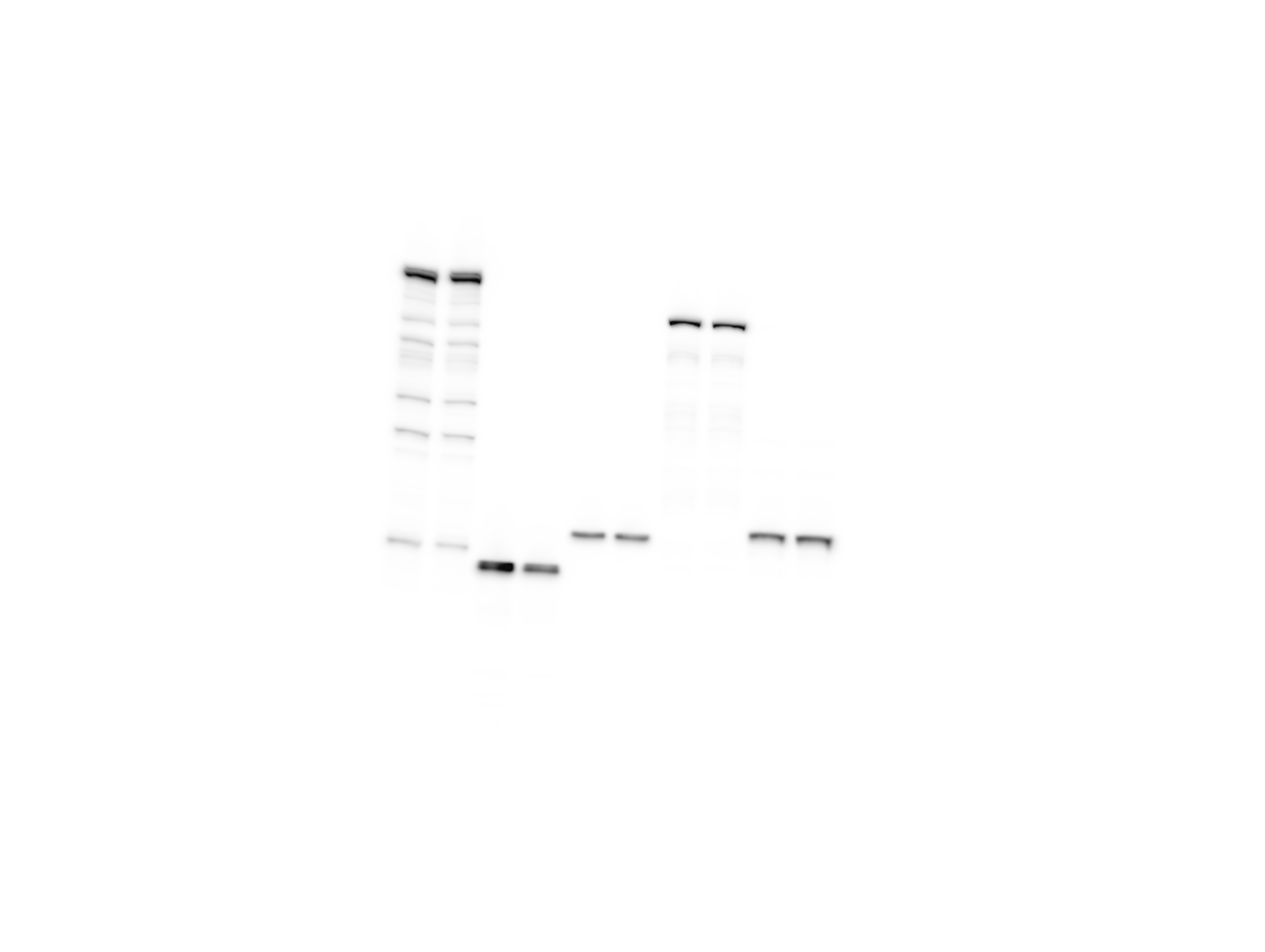

Supplement: Figure 3—source data 4. [file elife-74255-fig3-data4.zip › Figure 3E - source Btt1 TAP total.tif]

# Figure 3E - source data

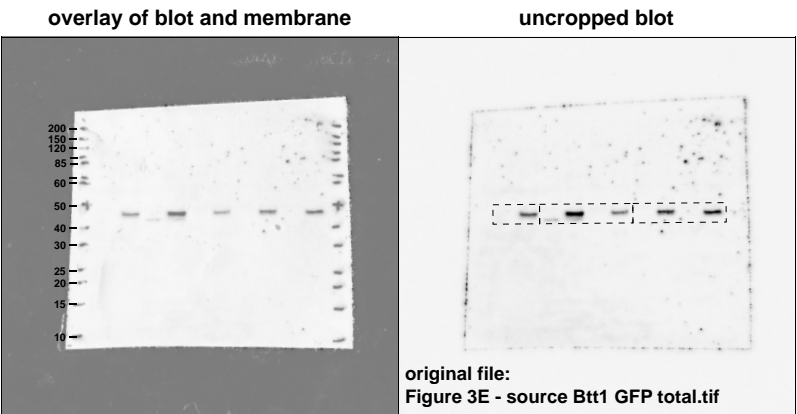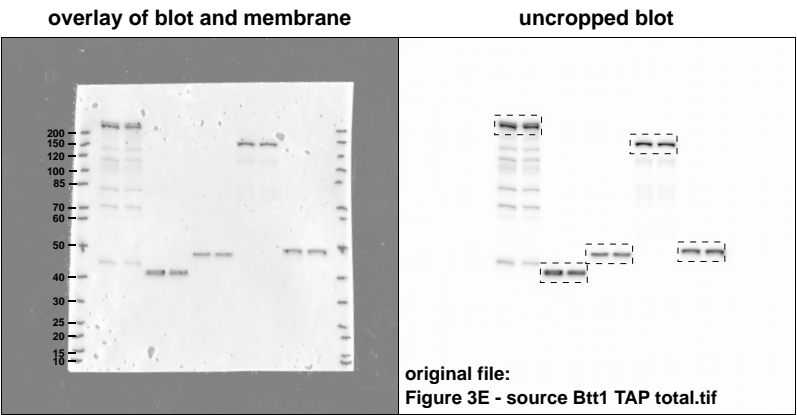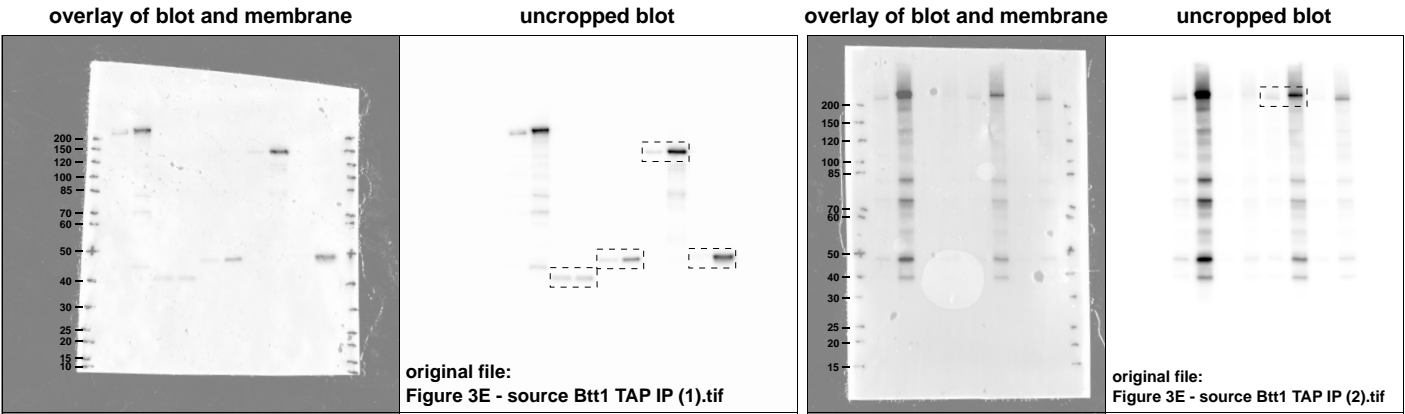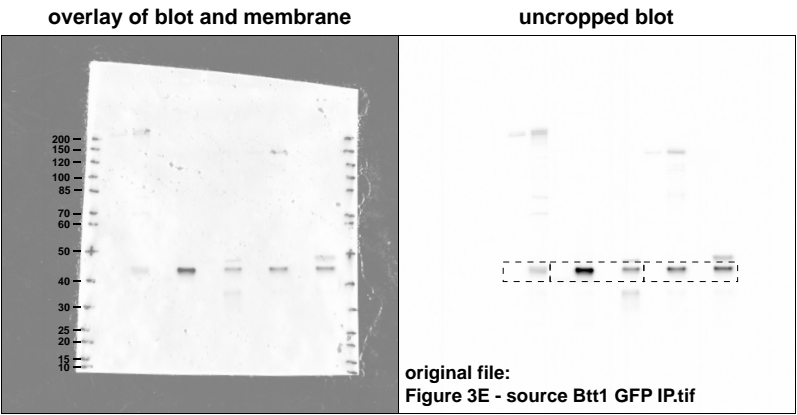

Supplement: Figure 3—source data 4. [file elife-74255-fig3-data4.zip › Figure 3E - source data.pdf]

# Figure 3F - source data

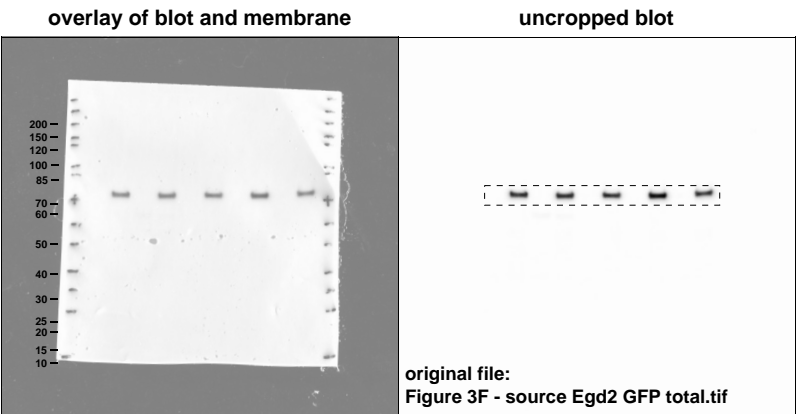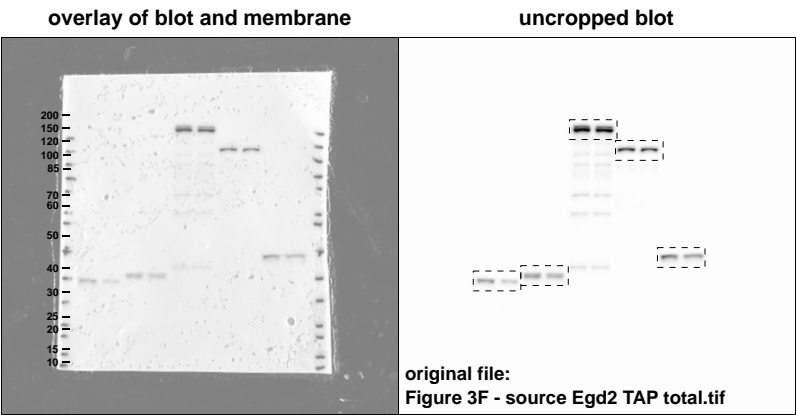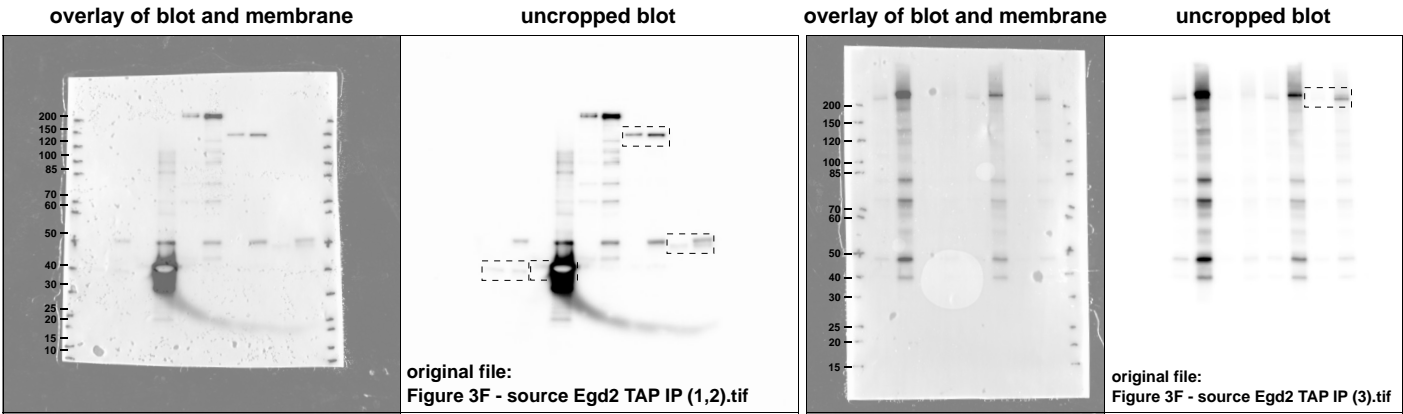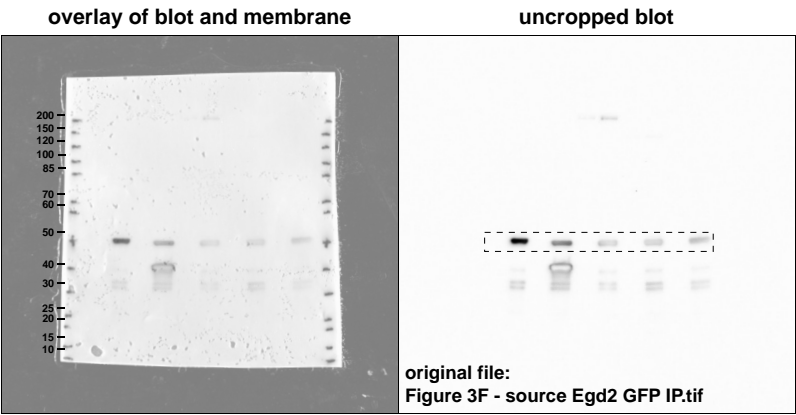

Supplement: Figure 3—source data 5. [file elife-74255-fig3-data5.zip › Figure 3F - source data.pdf]

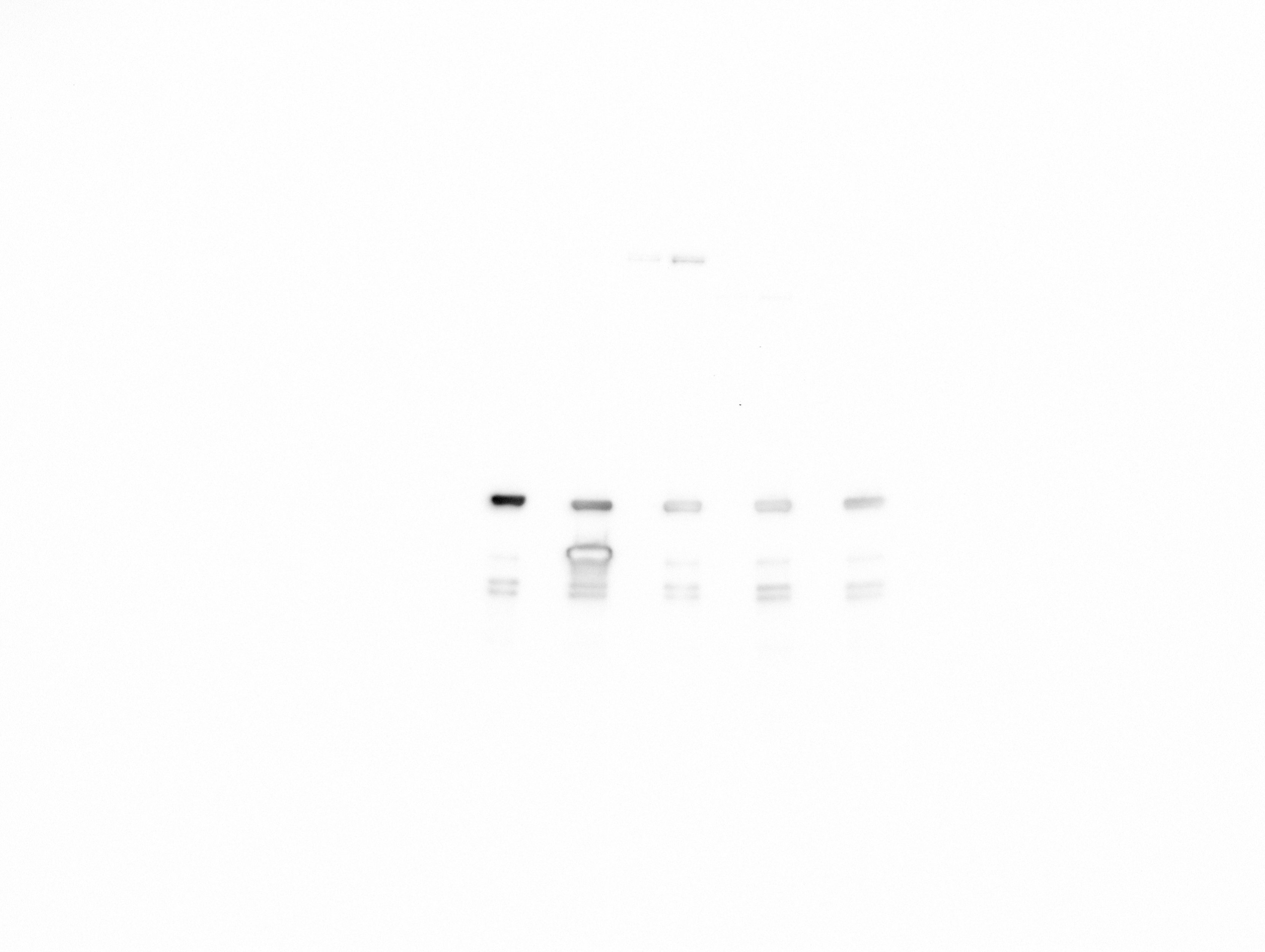

Supplement: Figure 3—source data 5. [file elife-74255-fig3-data5.zip › Figure 3F - source Egd2 GFP IP.tif]

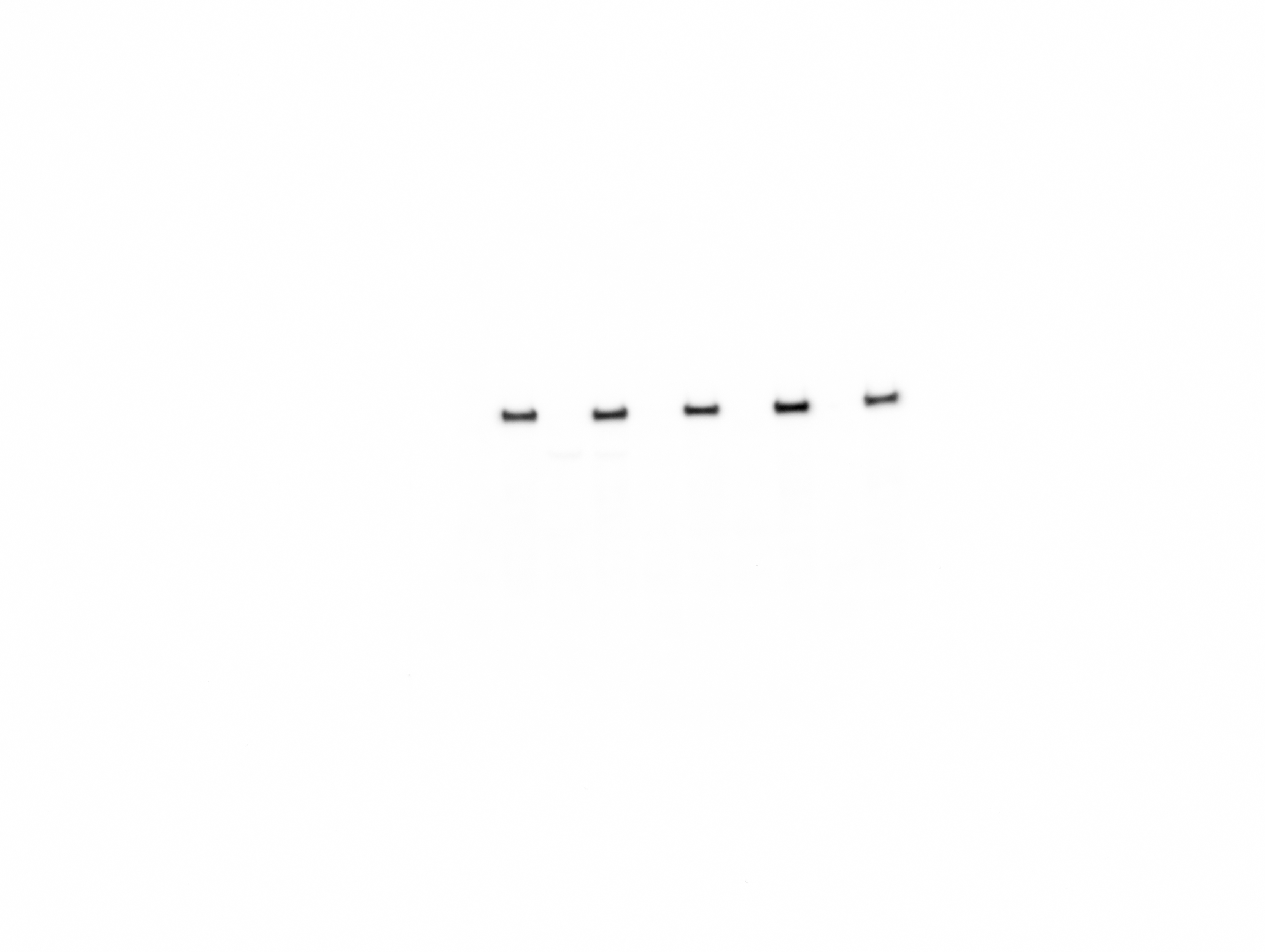

Supplement: Figure 3—source data 5. [file elife-74255-fig3-data5.zip › Figure 3F - source Egd2 GFP total.tif]

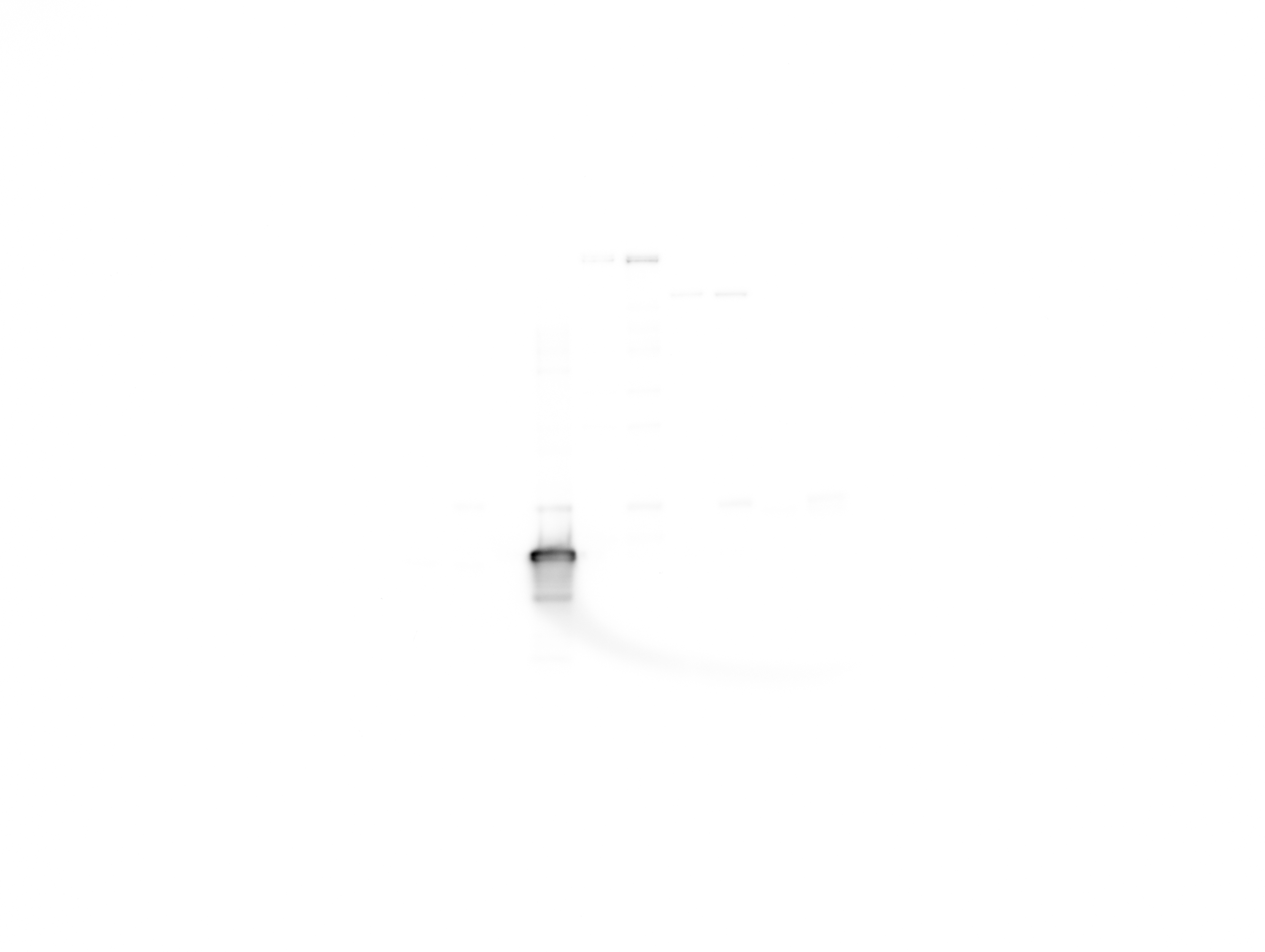

Supplement: Figure 3—source data 5. [file elife-74255-fig3-data5.zip › Figure 3F - source Egd2 TAP IP (1).tif]

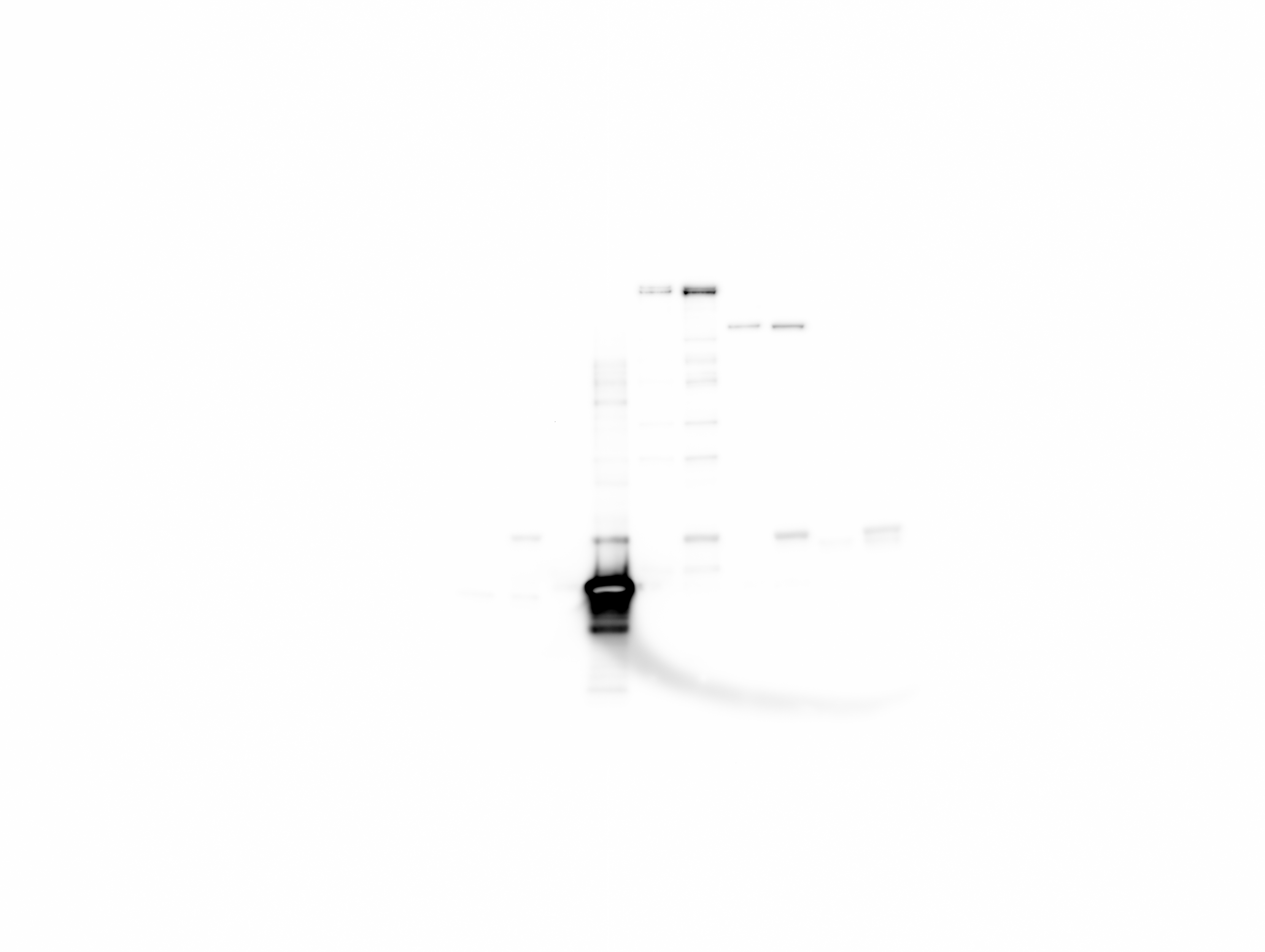

Supplement: Figure 3—source data 5. [file elife-74255-fig3-data5.zip › Figure 3F - source Egd2 TAP IP (2).tif]

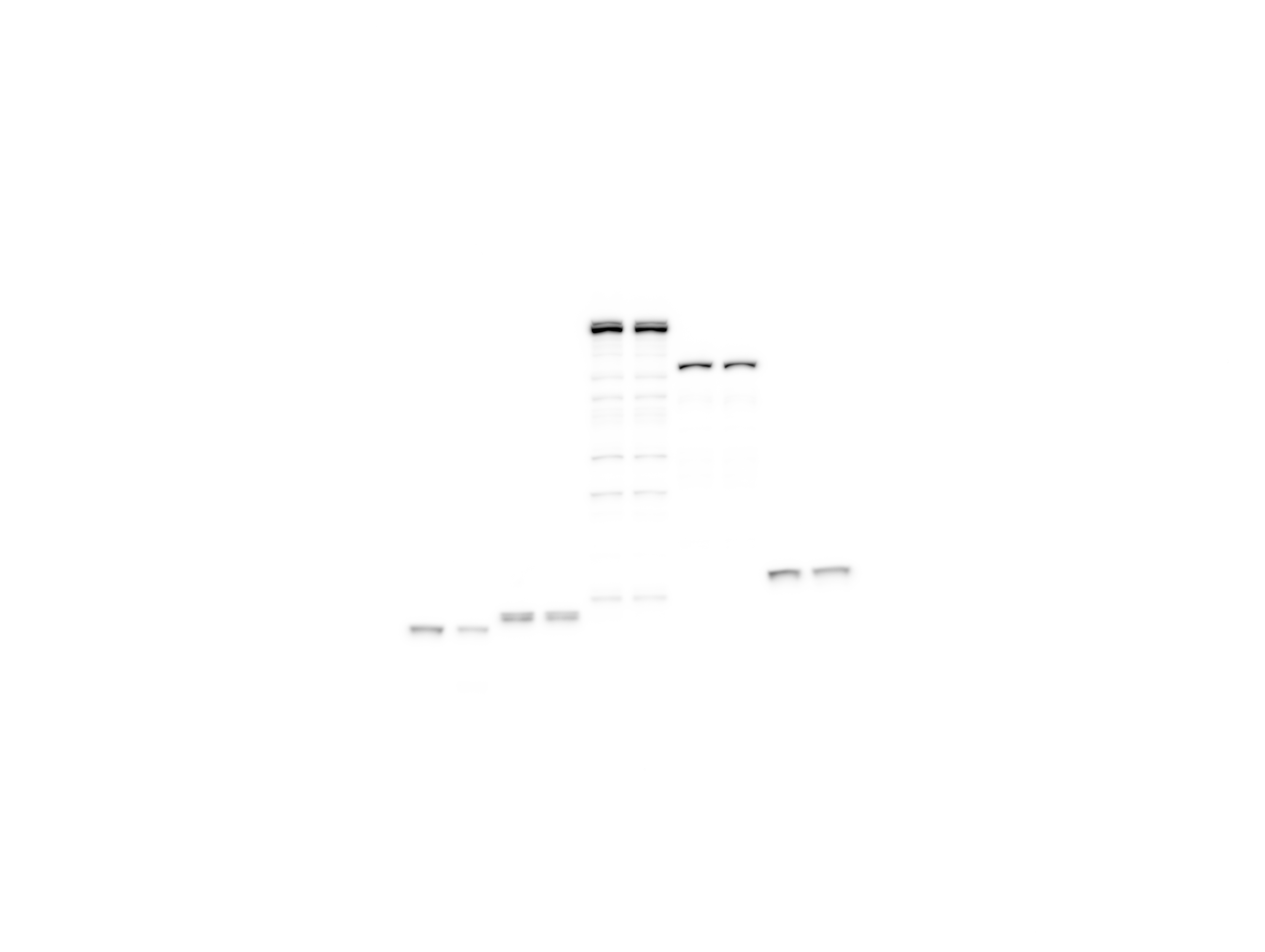

Supplement: Figure 3—source data 5. [file elife-74255-fig3-data5.zip › Figure 3F - source Egd2 TAP total.tif]

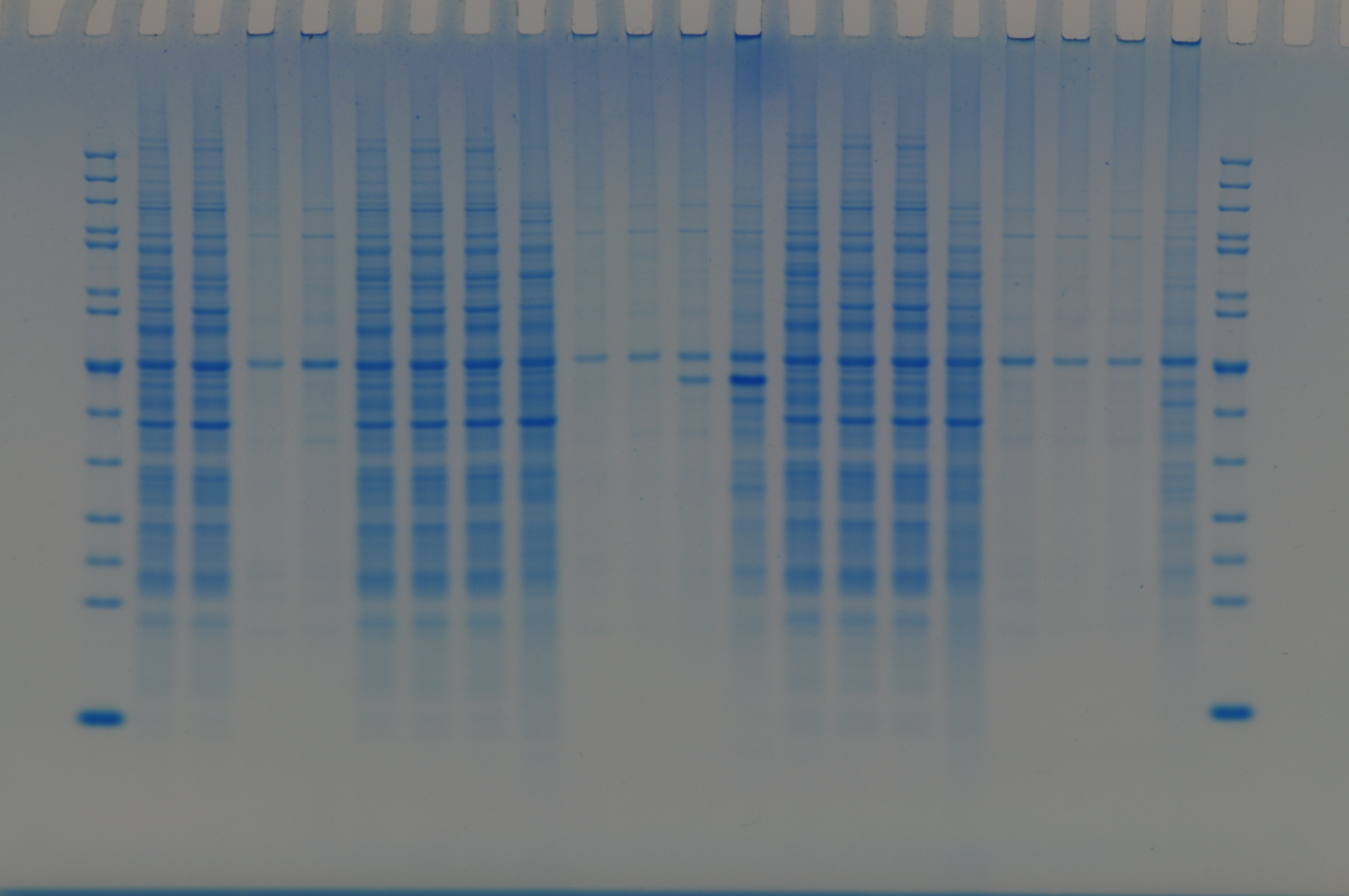

Supplement: Figure 7—source data 1. [file elife-74255-fig7-data1.zip › Figure 7D - source coomassie.JPG]

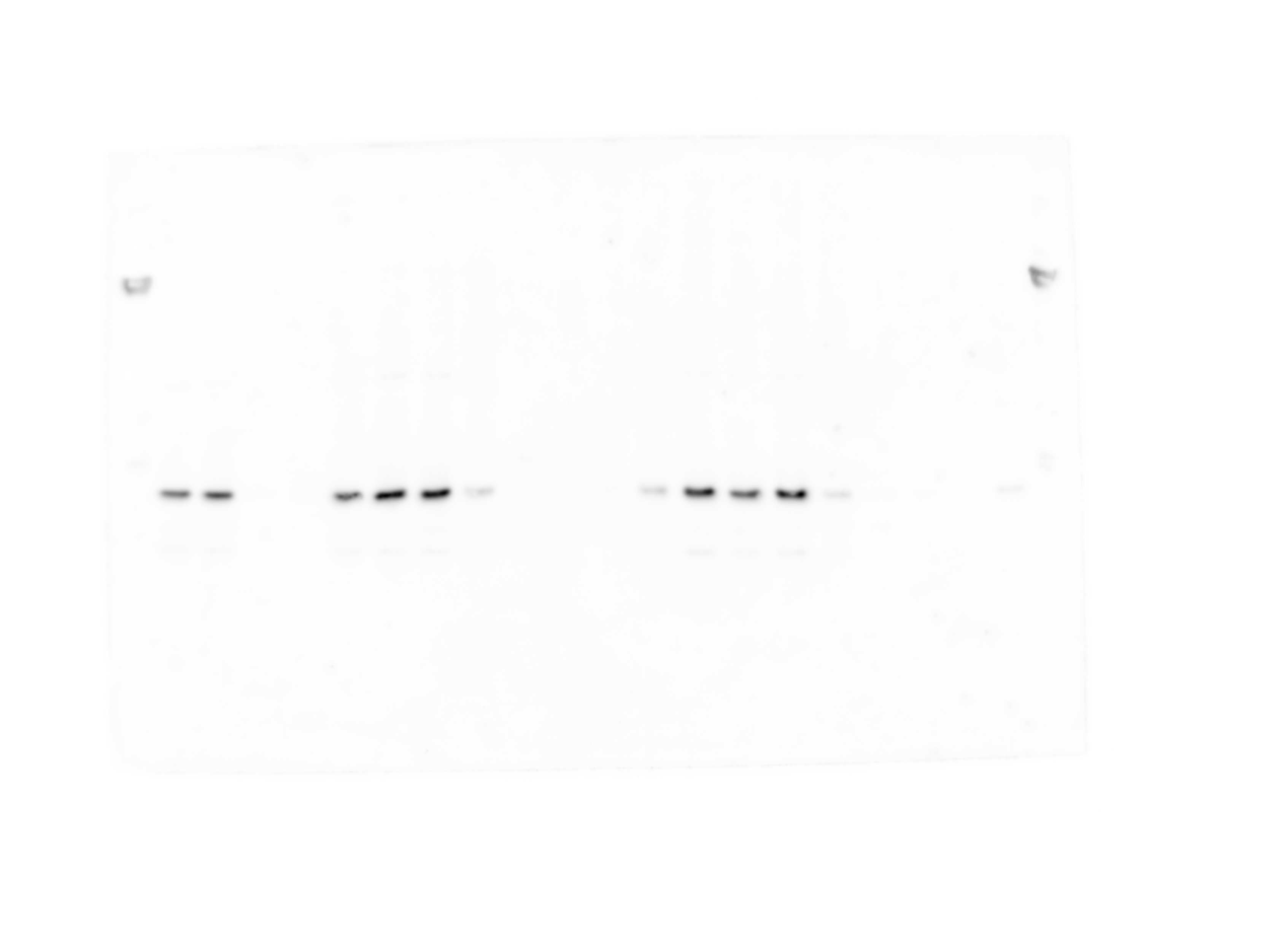

Supplement: Figure 7—source data 1. [file elife-74255-fig7-data1.zip › Figure 7D - source Rpl1.tif]

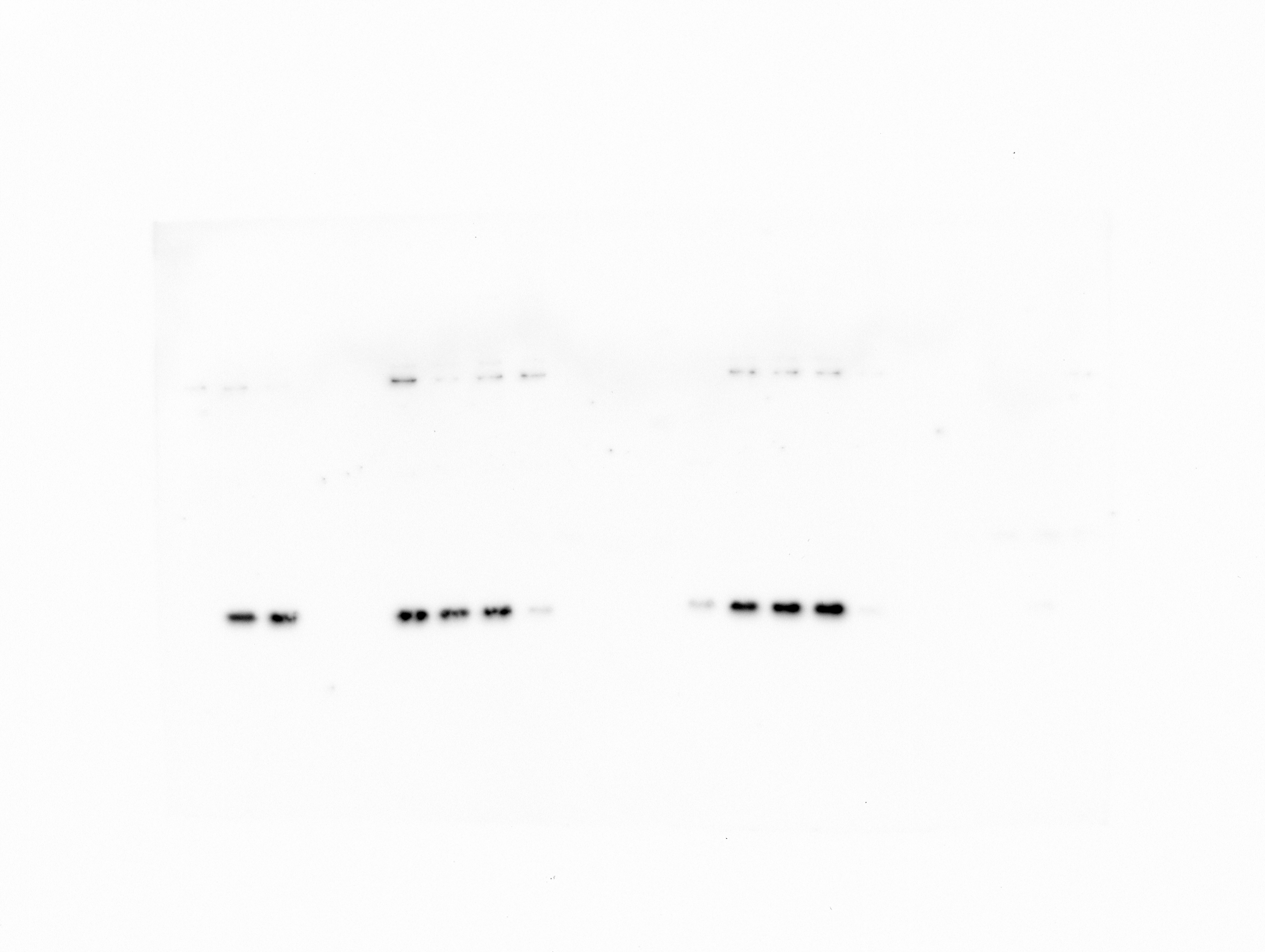

Supplement: Figure 7—source data 1. [file elife-74255-fig7-data1.zip › Figure 7D - source Rpl11.tif]

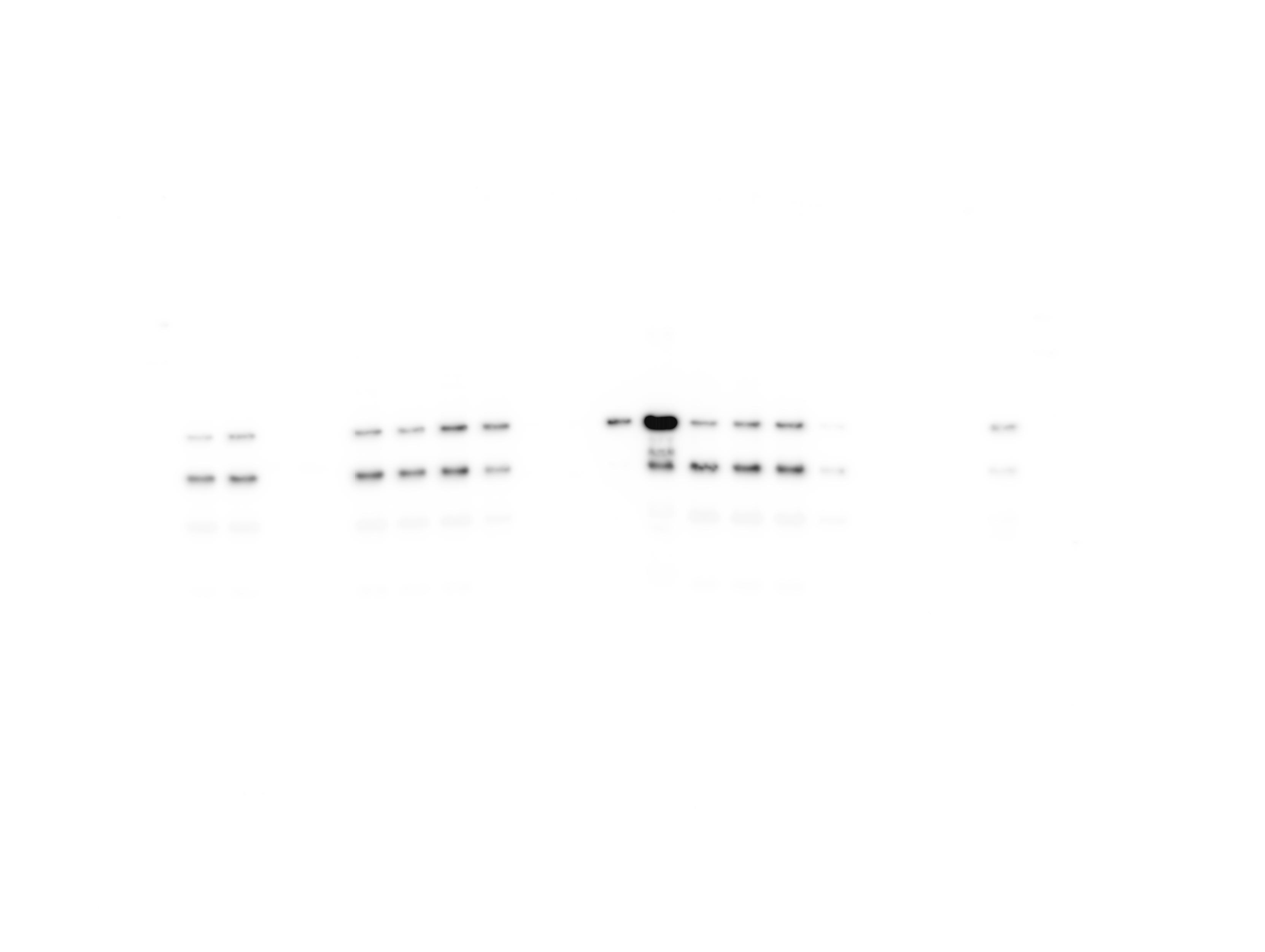

Supplement: Figure 7—source data 1. [file elife-74255-fig7-data1.zip › Figure 7D - source Rpl3.tif]

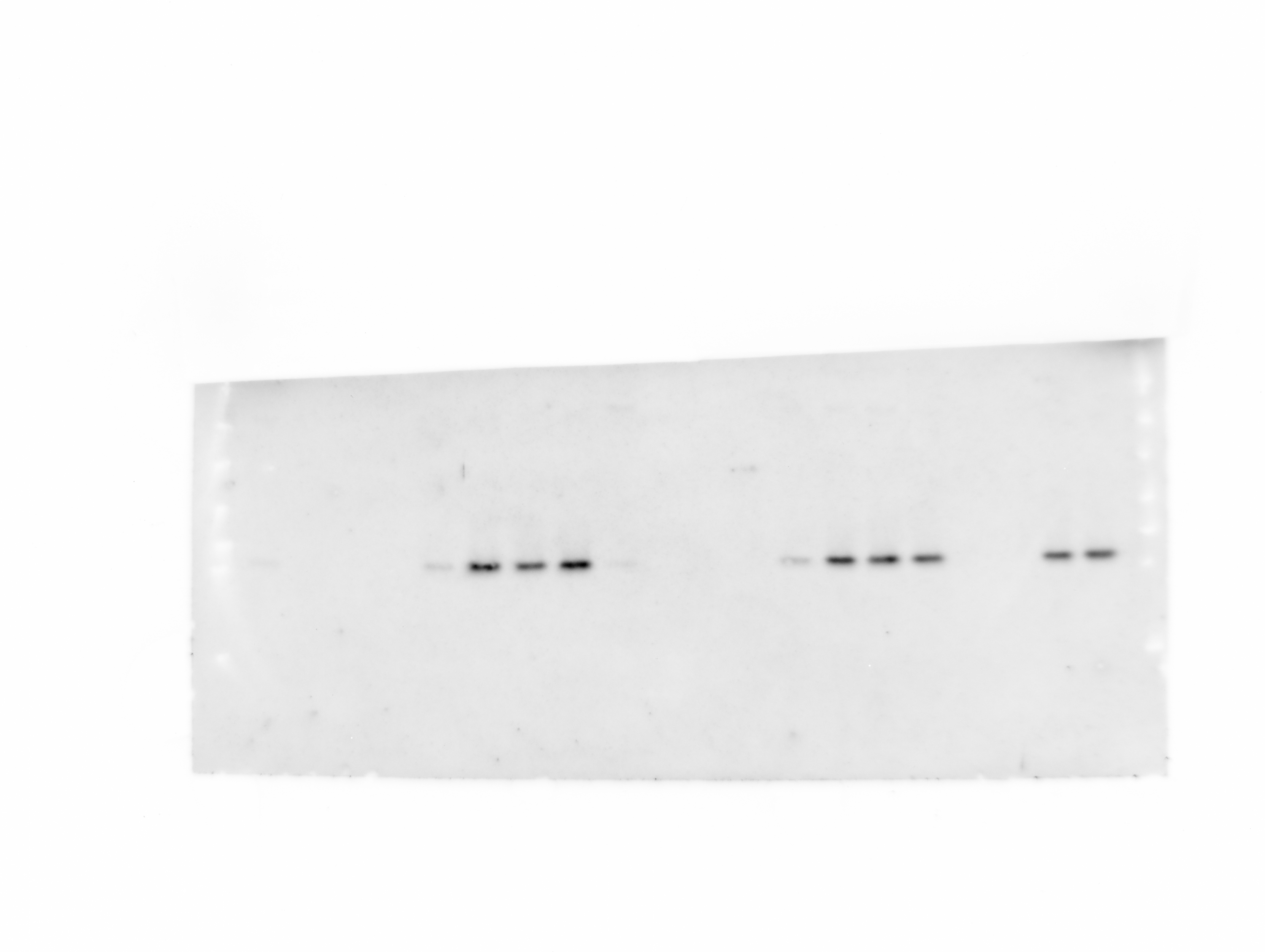

Supplement: Figure 7—source data 1. [file elife-74255-fig7-data1.zip › Figure 7D - source Rpl35.tif]

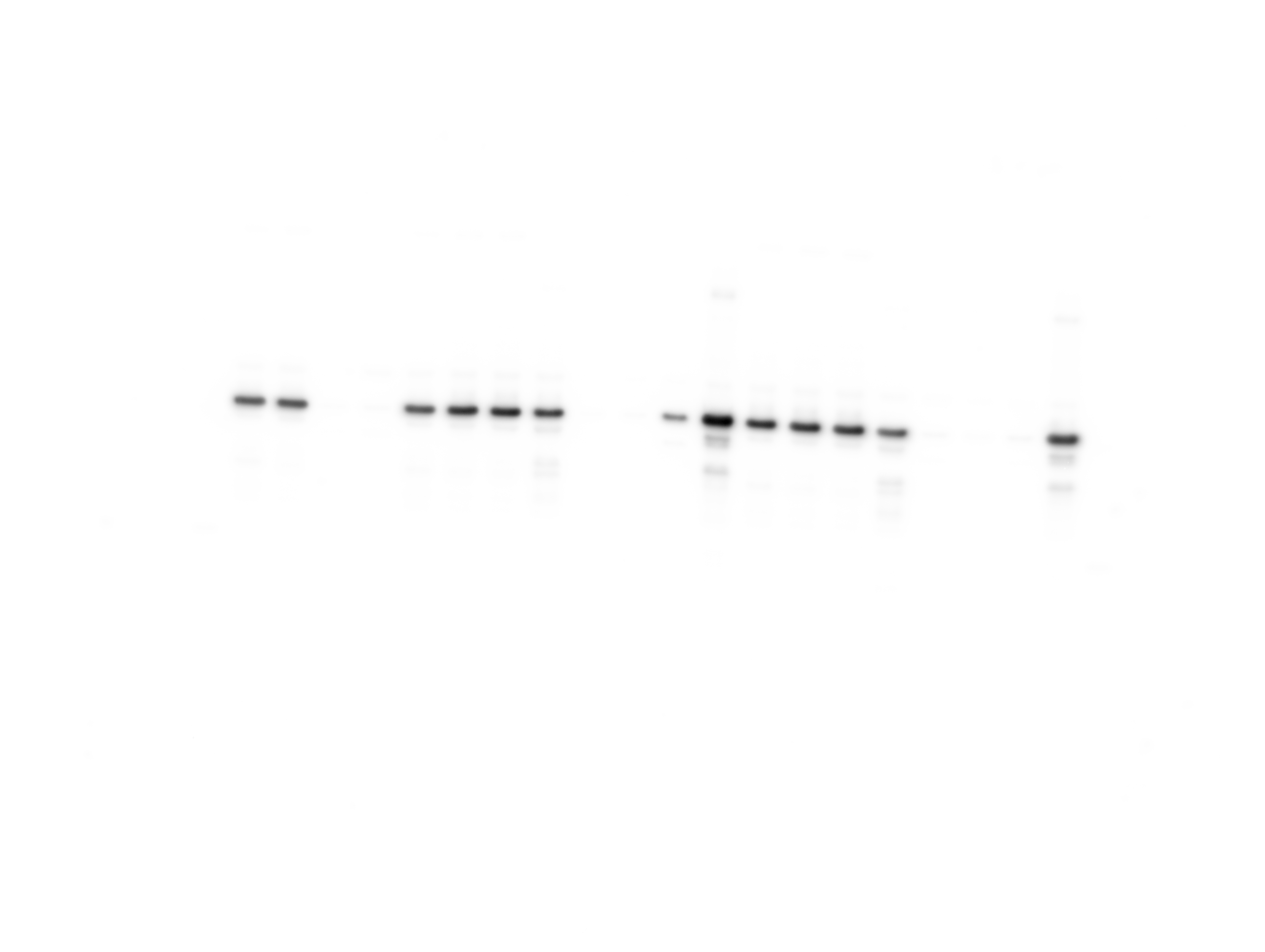

Supplement: Figure 7—source data 1. [file elife-74255-fig7-data1.zip › Figure 7D - source Rpl4.tif]

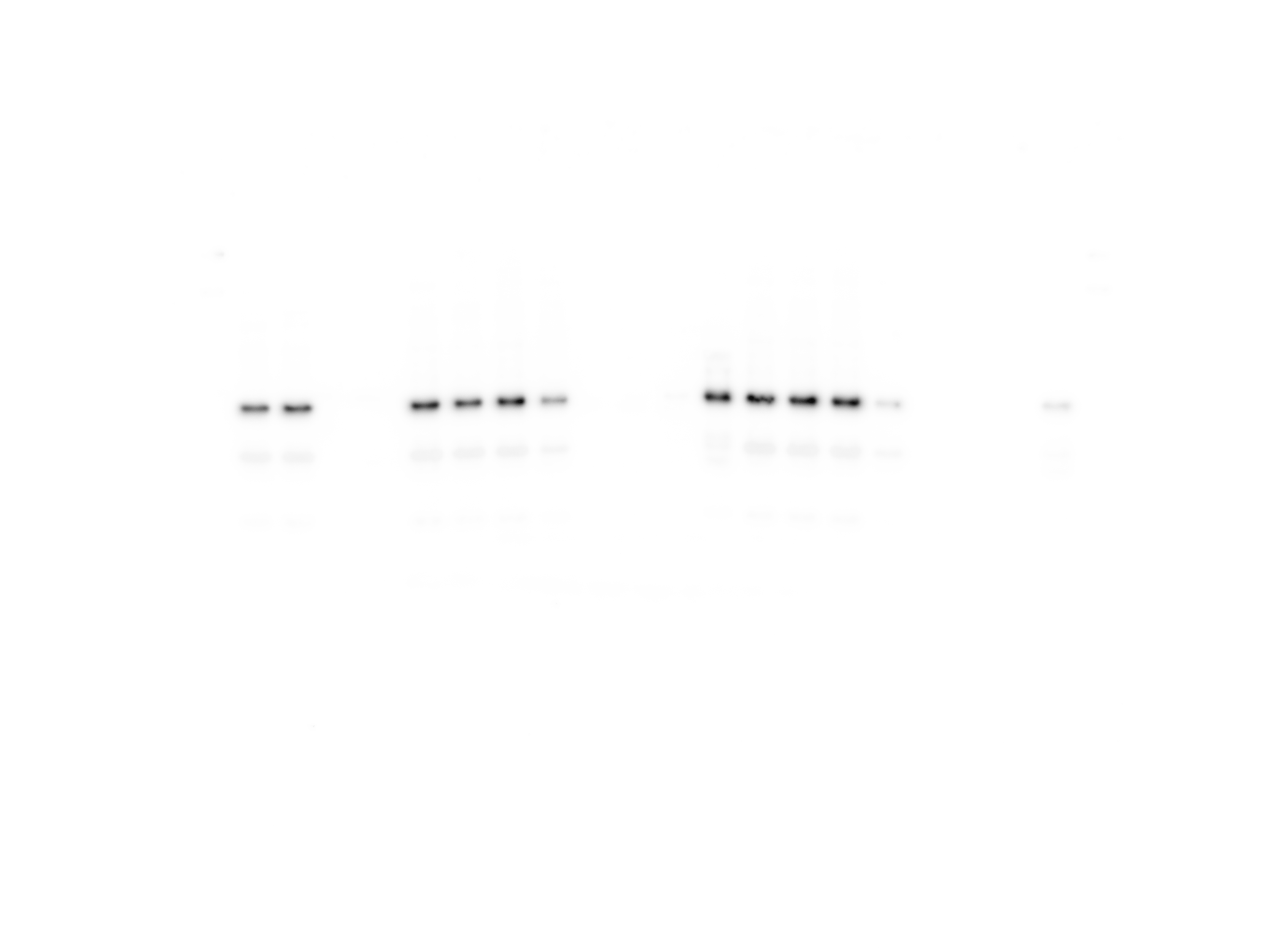

Supplement: Figure 7—source data 1. [file elife-74255-fig7-data1.zip › Figure 7D - source Rpl5.tif]

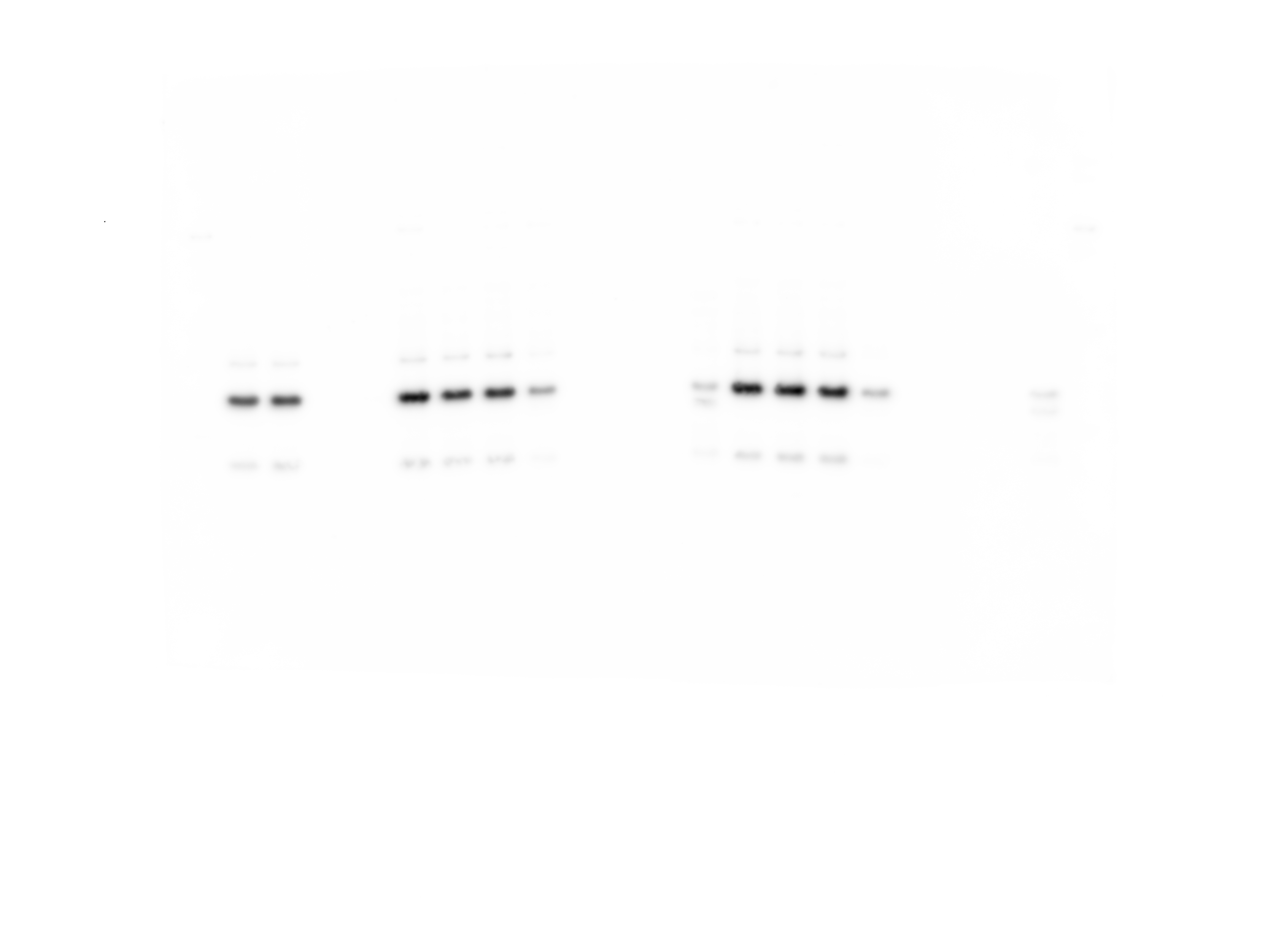

Supplement: Figure 7—source data 1. [file elife-74255-fig7-data1.zip › Figure 7D - source Rpp0.tif]

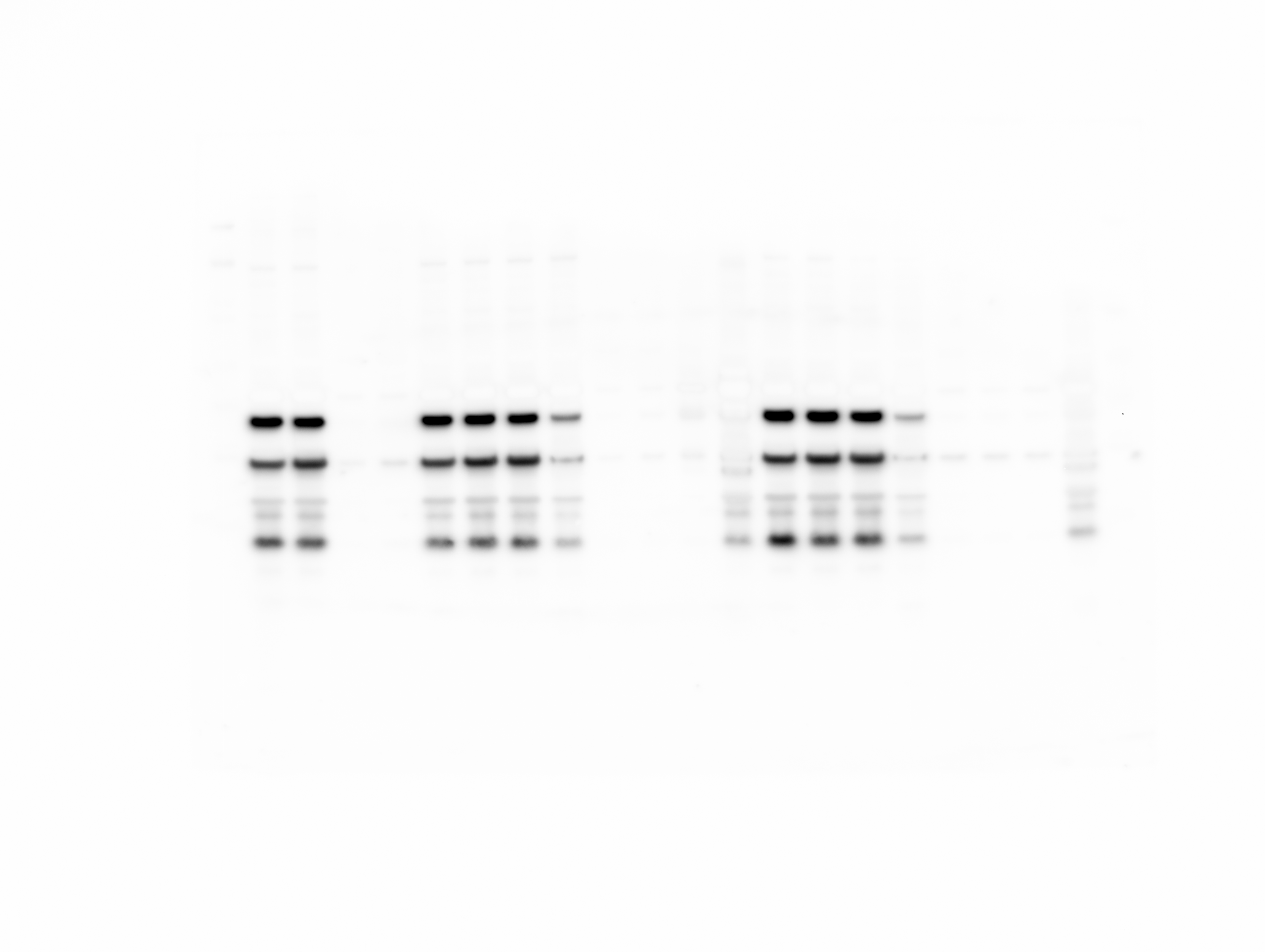

Supplement: Figure 7—source data 1. [file elife-74255-fig7-data1.zip › Figure 7D - source Rps26.tif]

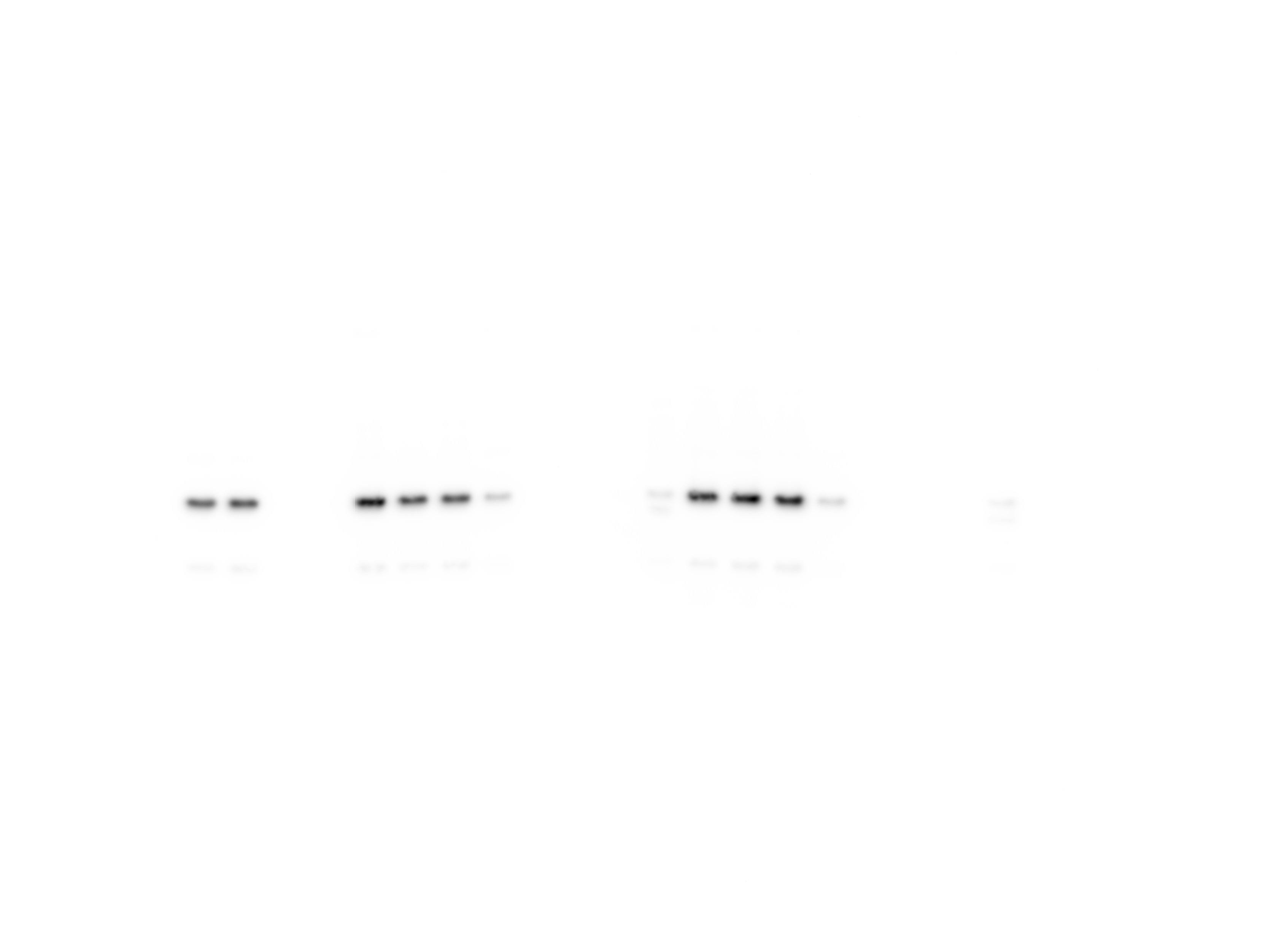

Supplement: Figure 7—source data 1. [file elife-74255-fig7-data1.zip › Figure 7D - source Rps3.tif]

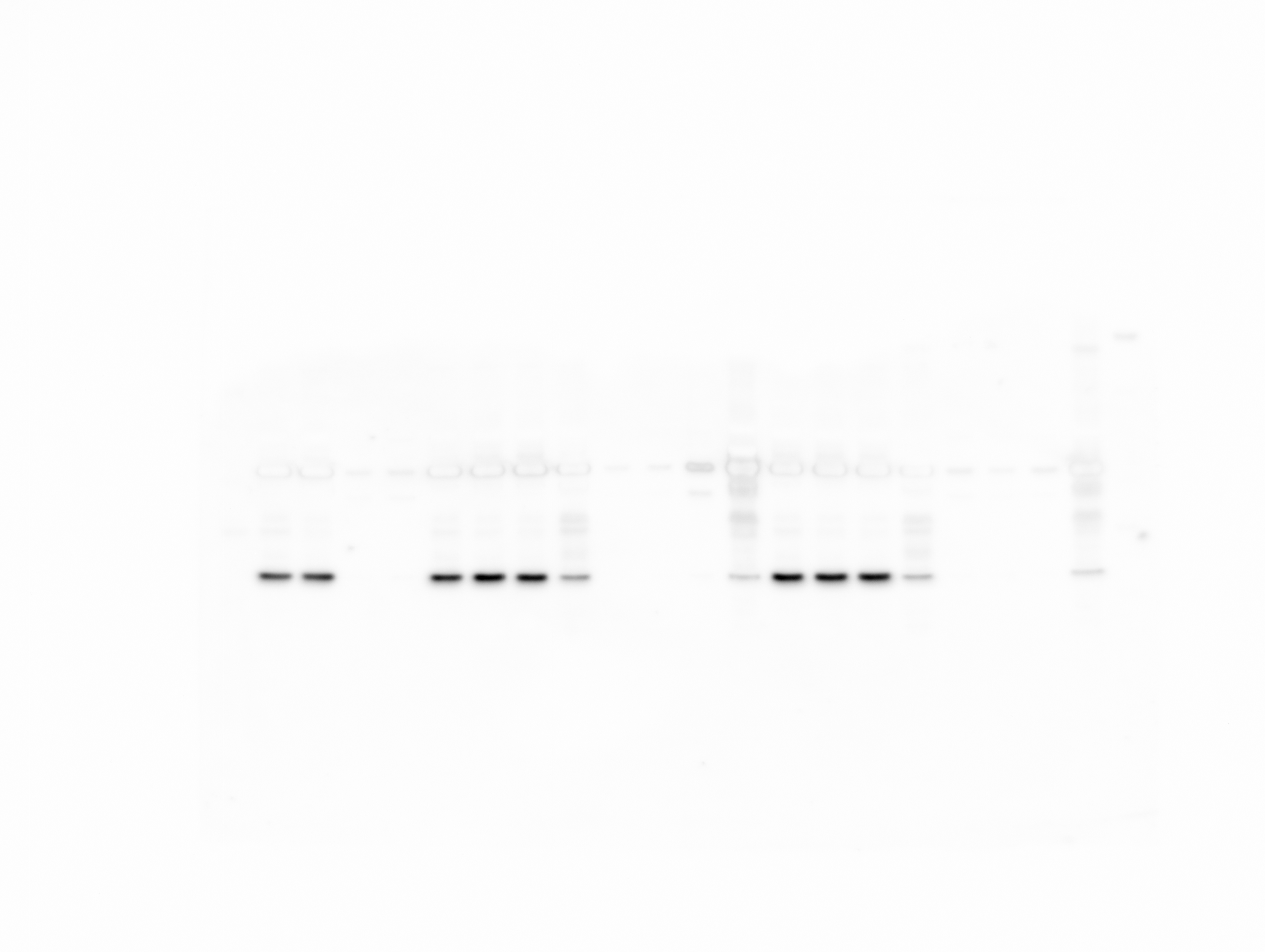

Supplement: Figure 7—source data 1. [file elife-74255-fig7-data1.zip › Figure 7D - source Rps9.tif]

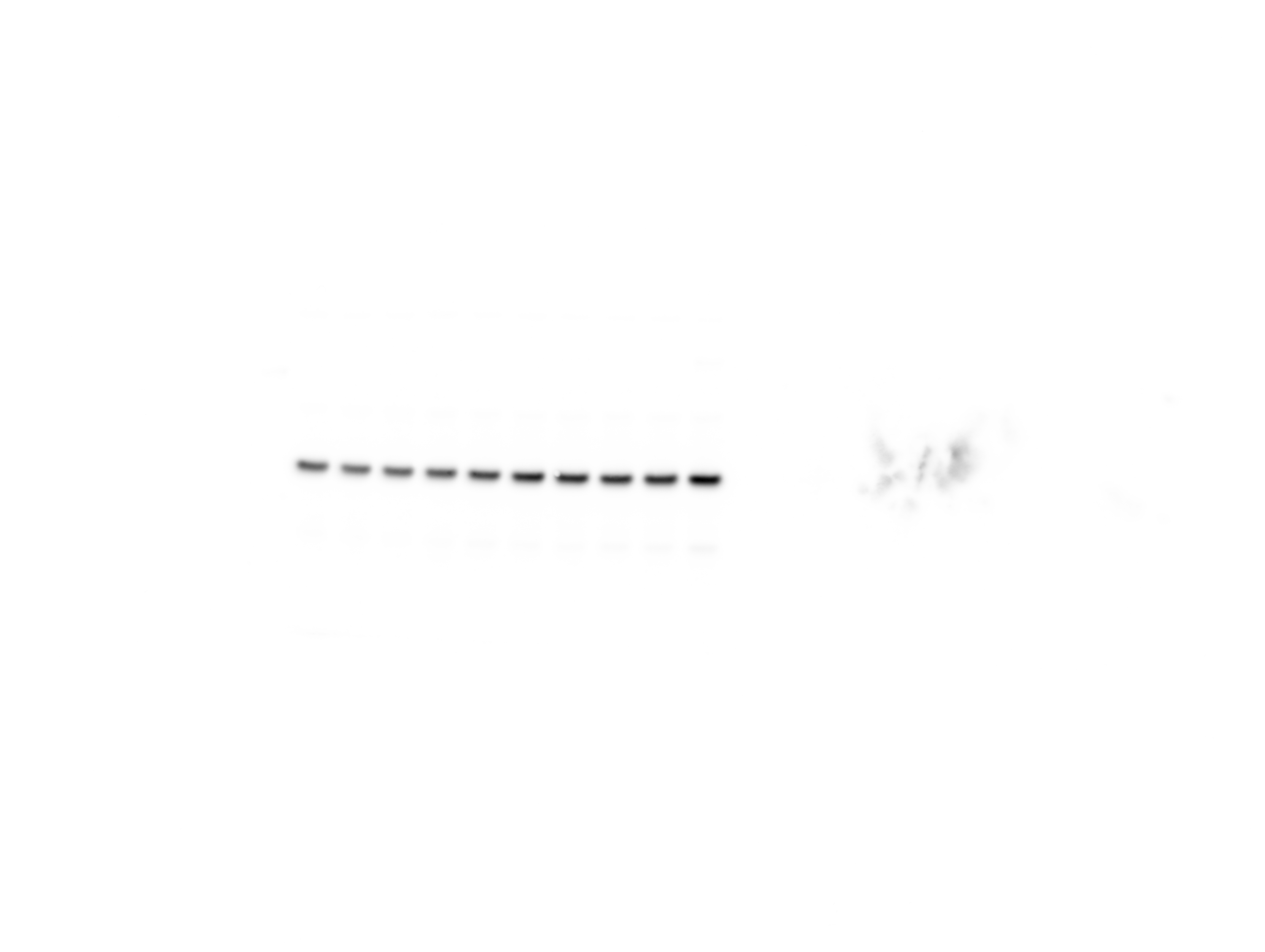

Supplement: Figure 7—figure supplement 2—source data 1. [file elife-74255-fig7-figsupp2-data1.zip › Figure 7-S2A - source Adh1.tif]

# Figure 7-S2A - source data

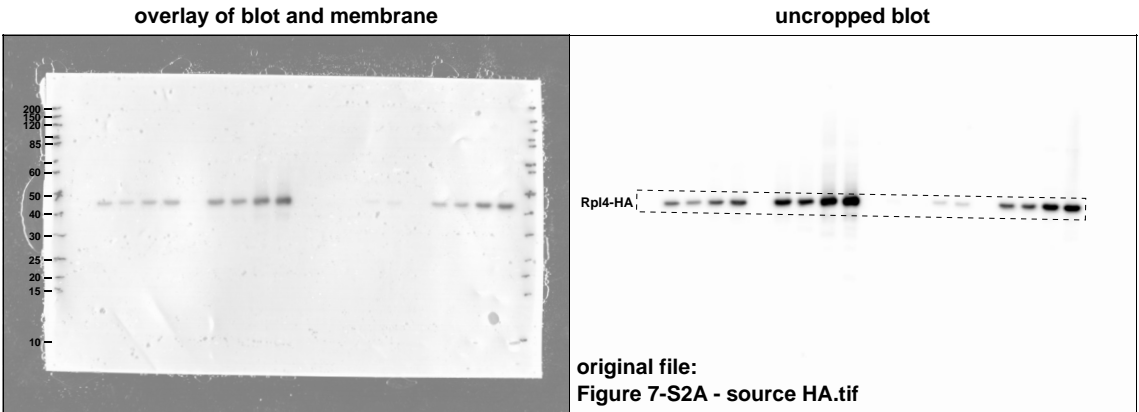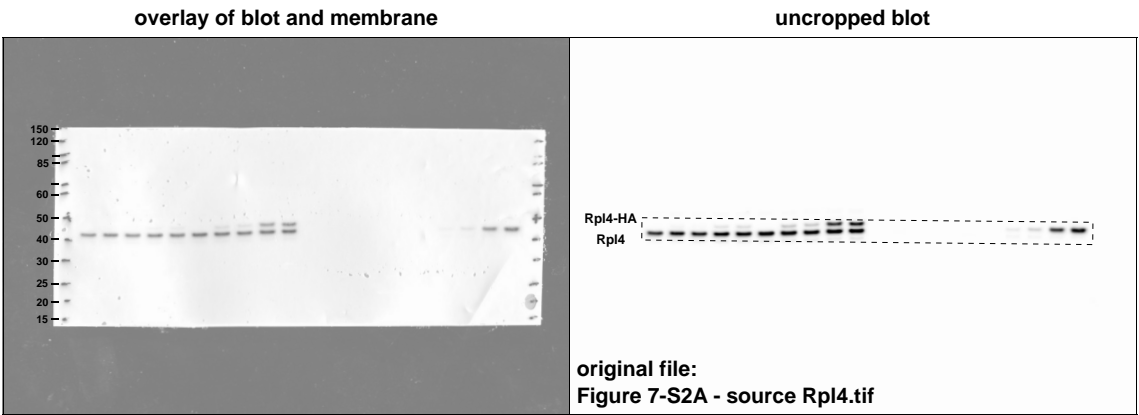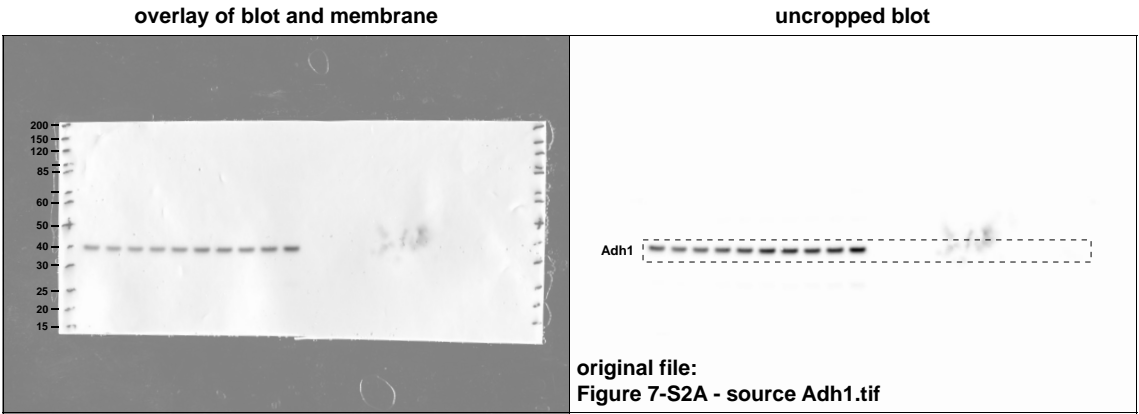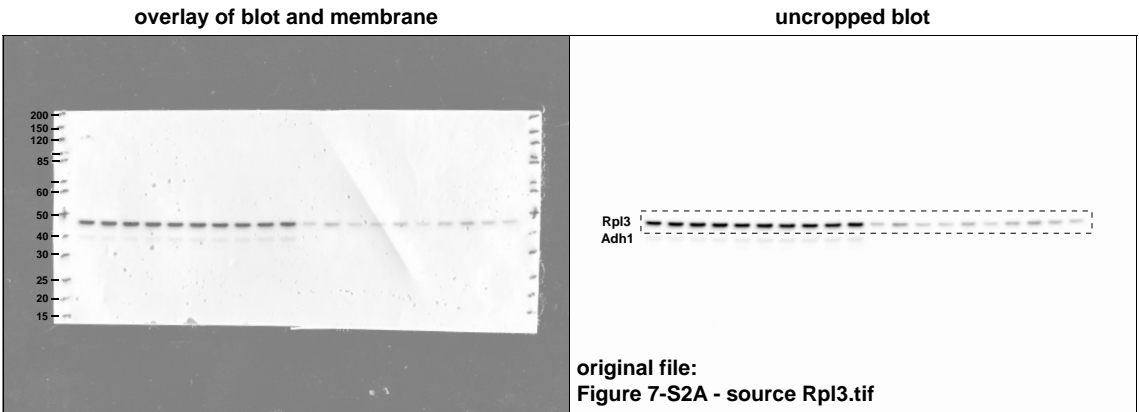

Supplement: Figure 7—figure supplement 2—source data 1. [file elife-74255-fig7-figsupp2-data1.zip › Figure 7-S2A - source data.pdf]

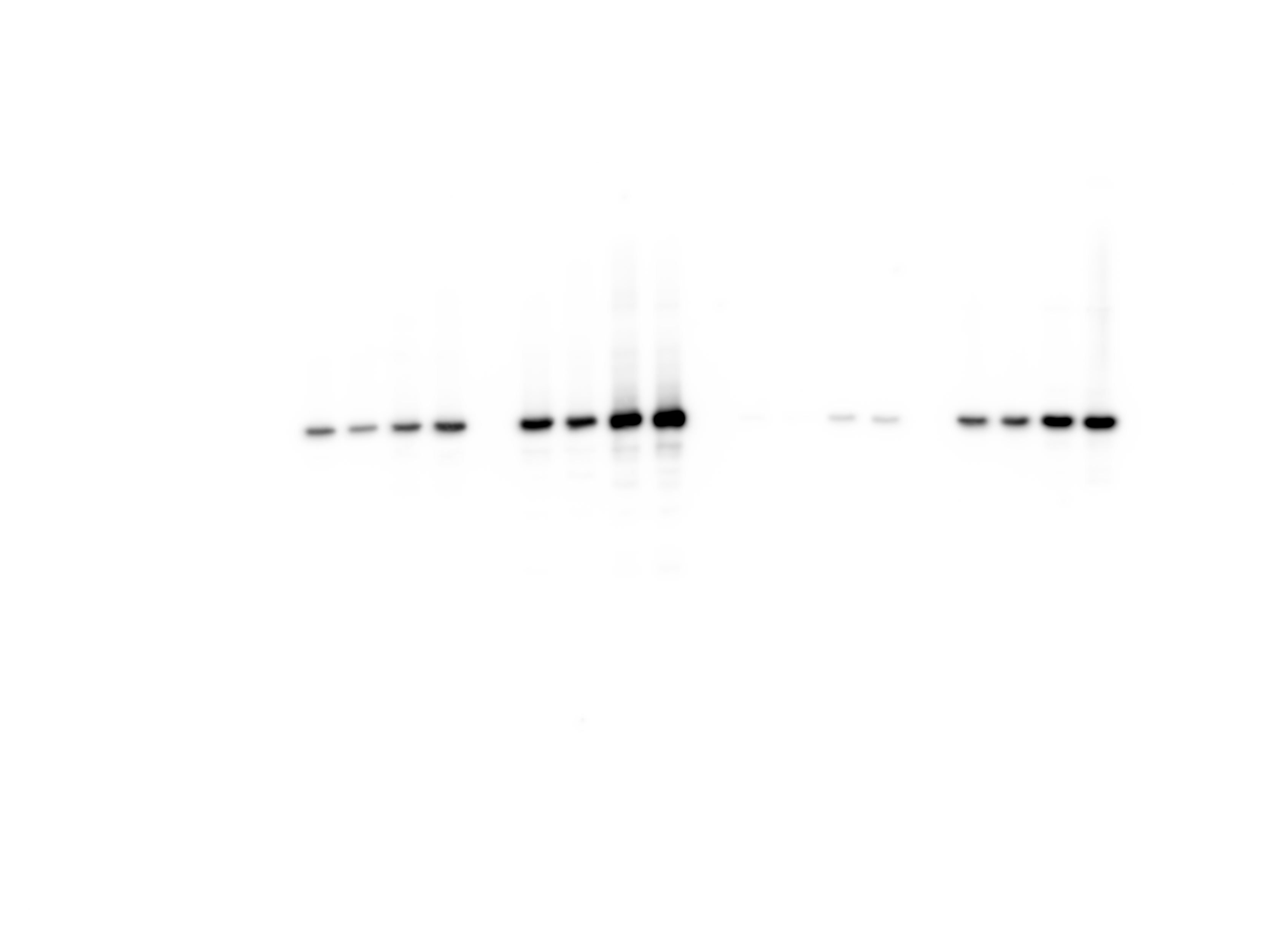

Supplement: Figure 7—figure supplement 2—source data 1. [file elife-74255-fig7-figsupp2-data1.zip › Figure 7-S2A - source HA.tif]

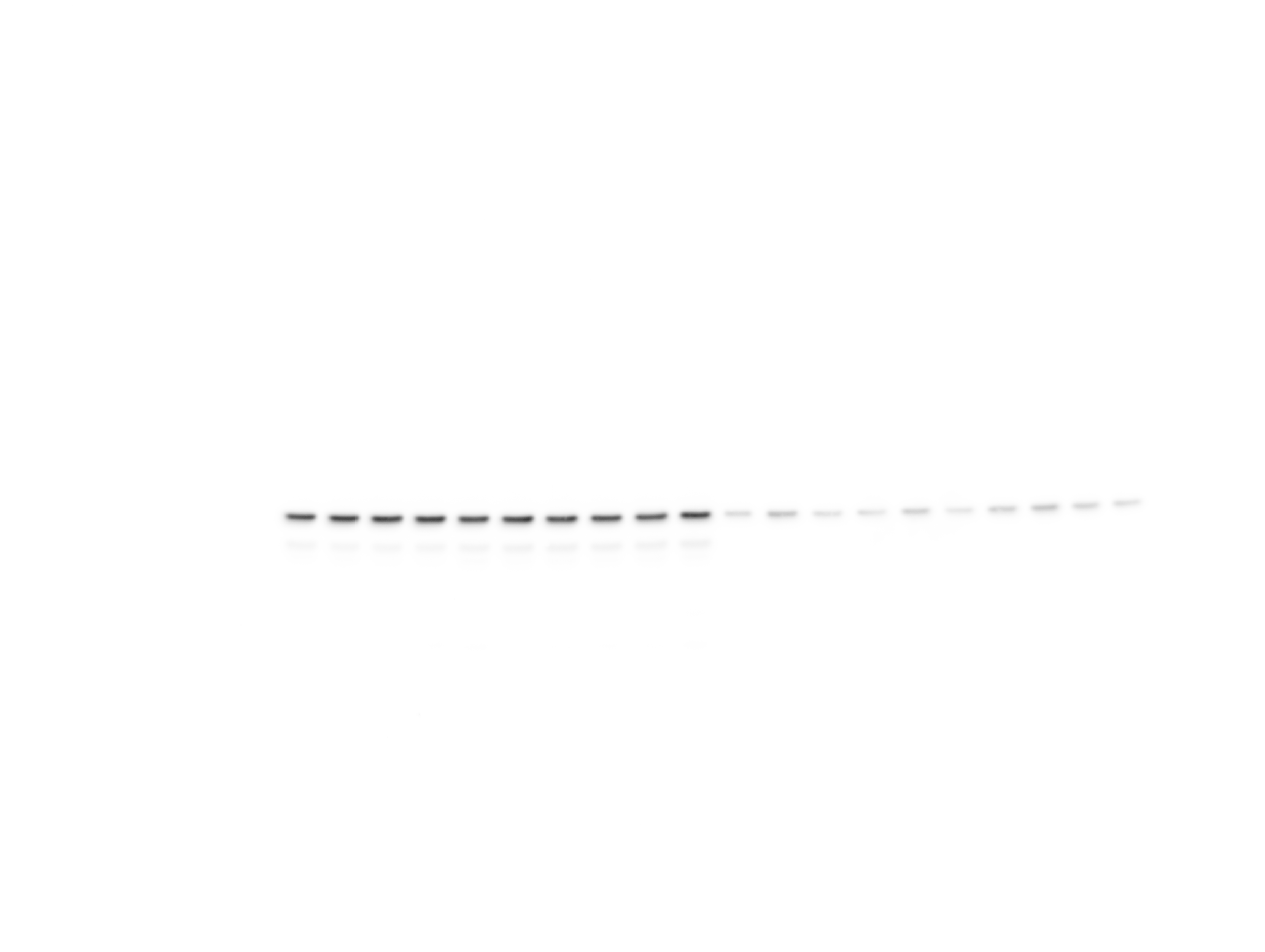

Supplement: Figure 7—figure supplement 2—source data 1. [file elife-74255-fig7-figsupp2-data1.zip › Figure 7-S2A - source Rpl3.tif]

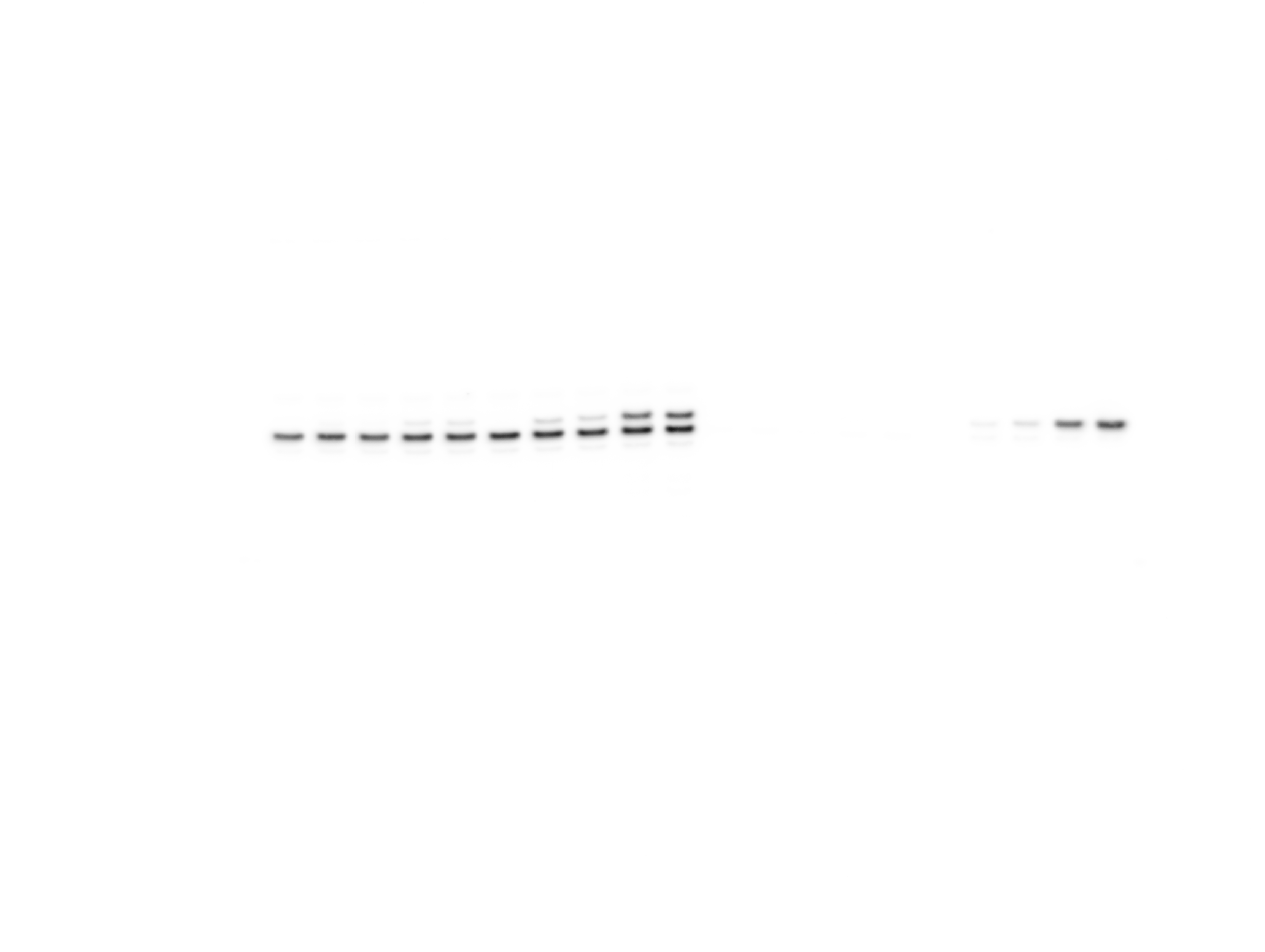

Supplement: Figure 7—figure supplement 2—source data 1. [file elife-74255-fig7-figsupp2-data1.zip › Figure 7-S2A - source Rpl4.tif]

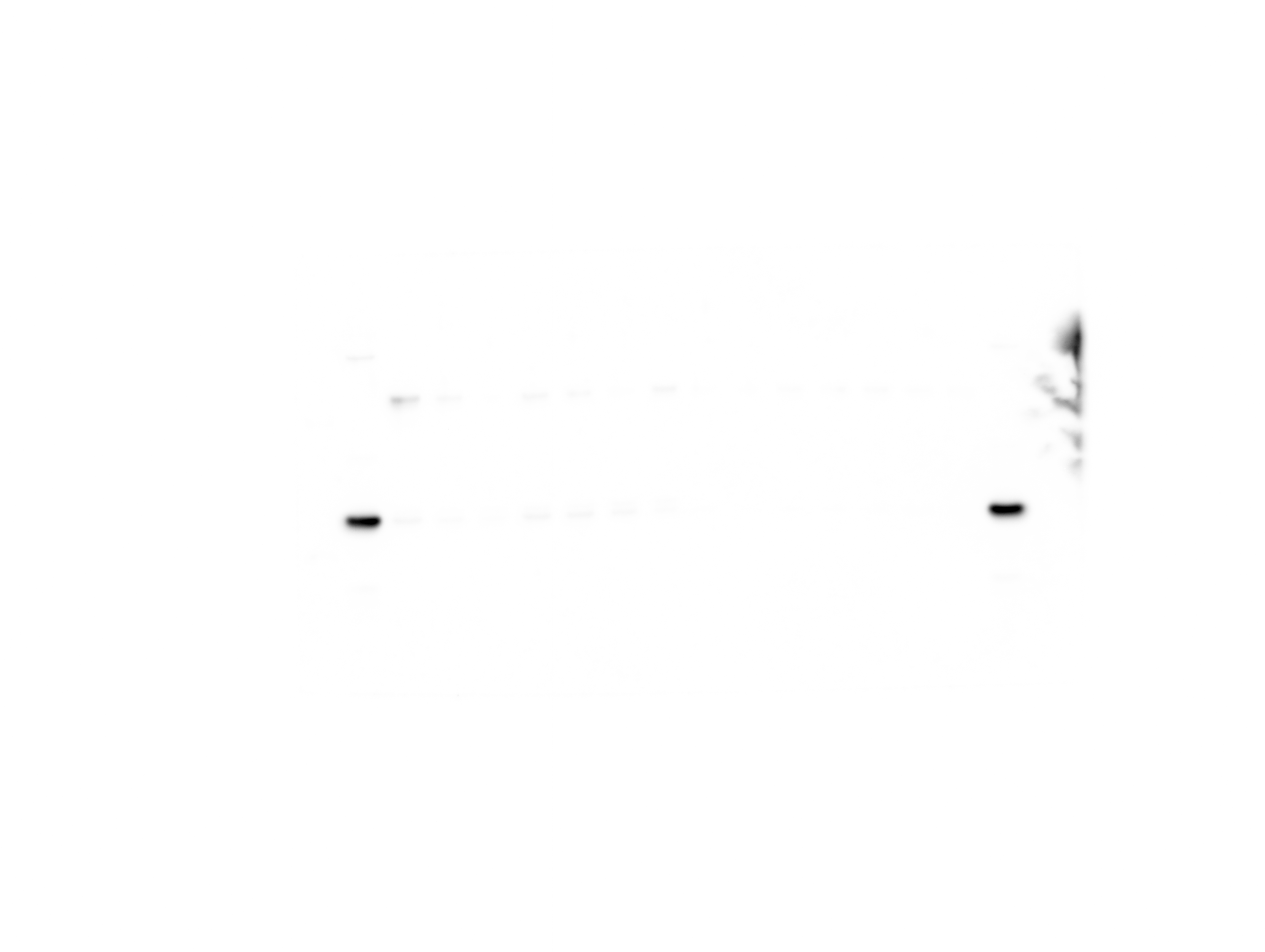

Supplement: Figure 7—figure supplement 2—source data 2. [file elife-74255-fig7-figsupp2-data2.zip › Figure 7-S2B - source Adh1 pellet.tif]

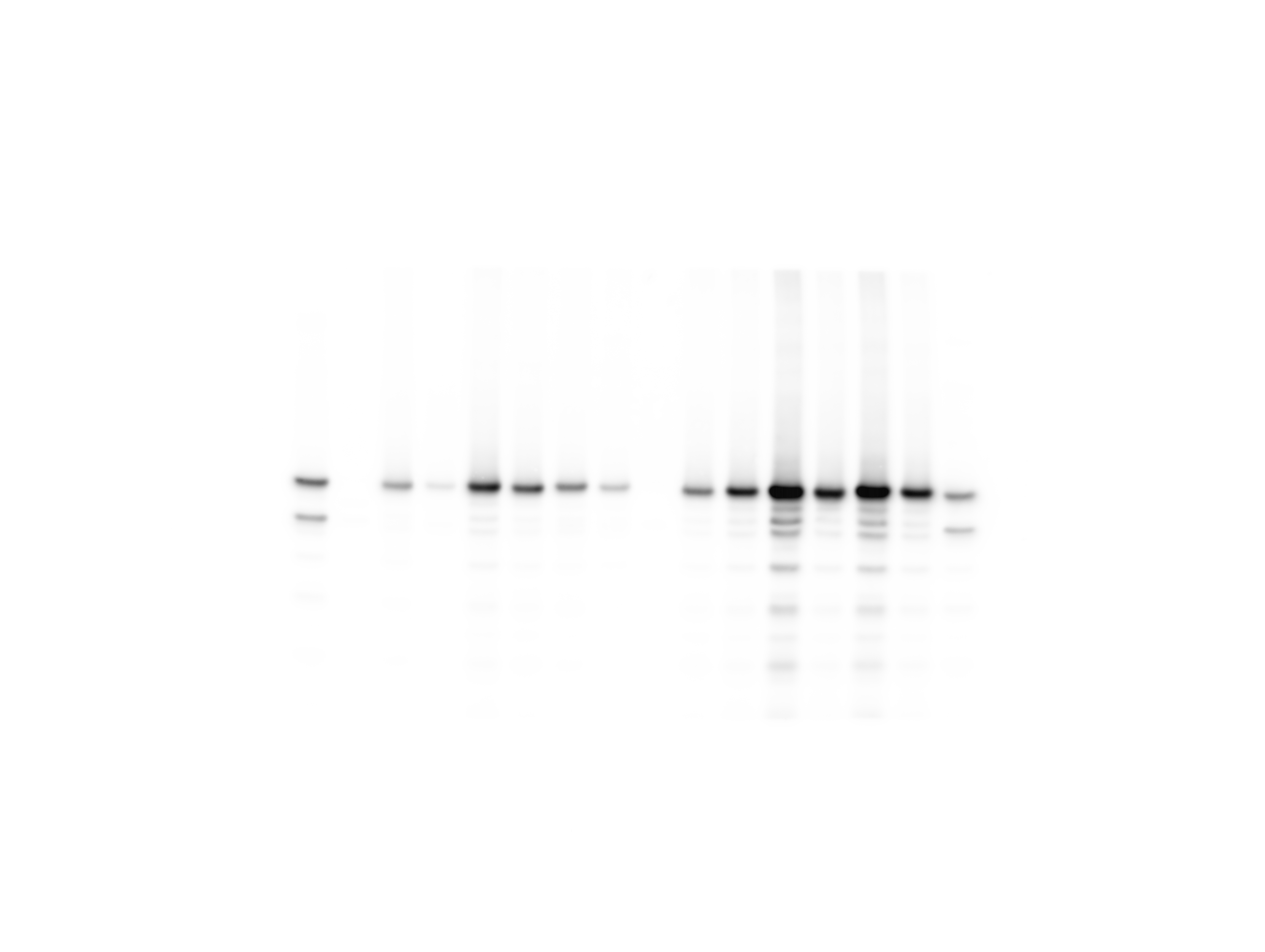

Supplement: Figure 7—figure supplement 2—source data 2. [file elife-74255-fig7-figsupp2-data2.zip › Figure 7-S2B - source HA pellet.tif]

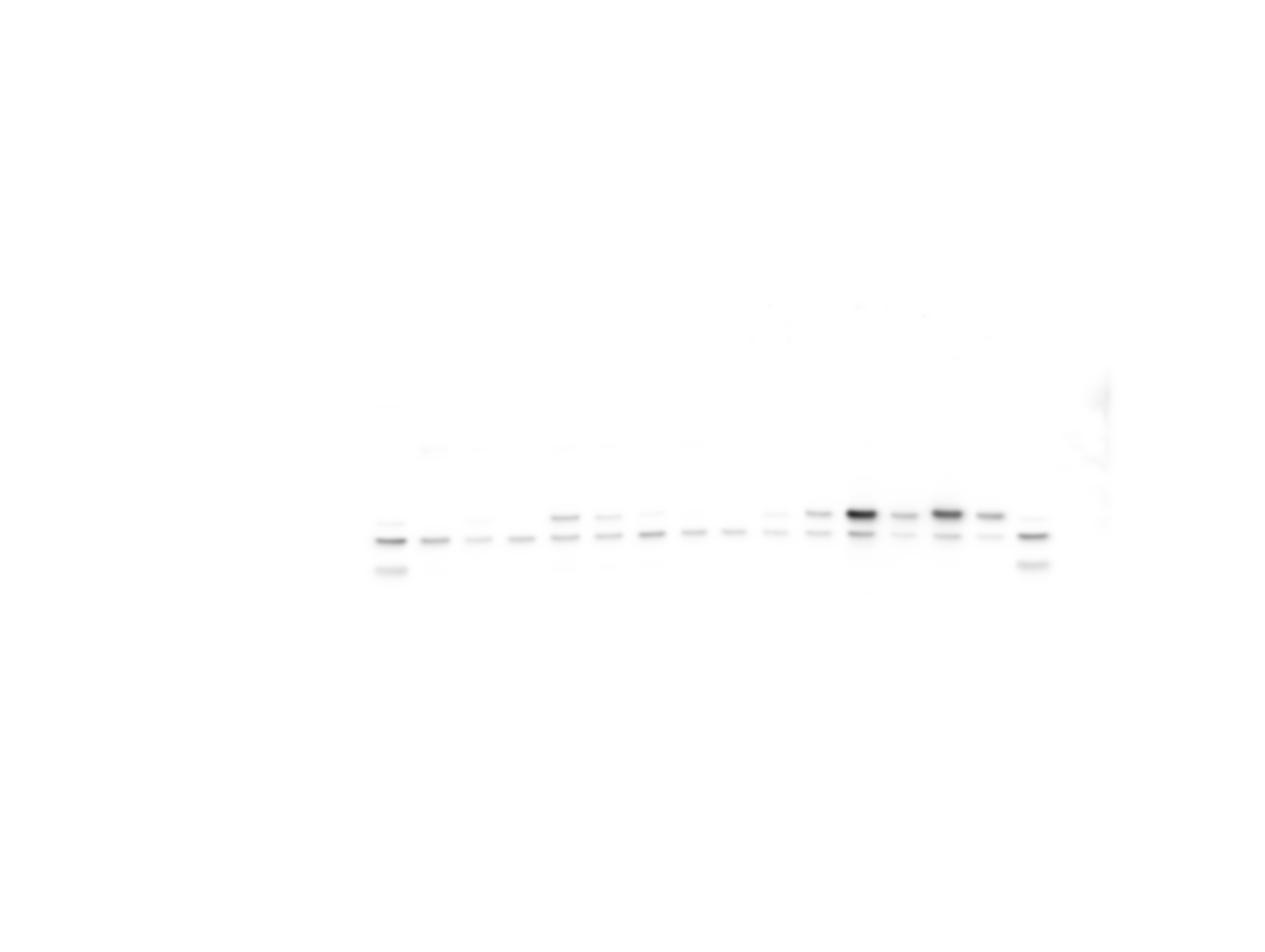

Supplement: Figure 7—figure supplement 2—source data 2. [file elife-74255-fig7-figsupp2-data2.zip › Figure 7-S2B - source Rpl3 pellet.tif]

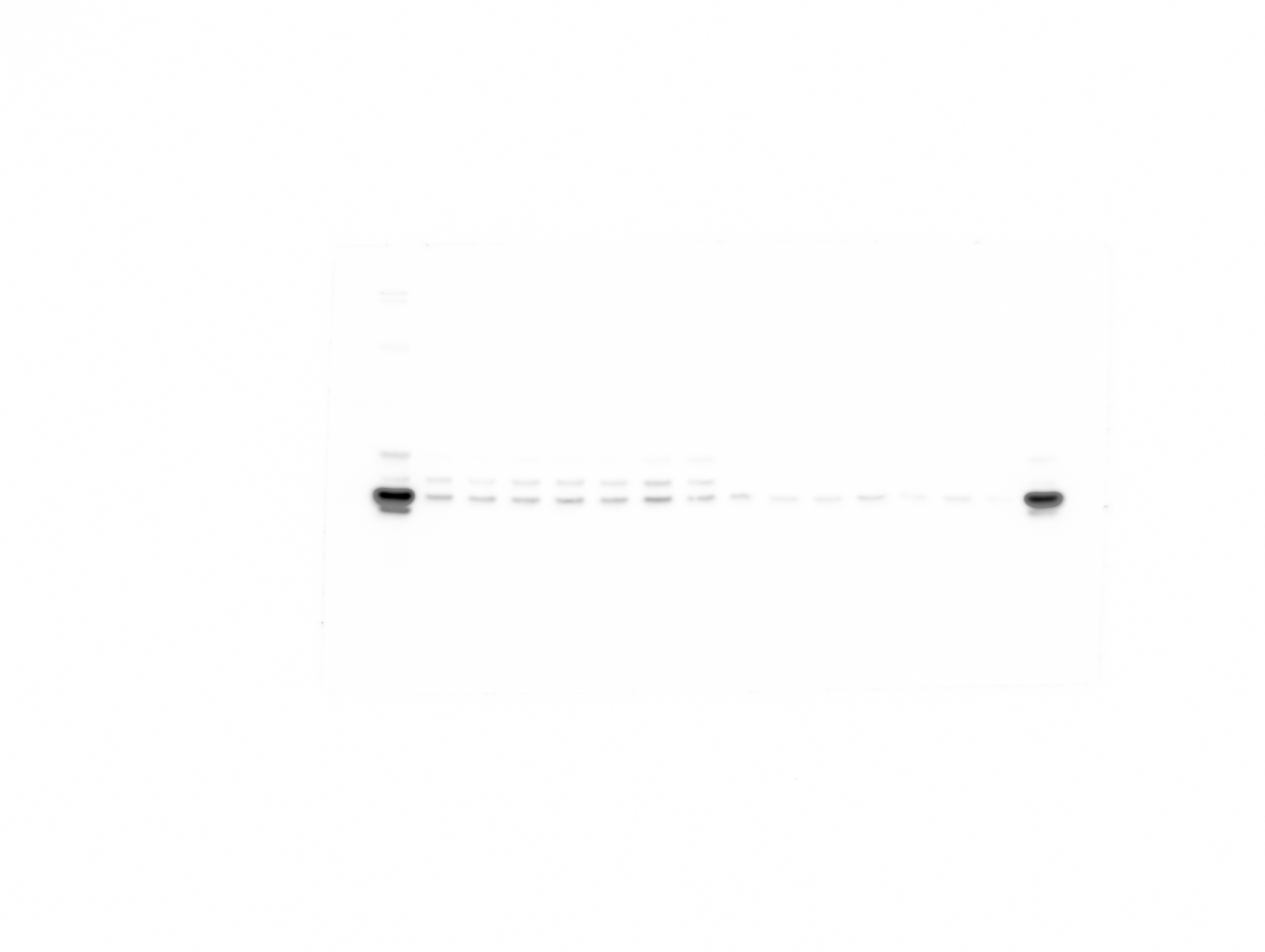

Supplement: Figure 7—figure supplement 2—source data 2. [file elife-74255-fig7-figsupp2-data2.zip › Figure 7-S2B - source Rpl4 pellet.tif]

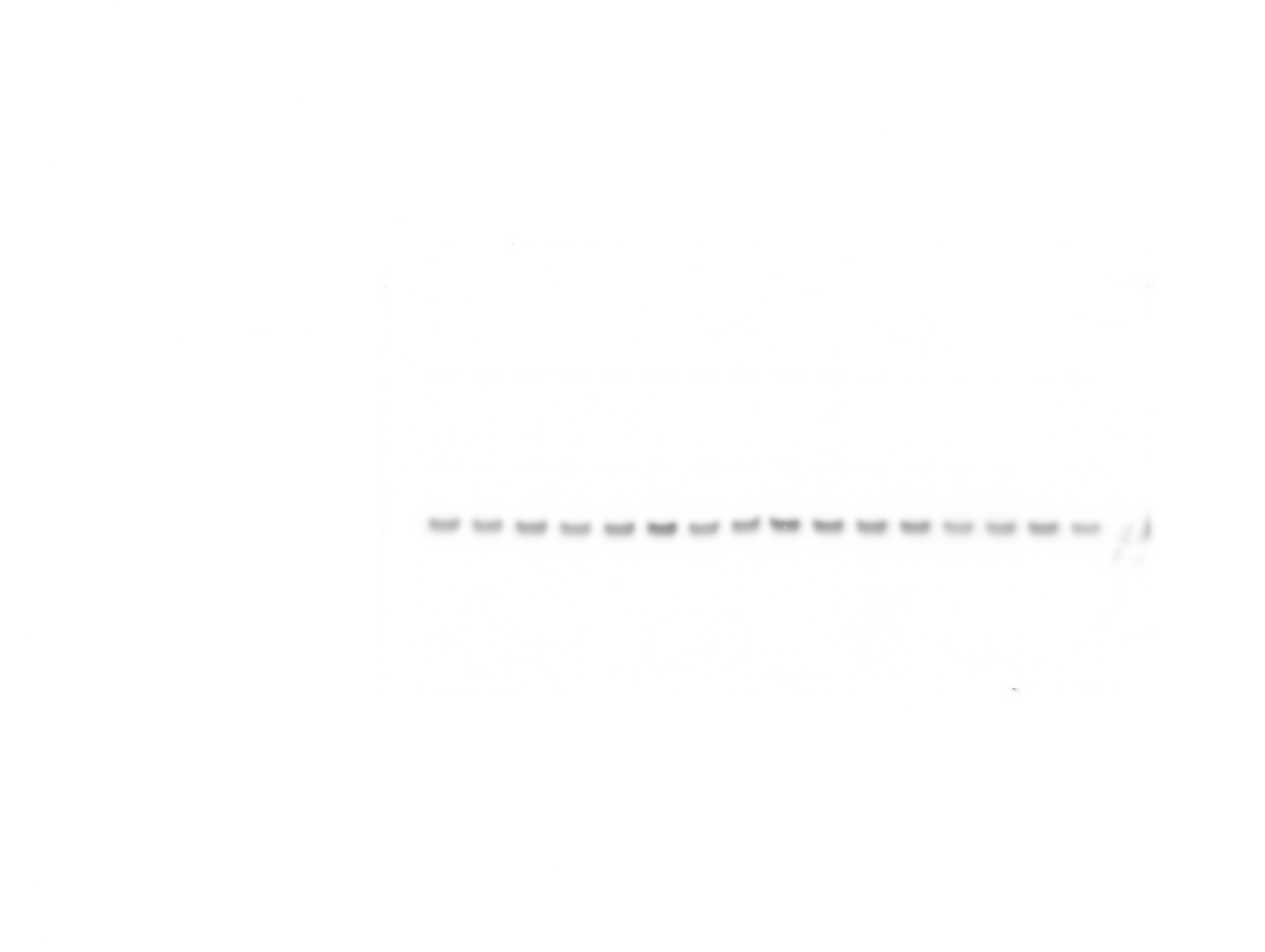

Supplement: Figure 7—figure supplement 2—source data 2. [file elife-74255-fig7-figsupp2-data2.zip › Figure 7-S2B - source Adh1 TE.tif]

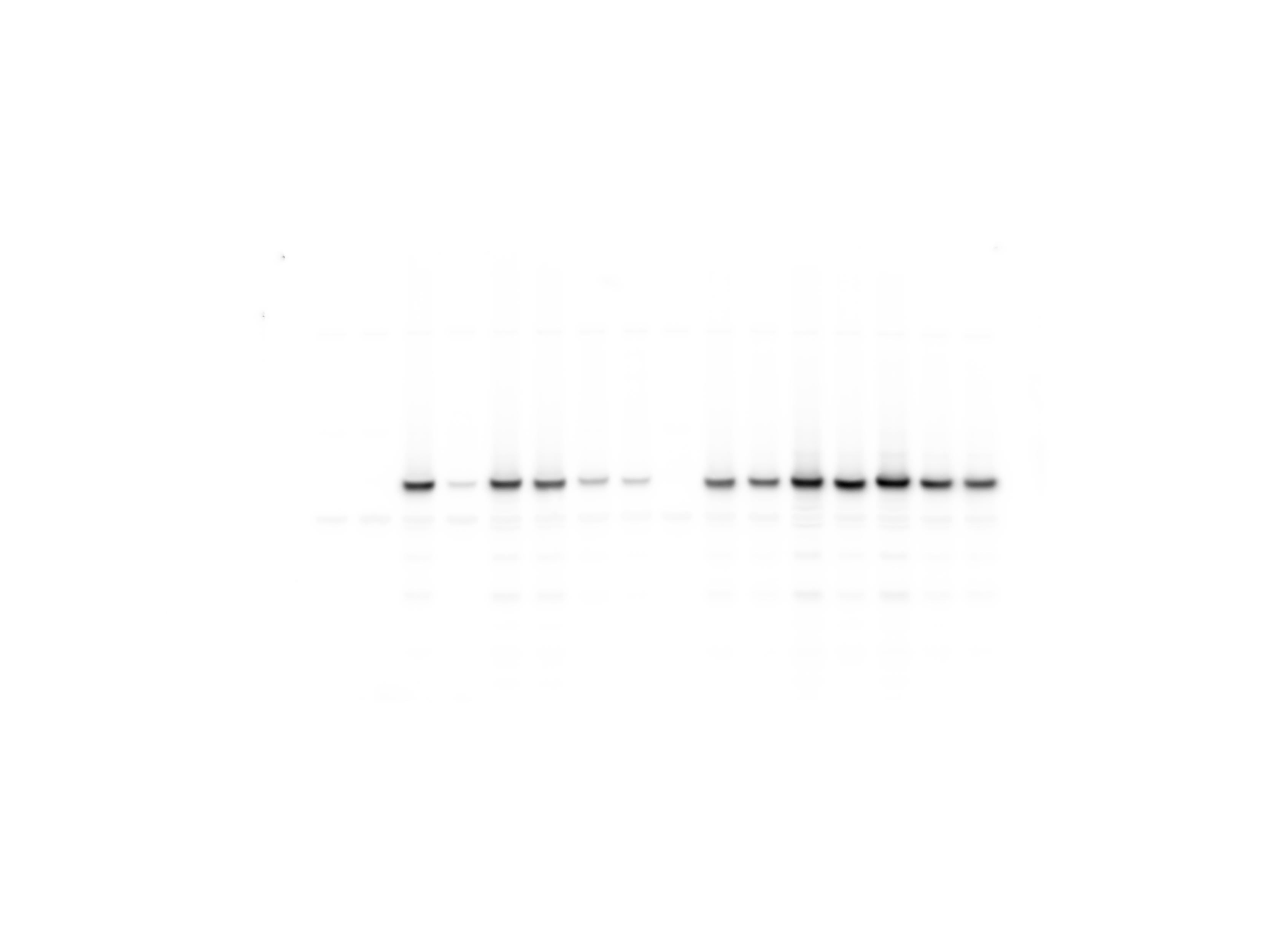

Supplement: Figure 7—figure supplement 2—source data 2. [file elife-74255-fig7-figsupp2-data2.zip › Figure 7S2B - source HA TE.tif]

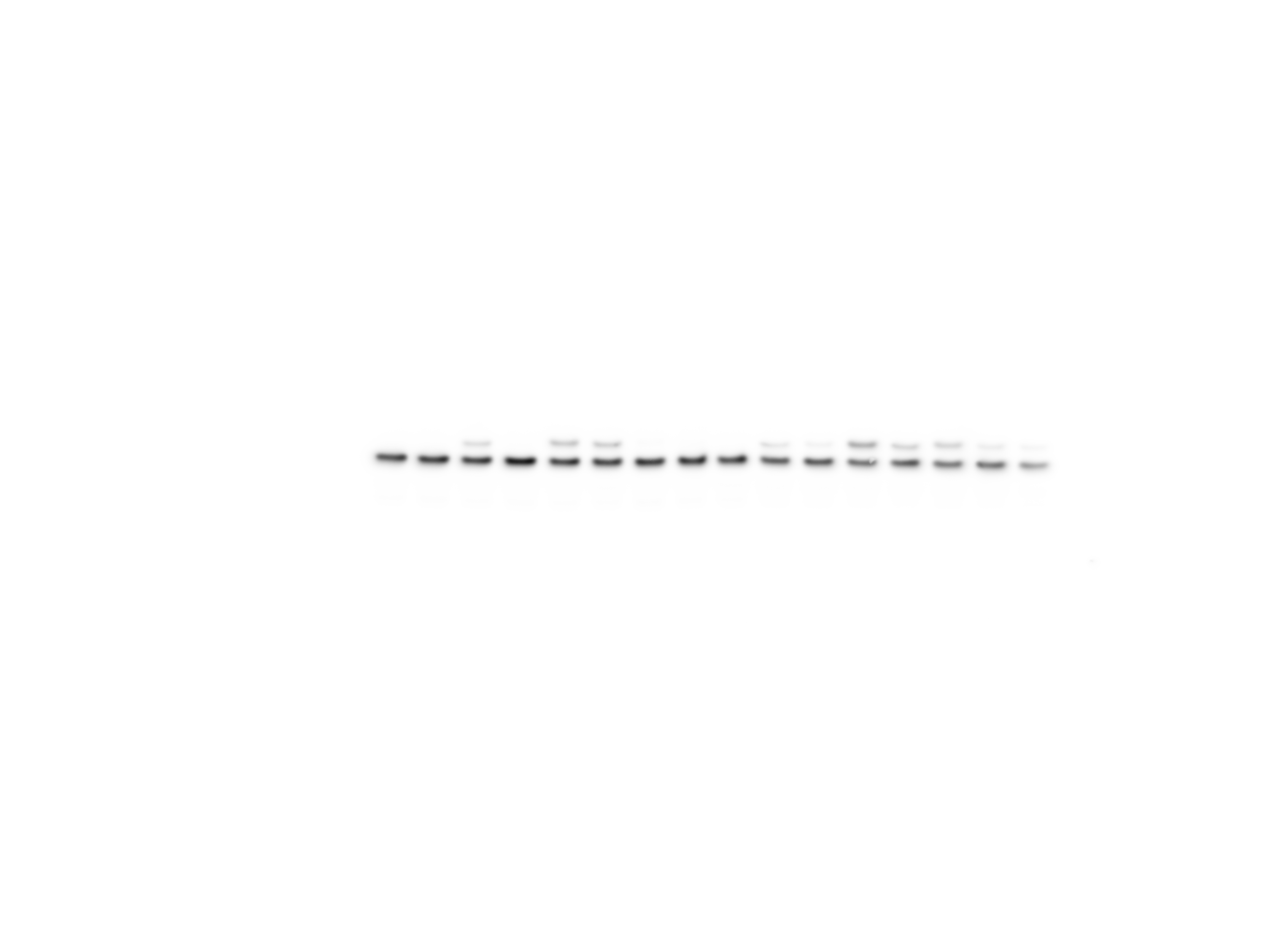

Supplement: Figure 7—figure supplement 2—source data 2. [file elife-74255-fig7-figsupp2-data2.zip › Figure 7-S2B - source Rpl3 TE.tif]

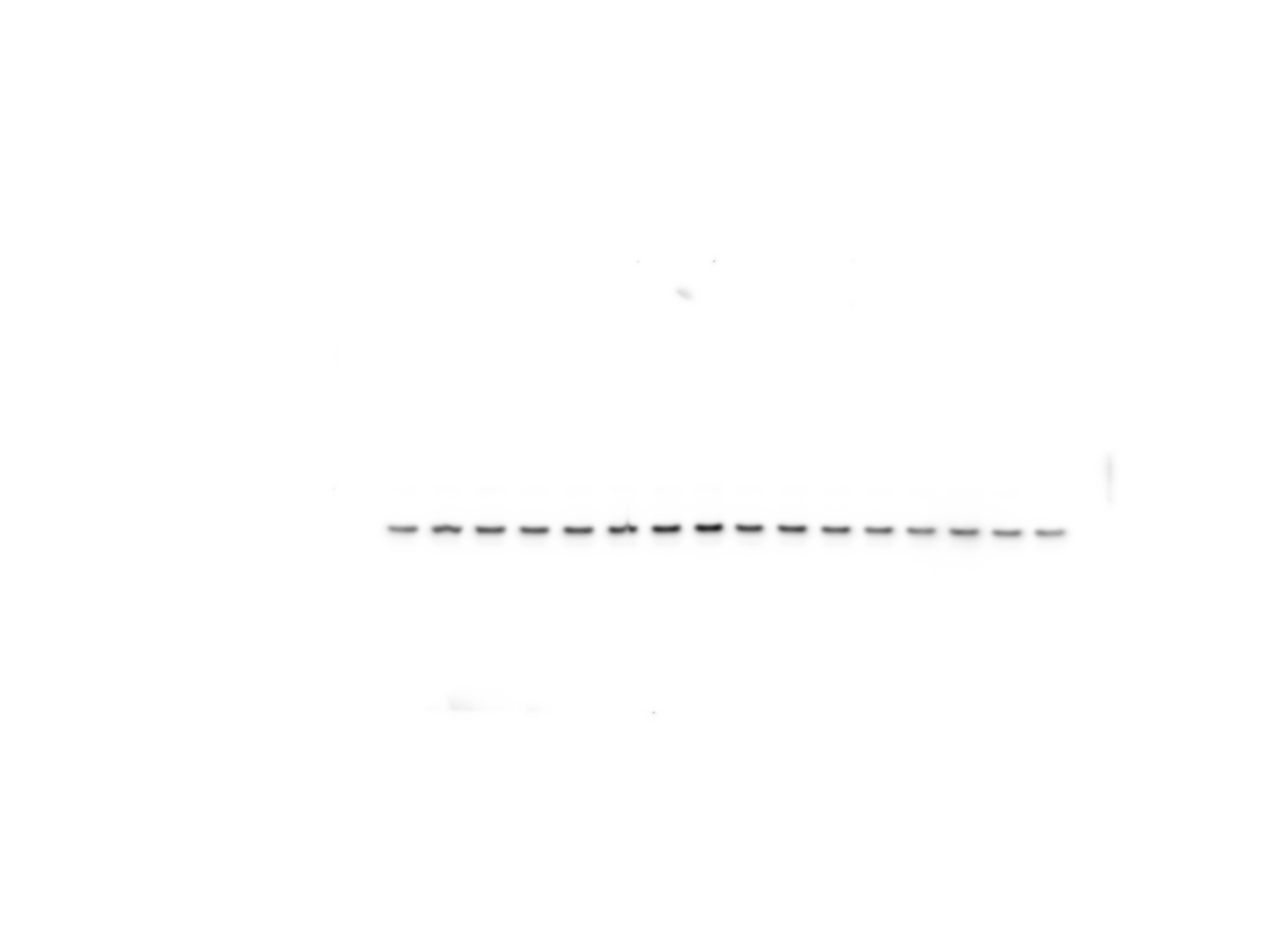

Supplement: Figure 7—figure supplement 2—source data 2. [file elife-74255-fig7-figsupp2-data2.zip › Figure 7-S2B - source Rpl4 TE.tif]

# Figure 7-S2B (total extract) - source data

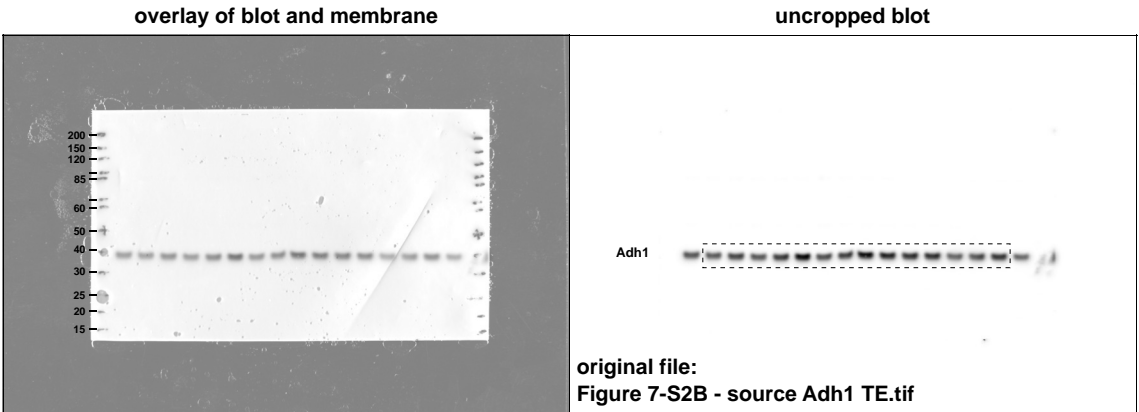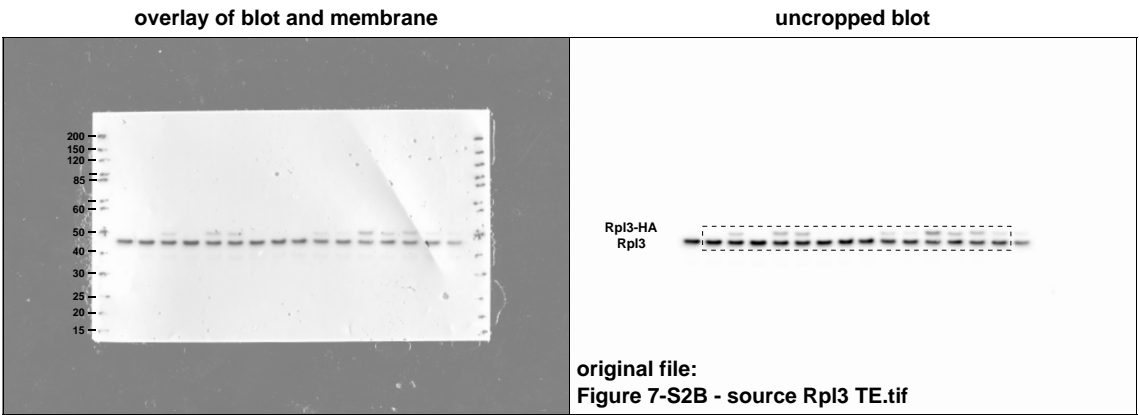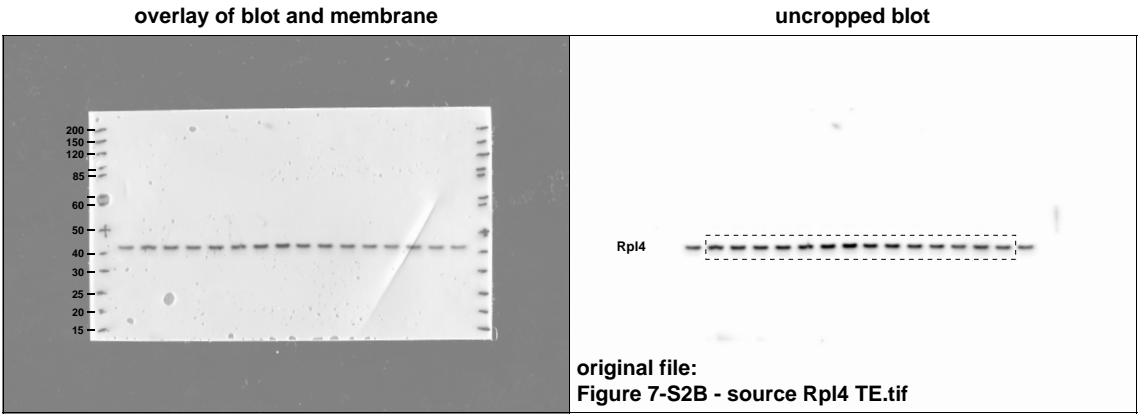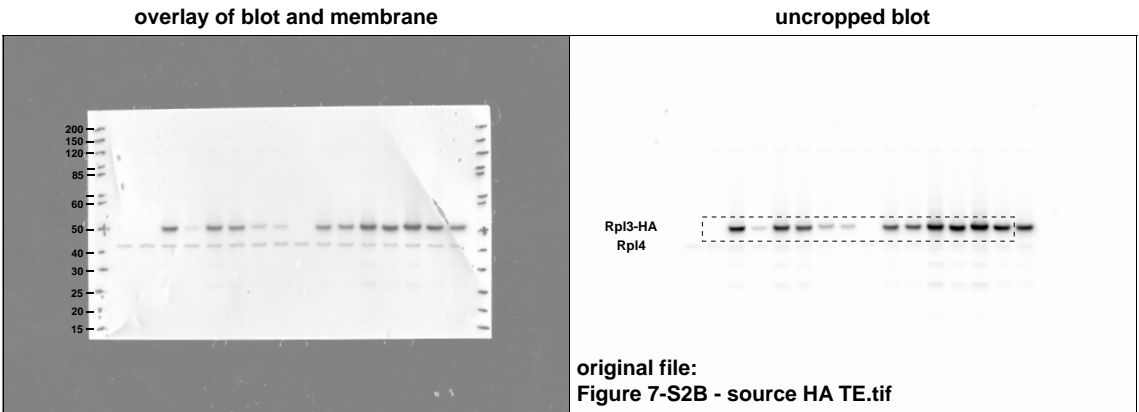

# Figure 7-S2B (pellet) - source data

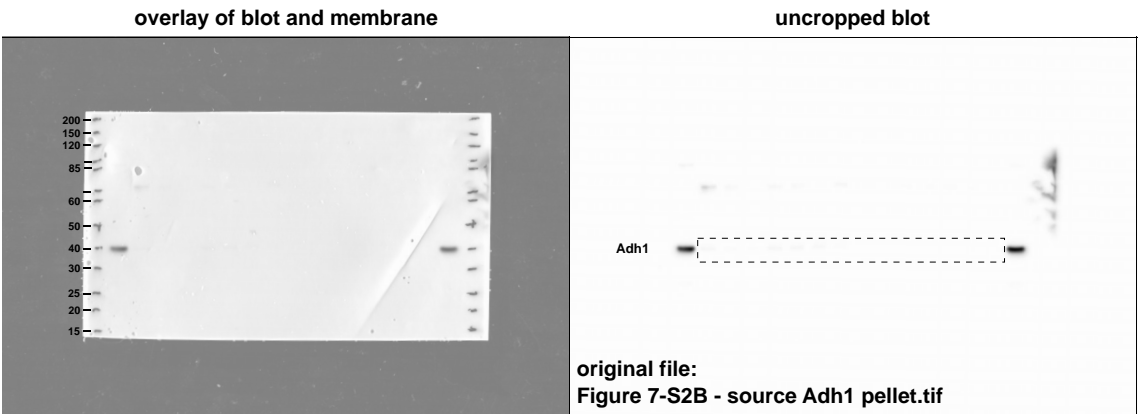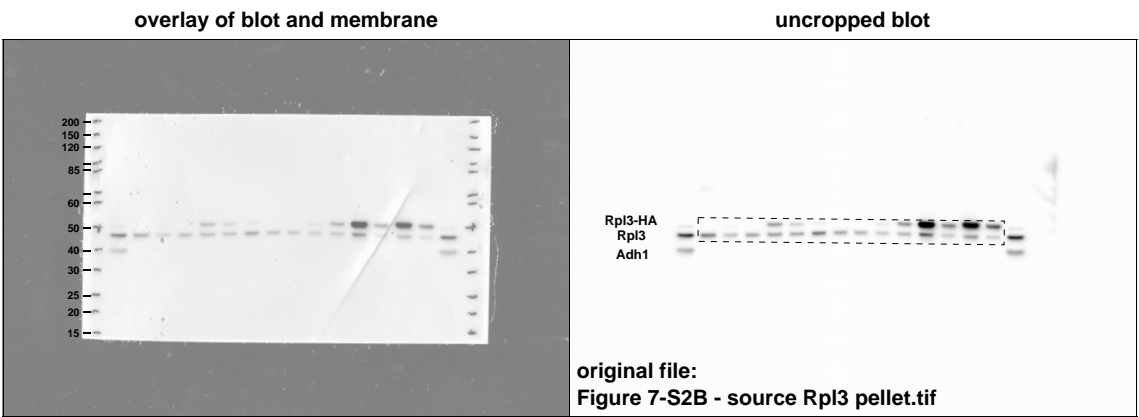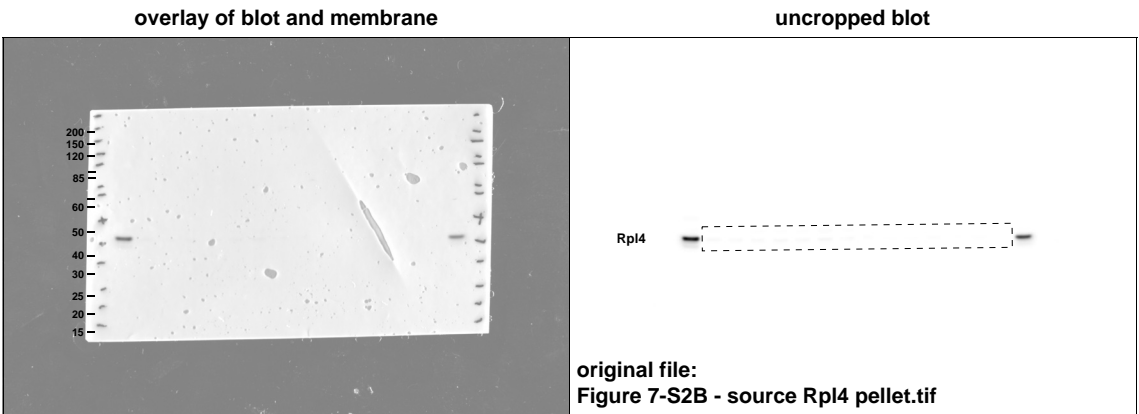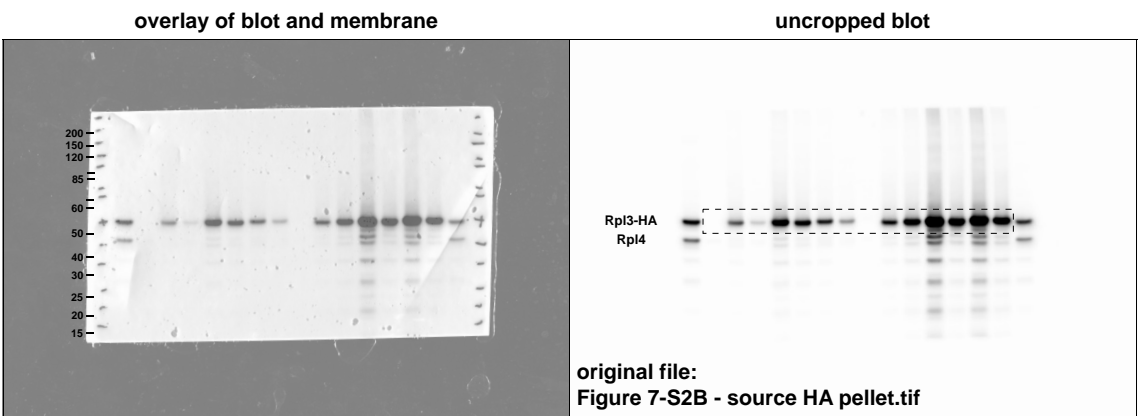

Supplement: Figure 7—figure supplement 2—source data 2. [file elife-74255-fig7-figsupp2-data2.zip › Figure 7-S2B - source data.pdf]

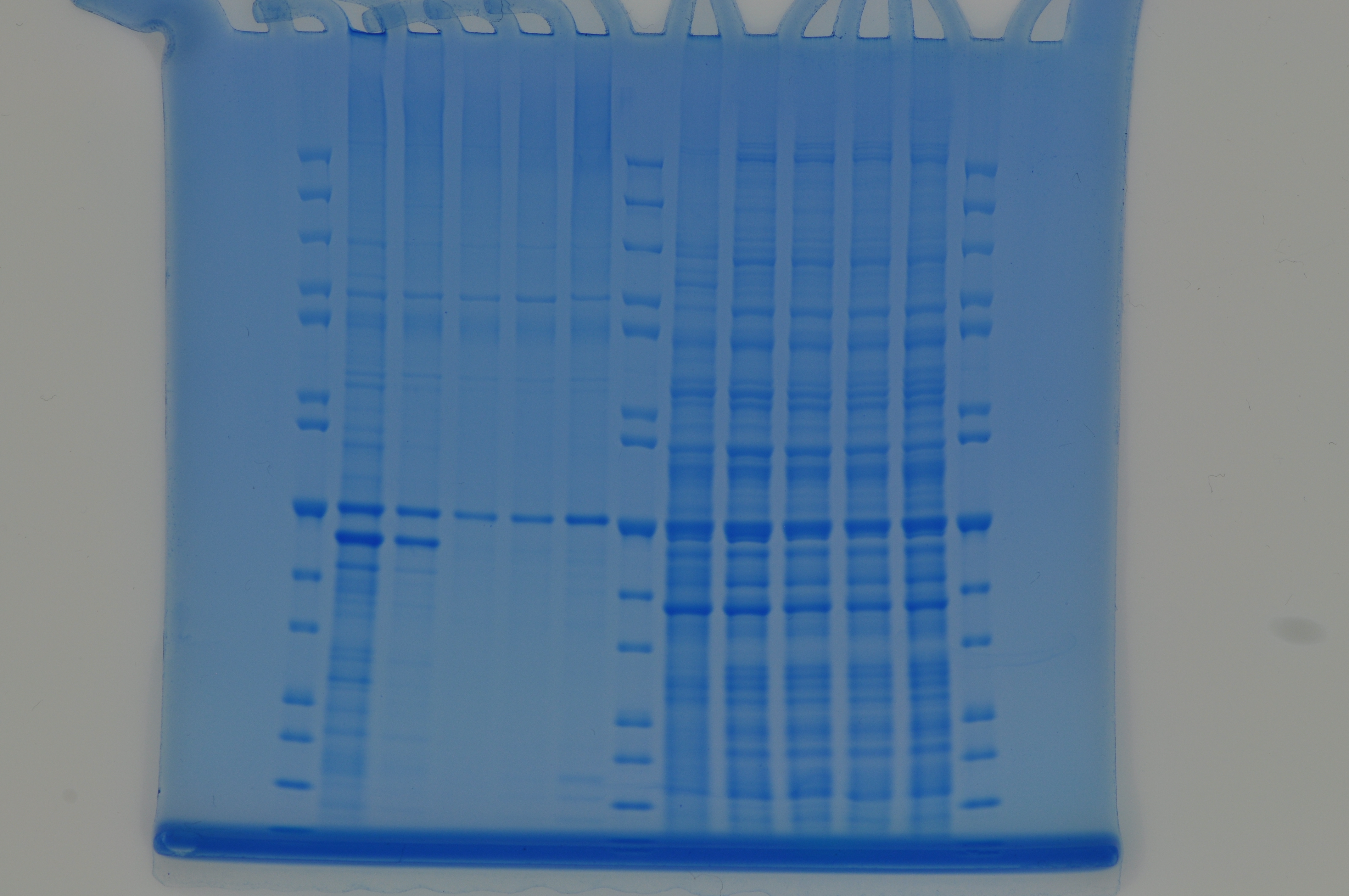

Supplement: Figure 7—figure supplement 4—source data 1. [file elife-74255-fig7-figsupp4-data1.zip › Figure 7-S4 - coomassie.JPG]

## Figure 7-S4 - source data

uncropped coomassie gel, original file: Figure 7-S4 - source.JPG

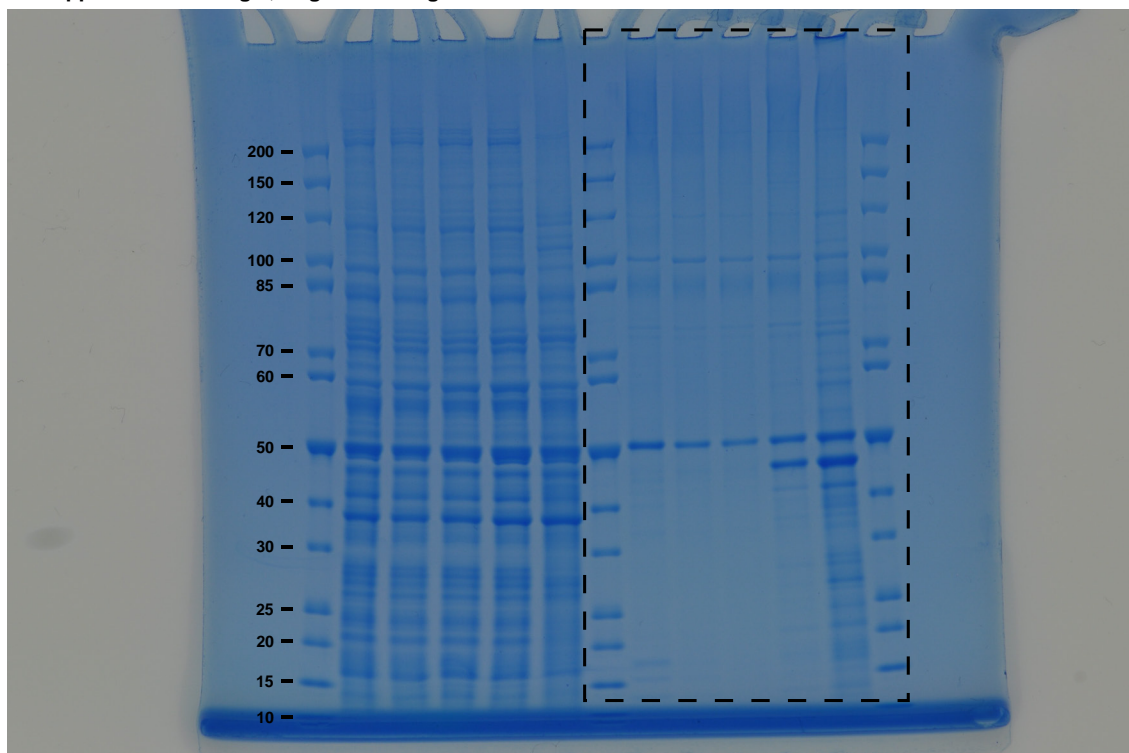

Supplement: Figure 7—figure supplement 4—source data 1. [file elife-74255-fig7-figsupp4-data1.zip › Figure 7-S4 - source data.pdf]
