# Supplementary material for: Dedicated chaperones coordinate co-translational regulation of ribosomal protein production with ribosome assembly to preserve proteostasis
Source: eLife. 2022 Mar 31;11:e74255. doi: 10.7554/eLife.74255 (PMC8970588; doi:10.7554/eLife.74255)

Figure 7D - source data

uncropped coomassie gel,
original file: Figure 7D - source coomassie.JPG

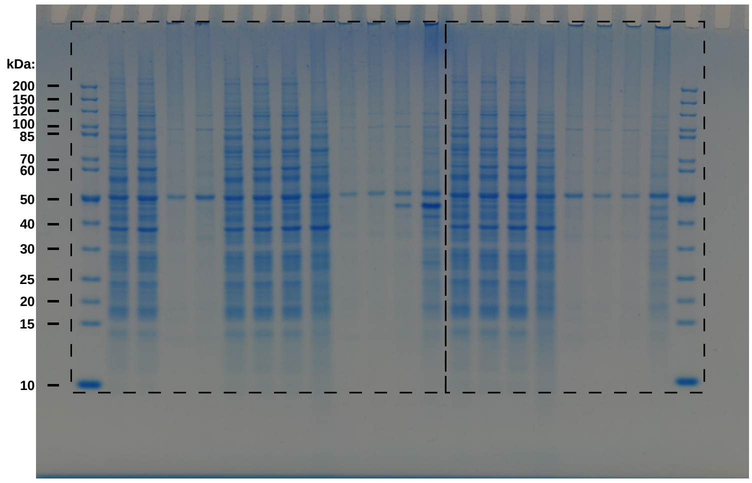

membrane 1:

uncropped blot

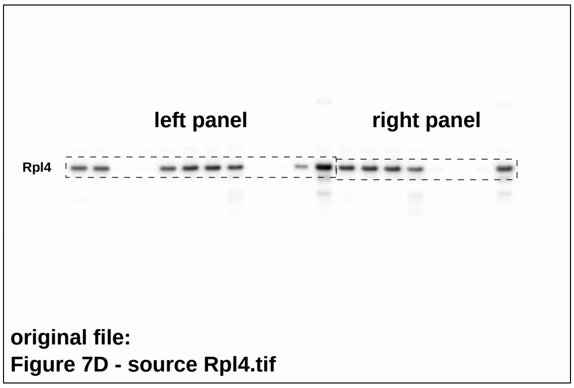

uncropped blot

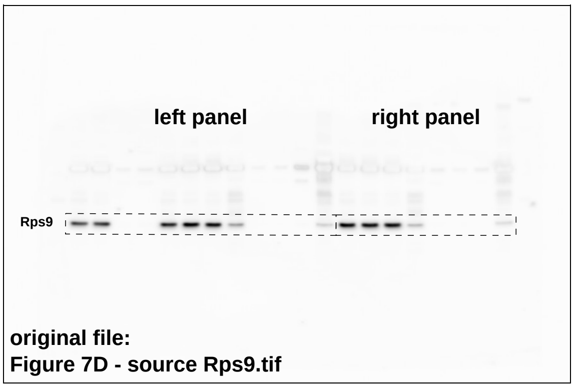

uncropped blot

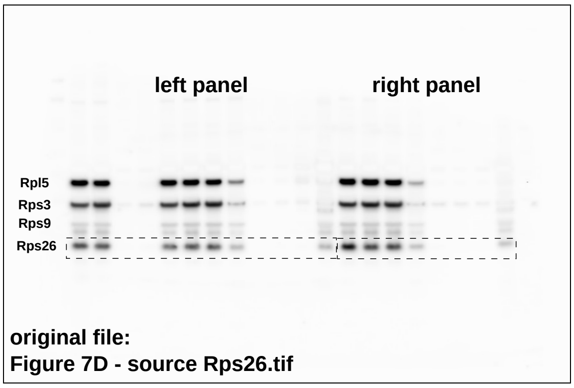

Figure 7D - source data

overlay of blot and membrane | uncropped blot

membrane 2:

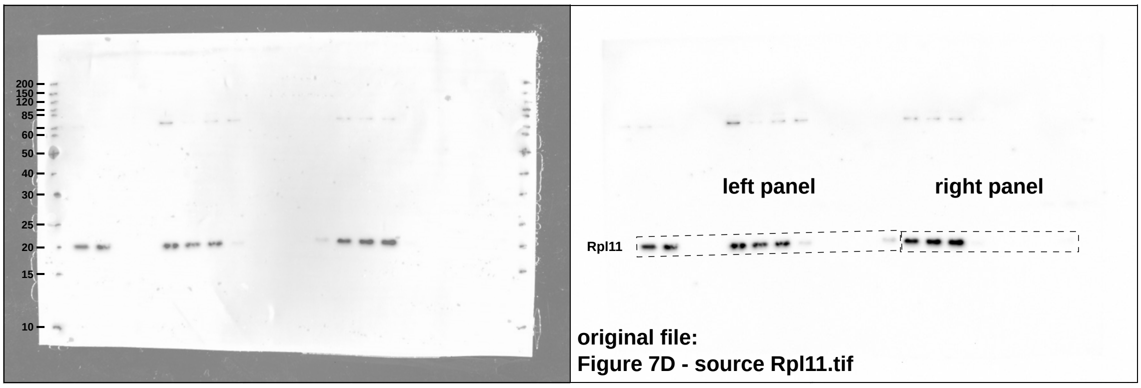

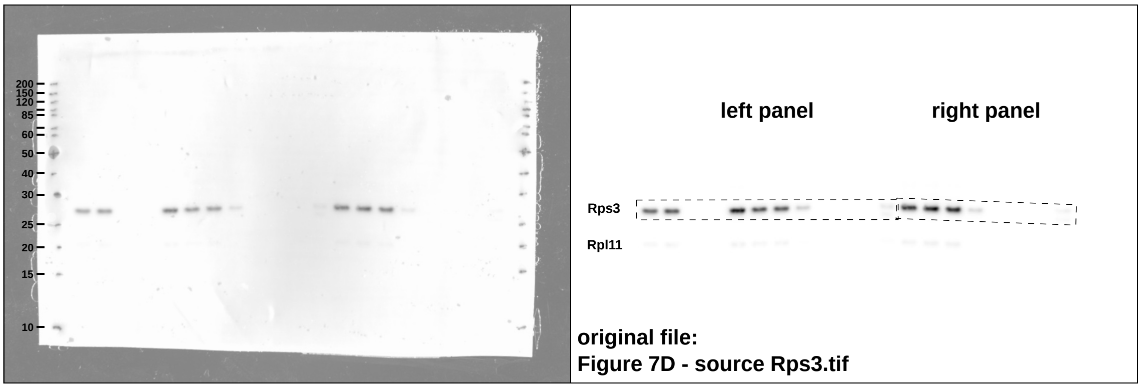

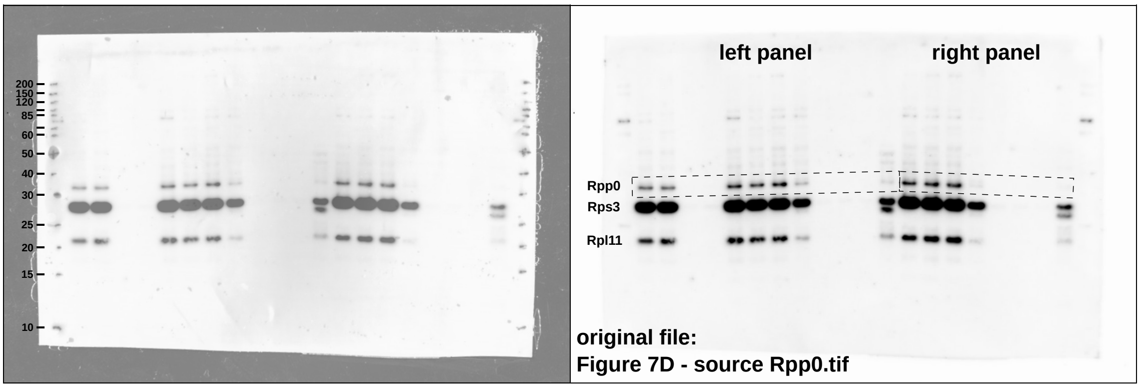

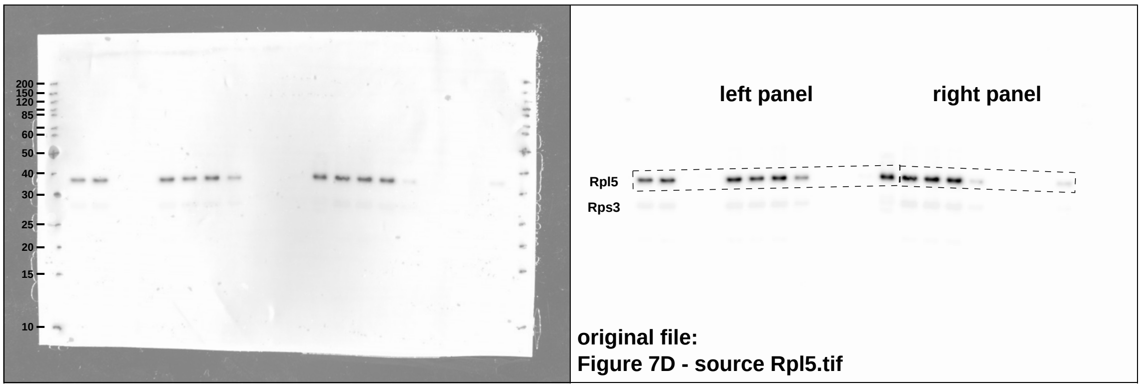

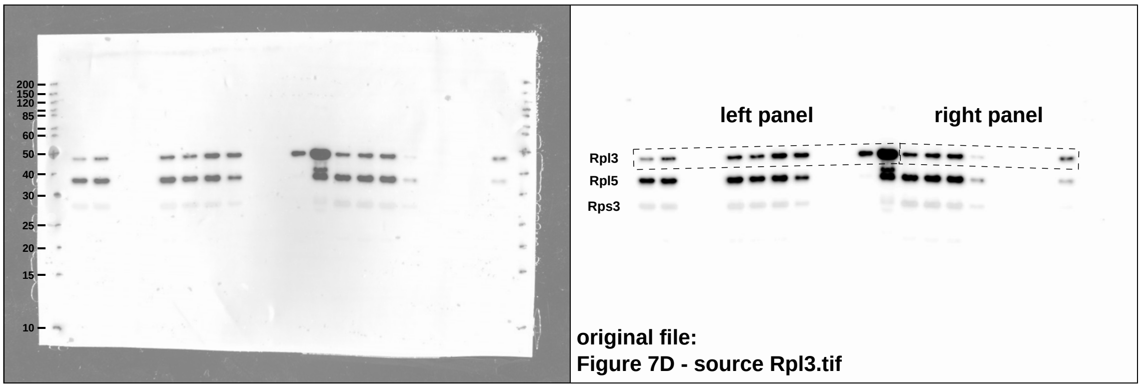

Figure 7D - source data

<u>membrane 3:</u>

overlay of blot and membrane | uncropped blot

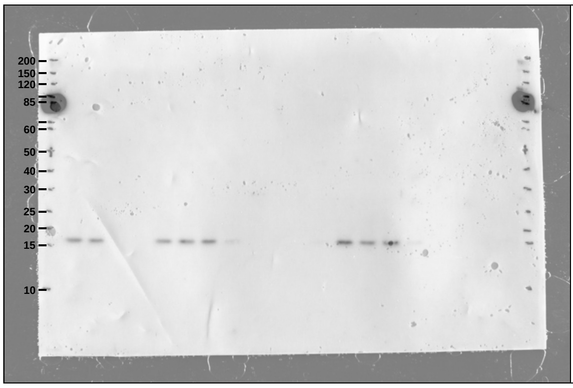

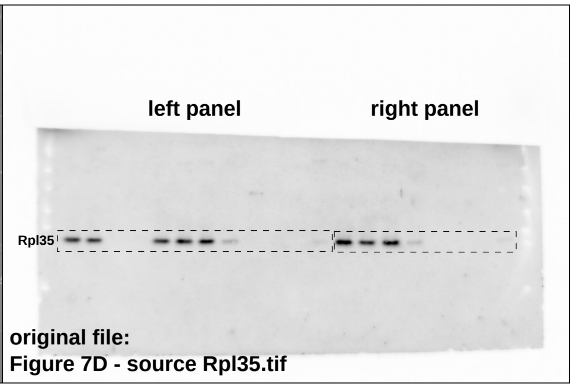

overlay of blot and membrane | uncropped blot

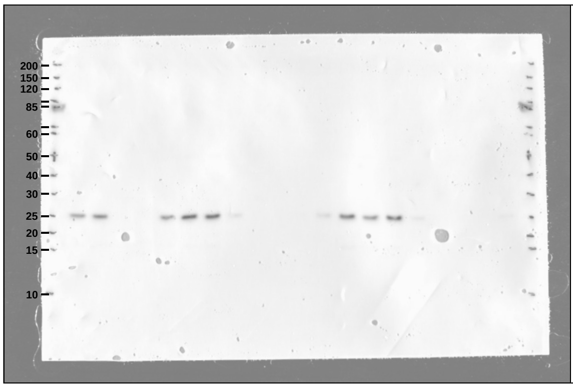

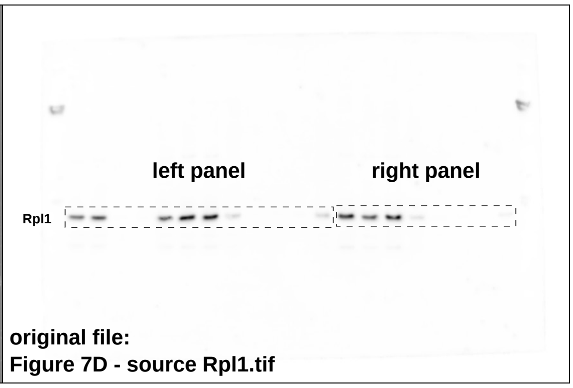

Supplement: Figure 7—source data 1. [file elife-74255-fig7-data1.zip › Figure 7D - source data.pdf]
